# In Situ TEM Studies of Tunnel‐Structured Materials for Alkali Metal‐Ion Batteries

**DOI:** 10.1002/advs.202500513

**Published:** 2025-04-15

**Authors:** Shuge Dai, Chenke Yang, Ye Wang, Yunrui Jiang, Longhui Zeng

**Affiliations:** ^1^ Key Laboratory of Material Physics of Ministry of Education, School of Physics Zhengzhou University Zhengzhou 450052 P. R. China; ^2^ Department of Electrical and Computer Engineering University of California San Diego La Jolla California 92093 USA

**Keywords:** alkali‐ion batteries, in situ transmission electron microscopy, reaction mechanism, tunnel structure

## Abstract

Tunnel‐structured materials have garnered significant attention as promising candidates for high‐performance rechargeable batteries, owing to their unique structural characteristics that facilitate efficient ionic transport. However, understanding the dynamic processes of ionic transport within these tunnels is crucial for their further development and performance optimization. Analytical in situ transmission electron microscopy (TEM) has demonstrated its effectiveness as a powerful tool for visualizing the complex ionic transport processes in real time. In this review, we summarize the state‐of‐the‐art in situ tracking of ionic transport processes in tunnel‐structured materials for alkali metal‐ion batteries (AMIBs) by TEM observation at the atomic scale, elucidating the fundamental issues pertaining to phase transformations, structural evolution, interfacial reactions and degradation mechanisms. This review covers a wide range of electrode and electrolyte materials used in AMIBs, highlighting the versatility and general applicability of in situ TEM as a powerful tool for elucidating the fundamental mechanisms underlying the performance of AMIBs. Furthermore, this work critically discusses current challenges and future research directions, offering perspectives on the development of next‐generation battery materials through advanced in situ characterization techniques.

## Graphical abstract

This review comprehensively investigates the latest advancements and key insights derived from in situ transmission electron microscopy (TEM) studies of tunnel‐structured materials in alkali metal‐ion batteries. By elucidating fundamental issues such as phase transformations, structural evolution, interfacial reactions, and degradation mechanisms, this work highlights the critical role of in situ TEM in advancing the understanding of these materials. Additionally, it highlights current challenges and future opportunities, providing valuable perspectives for the development of next‐generation battery technologies.

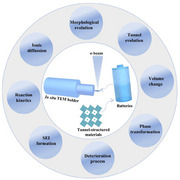

## 1 Introduction

The increasing demand for portable electronics, electric grids and electric vehicles has driven global efforts in large‐scale energy storage systems.^[^ ^1^, ^2^, ^3^, ^4^, ^5^ ^]^ Alkali metal‐ion batteries (AMIBs) have emerged as promising energy storage devices due to their high energy density and potential applications in electric vehicles and grid‐scale energy storage.^[^ ^6^, ^7^, ^8^, ^9^, ^10^ ^]^ However, the performance of AMIBs is primarily dependent on the structural and chemical dynamics of battery components (e.g. reaction mechanisms, structural evolution, ionic diffusion/migration, electrolyte degradation).^[^ ^11^, ^12^, ^13^, ^14^ ^]^ For battery materials, the electrode structures may inevitably undergo severe expansion and contraction during the charge/discharge reactions, thus resulting in the collapse or even pulverization of electrodes and capacity fading during repeated cycling.^[^ ^15^, ^16^ ^]^ Remarkably, the tunnel‐structured materials can provide a sufficiently robust framework for the fast insertion/deinsertion of charge carriers (e.g., Li^+^, Na^+^, K^+^, Zn^+^ and Mg^2+^).^[^ ^17^, ^18^, ^19^ ^]^ In particular, metal oxides/ sulfides (α‐MnO_2_, β‐FeOOH, TiO_2_, VO_2_ and KCu_7_S_4_) with an internal tunnel structure have been widely studied for batteries.^[^ ^20^, ^21^, ^22^, ^23^, ^24^, ^25^, ^26^ ^]^ Such tunnel structures can accommodate large charge carriers and enable fast ion diffusion inside the host materials. For instance, α‐MnO₂, characterized by its octahedral molecular sieve structure, features well‐ordered 1D 1 × 1 and 2 × 2 tunnels, which are large enough to facilitate rapid ion diffusion.^[^ ^17^ ^]^ Similarly, β‐FeOOH and TiO₂ exhibit tunnel structures that enhance ionic conductivity and structural stability during cycling.^[^ ^22^, ^23^, ^24^ ^]^ Although considerable efforts have been devoted to developing the tunnel‐structured materials, the underlying mechanism by which tunnels affect the insertion/extraction of charge carriers remains poorly understood. For example, the role of large cations (e.g., K⁺, Ag⁺, and NH_₄_⁺) in stabilizing tunnel structures and influencing ionic transport is still debated. Some studies suggest that these cations may impede ion diffusion due to physical blocking and repulsive electrostatic forces, while others argue that they help maintain tunnel integrity, preventing collapse during cycling.^[^ ^17^, ^27^ ^]^ Understanding these dynamic processes is crucial for improving the Coulombic efficiency, energy/power densities, stability and safety of AMIBs. Specifically, the ability of ionic transport plays a critical role in determining the charge/discharge rates and efficiency of AMIBs. By studying and optimizing ionic transport dynamics, researchers can design and optimize advanced electrode and electrolyte materials that facilitate faster ion transport, thus improving the rate capability, Coulombic efficiency, stability and longevity of rechargeable batteries. Notably, the dynamic evolution inside the working batteries had not been intensively studies until the advent of various in situ/operando characterization techniques.^[^ ^27^, ^28^ ^]^ Subsequently, these techniques have revolutionized the investigation of batteries, which provides direct observation and comprehensive study of the structural and chemical evolution of electrodes and interfaces during the dynamic charge/discharge processes, paving the way for advancements in battery technology and energy storage systems.

**Figure 1:**
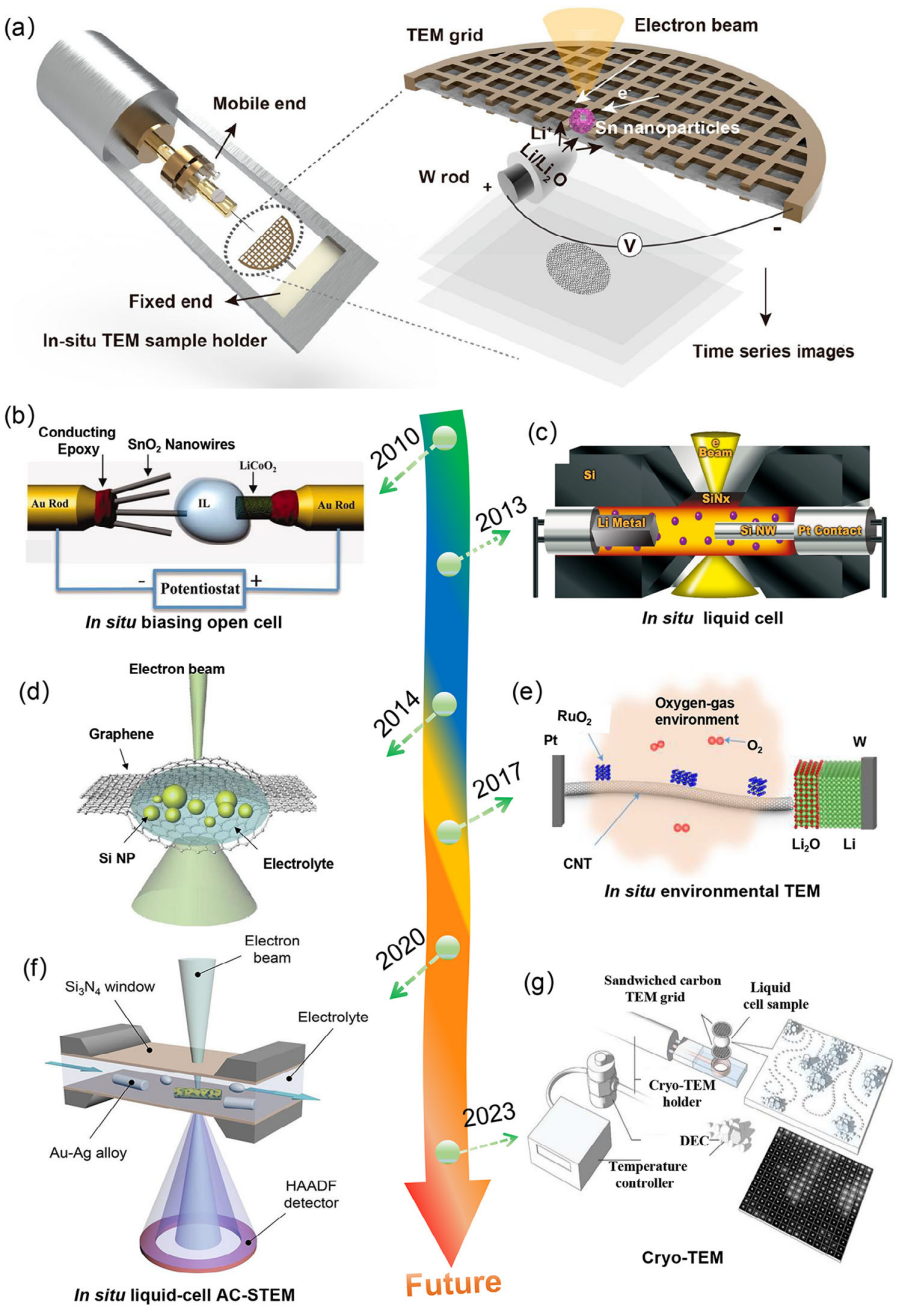
Timeline of the development of in situ TEM observations in batteries. a) In situ TEM sample holder and corresponding schematic illustration of in situ TEM experiment. Reproduced with permission.^[^ ^36^ ^]^ Copyright 2024, American Chemical Society. b) In situ biasing open cell. Reproduced with permission.^[^ ^50^ ^]^ Copyright 2010, American Chemical Society. c) Electrochemical liquid cell. Reproduced with permission.^[^ ^51^ ^]^ Copyright 2018, American Chemical Society. d) Graphene liquid cell. Reproduced with permission.^[^ ^53^ ^]^ Copyright 2014, American Chemical Society. e) In situ environmental TEM. Reproduced with permission.^[^ ^54^ ^]^ Copyright 2017, Springer Nature. f) In situ liquid‐cell AC TEM. Reproduced with permission.^[^ ^43^ ^]^ Copyright 2020, American Chemical Society. g) cry‐TEM. Reproduced with permission.^[^ ^57^ ^]^ Copyright 2023, American Chemical Society.

Nowadays, in situ/operando characterization techniques, including X‐ray diffraction (XRD), nuclear magnetic resonance (NMR), neutron diffraction (ND), atomic force microscopy (AFM), Raman spectroscopy and TEM, have been widely utilized to reveal the structural and chemical dynamics of electrode materials in battery systems.^[^ ^29^, ^30^, ^31^, ^32^, ^33^, ^34^, ^35^, ^36^, ^37^, ^38^ ^]^ These techniques provide invaluable information about the dynamic behavior of electrode materials and interfaces inside a working battery, which contributes to monitoring changes in crystal structure, phase transformations, surface reactions, and ion transport in real‐time, thus leading to more accurate predictions and a better understanding of battery performance.^[^ ^39^, ^40^, ^41^, ^42^, ^43^ ^]^ Among these techniques, in situ TEM observations are recognized as a powerful technique for characterizing electrode materials at the atomic scale.^[^ ^27^, ^28^ ^]^Recently, in situ TEM techniques have been developed to directly track the dynamic processes and underlying mechanisms of electrode materials during electrochemical reactions in real time.^[^ ^44^, ^45^, ^46^, ^47^, ^48^, ^49^ ^]^ Generally, an in situ TEM sample holder is a specialized device designed to hold and manipulate samples within the TEM while observing dynamic processes in real time. As shown in **Figure** **1**a, it typically consists of a holder stage that can be controlled to apply various stimuli, such as temperature, electrical bias, or mechanical deformation to the sample.^[^ ^36^ ^]^ Although in situ TEM technologies have been studied for several decades, they have been utilized in batteries for only a little over 10 years. A brief timeline is shown in Figure 1b–g. The first in situ nanocell battery was assembled with a SnO_2_ nanowire anode, a LiCoO_2_ cathode and an ionic liquid electrolyte (Figure 1b).^[^ ^50^ ^]^ Such an open‐cell nanobattery can effectively demonstrate the phase mechanisms and structural evolution of electrode materials during the charge/discharge process. Unfortunately, this designed open cell couldn't reveal the solid‐electrolyte interphase (SEI) layer due to its incompatibility with liquid electrolytes. Subsequently, Wang et al. designed an in situ TEM electrochemical liquid closed‐cell where the liquid electrolyte was filled in between the counter and working electrodes to simulate a realistic battery (Figure 1c).^[^ ^51^ ^]^ Unlike the open cell configurations, the closed‐cells can contribute to probing the dynamics of the electrolytes, SEI formation, and growth kinetics.^[^ ^51^ ^]^ Despite the great progress achieved, it cannot obtain high‐resolution images due to the inferior signal‐to‐noise ratio induced by SiN bulging.^[^ ^51^, ^52^ ^]^ To address this issue, a graphene liquid cell was first proposed by Lee's team,^[^ ^53^ ^]^ which can protect the sample against the vacuum within the chamber (Figure 1d). It exhibits the merit of easy‐accessibility without any in situ holders by the electron beam induced chemical charge/discharge. To understand the solid‐gas interface of fuel cells and metal‐air batteries, a new environmental TEM was developed by Wang's team in 2017,^[^ ^54^ ^]^ as shown in Figure 1e. This technology provides a powerful platform for revealing gas‐solid reaction, and atomistic imaging of the kinetic features in working batteries.^[^ ^39^, ^54^ ^]^ Notably, there have been limitations in effectively capturing both morphology visualization and mass information simultaneously in previous in situ TEM technologies. Recently, Liu et al. proposed a liquid‐cell aberration‐corrected scanning transmission electron microscopy (AC‐STEM), which can directly observe the structural evolution and the dynamic processes in liquid phases (Figure 1f).^[^ ^43^ ^]^ Owing to the limitation of low‐resolution TEM, the distribution of organic/inorganic components in the SEI layer and their effect on dendrite growth cannot be understood at the nanoscale.^[^ ^55^ ^]^ To address these challenges, a cryo‐transfer strategy based on cryo‐EM procedures was proposed by Cui's team.^[^ ^55^ ^]^ This technology can effectively probe the structure and composition of the SEI, as well as the structural and chemical mapping of solid‐liquid interfaces.^[^ ^55^, ^56^ ^]^ To achieve structural order in liquid electrolytes, Xie et al. proposed an integrated method of liquid‐phase TEM, cryo‐TEM operated at −30 °C, and 4D scanning TEM (Figure 1g).^[^ ^57^ ^]^ This technique enables the liquid electrolytes to remain in the liquid phase and mitigates electron beam damage.^[^ ^57^ ^]^ In short, in situ TEM techniques can offer not only visualization of working batteries but also help develop advanced battery systems beyond the limits of current techniques.

In this review, we aim to summarize the recent advancements and insights obtained from in situ TEM studies of tunnel‐structured materials in alkali metal‐ion batteries (AMIBs). Specifically, we will highlight the crucial role of in situ TEM studies in addressing the critical challenges faced by advanced AMIBs. These challenges include understanding the dynamic processes of ion transport, the structural evolution of electrode materials, and the degradation mechanisms. Through employing real‐time imaging and spectroscopic analysis, this review seeks to elucidate the intricate interplay between the electrochemical processes, material structure, and performance evolution in tunnel‐structured materials. We will discuss how in situ TEM observations have provided valuable information on the fundamental processes involved in working batteries, shedding light on the mechanisms governing ion transport, phase transitions, and degradation phenomena. Furthermore, this review will conclude by providing an overview of the potential perspectives and future opportunities that in situ TEM observations hold for AMIBs. By leveraging the capabilities of in situ TEM techniques, researchers can gain deeper insights into the dynamic behavior of multivalent‐ion systems, enabling the design of more efficient and stable electrode materials for next‐generation high‐energy‐density batteries.

## 2 In Situ TEM Studies of Tunnel‐Structured Materials for LIBs

In situ TEM studies have emerged as a pivotal tool for unraveling the intricate processes occurring within LIBs.^[^ ^58^, ^59^, ^60^, ^61^, ^62^ ^]^ These studies allow for real‐time visualization and analysis of the dynamic structural and chemical transformations that occur during the operation of lithium batteries.^[^ ^63^, ^64^, ^65^, ^66^ ^]^ By capturing atomic‐scale details, in situ TEM provides invaluable insights into the fundamental mechanisms governing the performance, degradation, and safety of lithium batteries.^[^ ^67^, ^68^, ^69^, ^70^, ^71^ ^]^ Based on the tunnel‐structured materials, we will elucidate the fundamental issues regarding the reaction mechanism, phase transformation, structural evolution, and performance degradation of LIBs.

In situ TEM studies of tunnel‐structured materials for LIBs provide valuable insights into the dynamic processes occurring within these materials during the lithiation and delithiation process. During lithiation/delithiation, in situ TEM can capture the process of lithium‐ion insertion/extraction from the tunnels of the materials, shedding light on the volume expansion, phase transitions, and formation of lithiated phases. This real‐time visualization helps elucidate the kinetics of lithium‐ion diffusion and the evolution of the material's nanostructure as it undergoes electrochemical reactions. Tunnel‐structured nanomaterials such as Mn‐based oxides,^[^ ^72^, ^73^, ^74^, ^75^, ^76^, ^77^ ^]^ Nb‐based oxides,^[^ ^78^, ^79^, ^80^, ^81^ ^]^ V‐based oxides,^[^ ^82^, ^83^, ^84^, ^85^, ^86^, ^87^ ^]^ Ti‐based oxides,^[^ ^88^, ^89^, ^90^ ^]^ etc. have garnered significant attention and application as electrode materials for LIBs because of their internal tunnels for free and fast ionic insertion and extraction. In this section, we will classify and summarize tunnel‐structured nanomaterials based on their roles as cathode and anode materials in LIBs. **Table** **1** comprehensively summarizes the electrochemical performances of cathode materials in LIBs.

**Table 1:** Summary of the electrochemical performances of cathode materials in LIBs.

Electrode material	Electrolyte	Rate performance	Discharge capability	Cycle capability	Ref.
Li_x_MnO_2_/LiPON/Li TFB	1 M LiClO_4_	50% retention (1.6 A g^−1^)	220 mAh g^−1^ (50 mA g^−1^)	200 cycles, 58.3% retention	[73]
Ti‐Doped MnO_2_ Nanowires	1 M LiPF_6_	31.9% retention (10 A g^−1^)	766 mA h g^−1^ (200 mA g^−1^)	3000 cycles, 96.86% retention	[91]
LiNi_0.5_Mn_1.5_O_4_ (LNMO)	1 M LiPF_6_	75.3% retention (8 A g^−1^)	507 mA h g^−1^ (80 mA g^−1^)	1000 cycles, 95% retention	[92]
Li_2_MnO_3_	1 M LiPF_6_	64.0% retention (0.9 A g^−1^)	_	6000 cycles, 85% retention	[93]
BT‐MnO_2_	1 M LiPF_6 _	_	236 mAh g^−1^ (10 mA g^−1^)	20 cycles, 73% retention	[94]
GT‐MnO_2_	1 M LiPF_6_	_	198 mAh g^−1^ (10 mA g^−1^)	20 cycles, 62% retention	[95]
Mn_2_P_2_O_7_–carbon@RGO	1 M LiPF_6_	45.4% retention (5 A g^−1^)	880 mAh g^−1^ (100 mAg^−1^)	150 cycles, 76.8% retention	[96]
MnO_2_@Co_3_O_4_	1 M LiPF_6_	41.5% retention (5 A g^−1^)	1734mAh g^−1^ (50 mA g^−1^)	400 cycles, 92.4% retention	[97]
Mo 5% δ‐MnO_2_	1 M LiPF_6_	45.9% retention (2 A g^−1^)	802.9 mAh g^−1^ (100 mA g^−1^)	100 cycles, 112.7% retention	[98]
MnO@C‐rGO	1 M LiPF_6_	58% retention (5 A g^−1^)	1162 mAh g^−1^ (200 mA g^−1^)	800 cycles, 110.4% retention	[85]
N‐V_2_O_3_	1.0 M LiPF_6_	56.2% retention (1 A g^−1^)	440.4 mA h g^−1^ (100 mA g^−1^)	1000 cycles, 100% retention	^[^ ^99^ ^]^
V_4_C_3_Tx‐BM‐HF	1 M LiPF_6_	14.5% retention (3 Ag^−1^)	185 mA h g^−1^ (100 mA g^−1^)	500 cycles, 121.6% retention	[100]
N‐S‐VCT‐600	1 M LiPF_6_	31.5% retention (5 A g^−1^)	849 m Ah g^−1^ (100 mA g^−1^)	100 cycles, 69.49% retention	[101]
V_2_CT_x_@SnO_2_	1 M LiPF_6_	_	2449.4 mAh g^−1^ (50 mA g^−1^)	200 cycles, 99.58% retention	[102]
V_2_O_3_@N‐C Nm	1 M LiPF_6_	38.% retention (2 A g^−1^)	1200 mAh g^−1^ (200 mA g^−1^)	1000 cycles, 81% retention	[103]
V_2_O_3_@C	1 M LiPF_6_	73.9% retention (2 A g^−1^)	700 mAh g^−1^ (100 mA g^−1^)	800 cycles, 100% retention	[104]
CV‐600	1 M LiPF_6_	47.2% retention (5 A g^−1^)	652.4 mAh g^−1^ (500 mA g^−1^)	_	[105]
S@G/G‐V_2_O_3_	1 M LITFSI	59.3% retention (5 A g^−1^)	1430 mAh g^−1^ (500 mA g^−1^)	1000 cycles, 53.6% retention	[106]
V_2_O_3_	1.1 M LiPF_6_	39% retention (2 A g^−1^)	200 mAh g^−1^ (100 mA g^−1^)	100 cycles, 85% retention	[107]
NC@V_2_O_3_	1 M LiPF_6_	53.4% retention (2 A g^−1^)	772 mAh g^−1^ (100 mA g^−1^)	200 cycles, 60.3% retention	[108]
Pure V_2_O_3_	1 M LiPF_6_	_	219 mAh g^−1^ (100 mA g^−1^)	200 cycles, 78.5% retention	[108]
V_2_O_3 _yolk–shell	1 M LiPF_6_	_	472.5 mAh g^−1^ (100 mA g^−1^)	100 cycles, 92.6% retention	[109]
LiFeHCF	1 M LiClO_4_	71.8% retention (1.9A g^−1^)	109 mAh g^−1^ (190 mA g^−1^)	650 cycles, 90% retention	[110]
Li_4_Fe(CN)_6_	1 M LiClO_4_	_	112 mAh g^−1^ (100 mA g^−1^)	20 cycles, 94.4% retention	[111]
Fe_4_(Fe(CN)_6_)_3_	1 M LiPF_6_	43.8% retention (0.24 A g^−1^)	450 mA h g^−1^ (8.76 mA g^−1^)	550 cycles, 101.3% retention	[112]

### 2.1 Cathode Materials

Tunnel‐structured cathode materials are critical for LIBs due to their ability to facilitate fast Li^+^ ion insertion/extraction and provide structural stability during cycling. Below, we will discuss key cathode materials studied using in situ TEM techniques.

#### 2.1.1 Mn‐based Oxides

**Figure 2:**
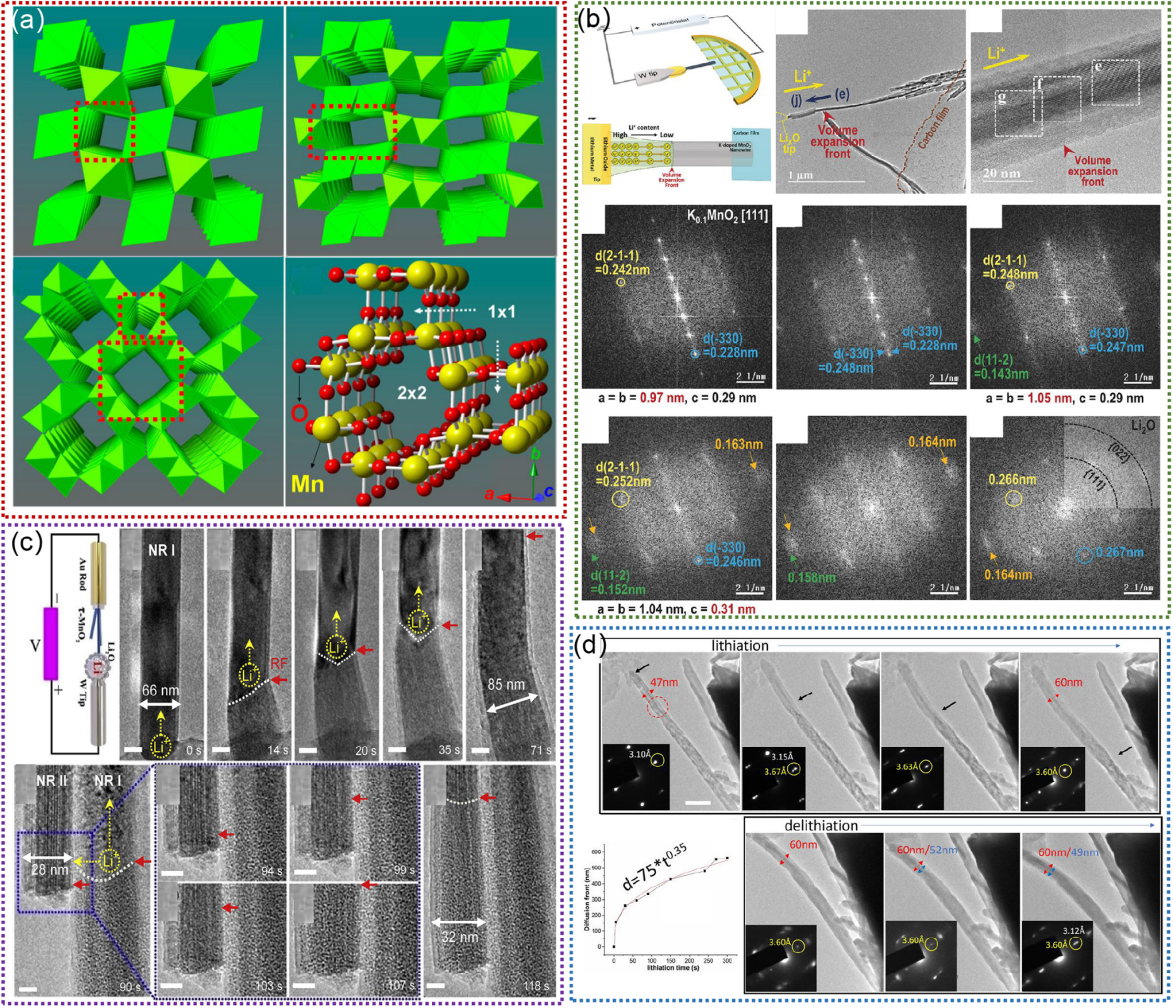
a) Crystal and atomic structures of MnO_2_. Reproduced with permission.^[^ ^74^ ^]^ Copyright 2015, American Chemical Society. b) In situ TEM experiment for the investigation of the lithiation mechanism of α‐MnO_2_ nanowires. Reproduced with permission.^[^ ^75^ ^]^ Copyright 2017, Wiley‐VCH GmbH. c) In situ visualization of lithium‐ion transport pathways of todorokite MnO_2_.^[^ ^76^ ^]^ Copyright 2019, Elsevier Ltd. d) Lithiation‐delithiation behavior of one MnO_2_ nanorod. Reproduced with permission.^[^ ^77^ ^]^ Copyright 2021, Wiley‐VCH GmbH.

Mn‐based oxides, (e.g., α‐MnO_2_, τ‐MnO_2_, β‐MnO_2_) are widely used as cathodes due to their tunneled structures (**Figure** **2**a), which facilitate the reversible insertion and extraction of lithium ions. Although remarkable achievements have been obtained, revealing the precise electrochemical reaction mechanisms occurring within these tunnel‐structured materials remains a challenge. In 2017, Lee. et al. investigated the lithiation mechanism of the tunnel‐structured α‐MnO_2_ nanowire by in situ high‐resolution TEM,^[^ ^75^ ^]^ as shown in Figure 2b. This research provides insight into the atomic position and diffusion pathway of lithium ions. Subsequently, Cai. et al. explored the (de)lithiation mechanisms of the todorokite‐type manganese oxide (τ‐MnO_2_) with p × 3 tunneled structure by in situ dynamic TEM, as illustrated in Figure 2c.^[^ ^76^ ^]^ This observation revealed the formation of Mn metal and Li_2_O phases through a Mn_2_O_3_ intermediate phase, demonstrating a stepwise intercalation‐conversion lithiation mechanism.^[^ ^76^ ^]^ This research has the potential to motivate endeavors aimed at achieving a comprehensive understanding of the highly polytypic material with other tunnel‐specific phases. Recently, He. et al. tracked the structure evolution of β‐MnO_2_ during the (de)lithiation process by in situ TEM (Figure 2d). The 1 × 1 tunnel framework of β‐MnO_2_ shows partially reversible expansion/contraction characteristics during lithiation/delithiation, during which an intermediate phase transition from β‐MnO_2_ to O‐LiMnO_2_ results in partial irreversibility.^[^ ^77^ ^]^ This work provides atomic‐level resolution of microscopic findings regarding the storage mechanisms of Li^+^ in β‐MnO_2_ for the first time.

**Figure 3:**
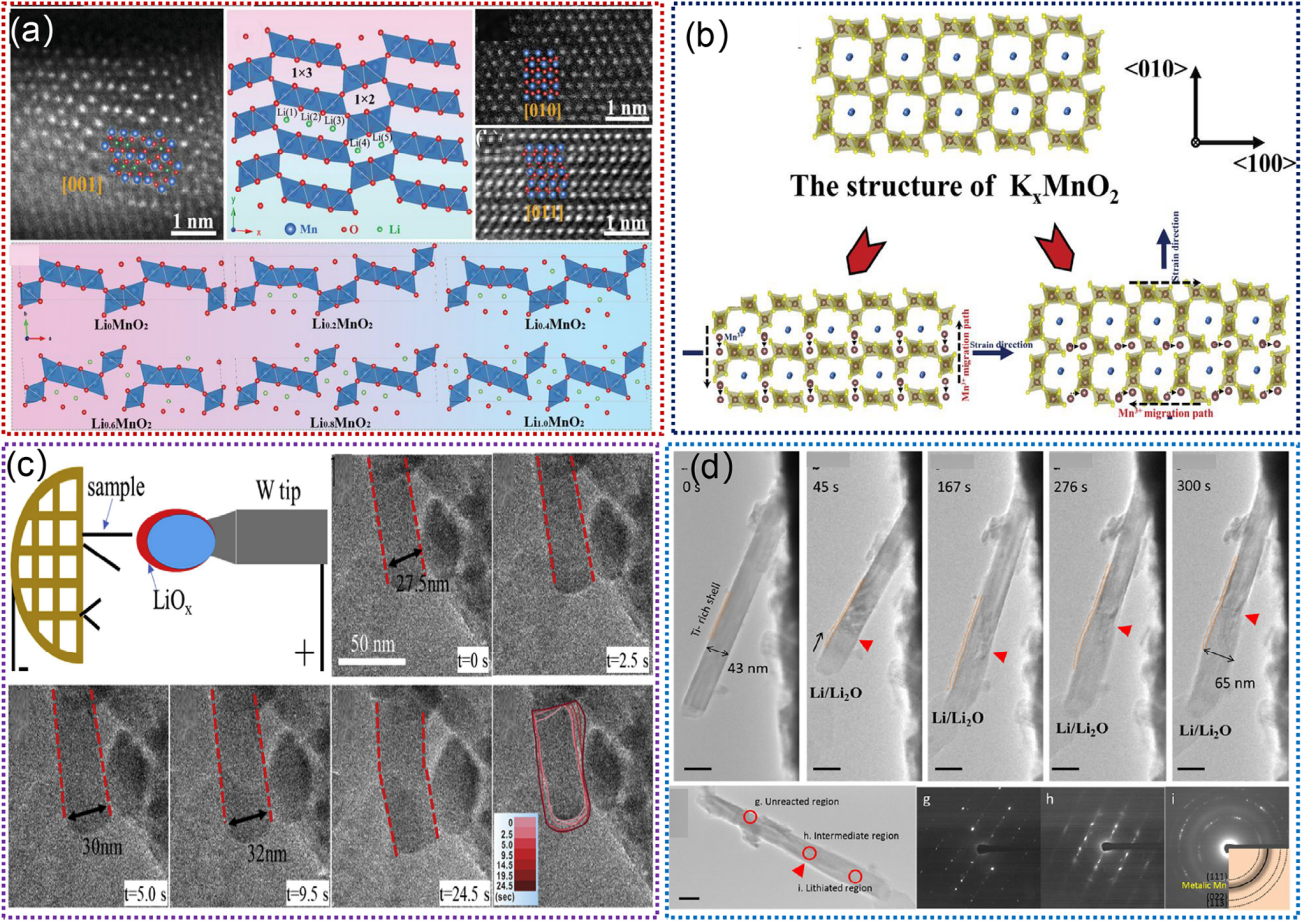
a) High‐angle annular dark field (HAADF)‐STEM images and simulated crystal structure model of the Li_x_MnO_2_. Reproduced with permission.^[^ ^73^ ^]^ Copyright 2020, Wiley‐VCH GmbH. b) The schematic illustration of the multi‐step reaction mechanism of K_x_MnO_2_. Reproduced with permission.^[^ ^113^ ^]^ Copyright 2022, Wiley‐VCH GmbH. c) In situ TEM experiment for the investigation of the lithiation mechanism of K_1.02_Mn_7.63_V_0.37_O_16_.^[^ ^24^ ^]^ Copyright 2020, Elsevier Ltd. d) In situ visualization of Ti‐MnO_2_ nanowires. Reproduced with permission.^[^ ^91^ ^]^ Copyright 2018 American Chemical Society.

To stabilize the tunnel framework structure and optimize the Li^+^ storage performance of MnO_2_, one of the effective strategies is introducing guest cation ions (Li^+^, K^+^, Ti^+^, Ag^2+^, etc.) into the tunnel structure.^[^ ^91^, ^113^, ^114^, ^115^ ^]^ These cations play pivotal roles in improving the properties of MnO_2_ structures, which not only reinforces the structural integrity by reducing lattice strain but also creates a more favorable environment for Li^+^ diffusion. Additionally, the interaction between guest cations and MnO_2_ can optimize the electronic structure, potentially enhancing the overall electrochemical performance. Delving deeper into the specific ion size, charge, and electronegativity can further illuminate their ultimate impact on performance metrics. For example, Xia. et al. reported that the pre‐insertion of Li^+^ ions into the α‐MnO_2_ could form an interesting Li_x_MnO_2_ tunnel structure with intergrowth of 1 × 3 and 1 × 2 tunnels,^[^ ^73^ ^]^ as illustrated in **Figure** **3**a. This tunnel structure exhibits a large charge storage capability, which is beneficial for accommodating a large amount of Li^+^ and enabling fast diffusion. Controlling the local distribution of K^+^ in the α‐MnO_2_ tunnels (Figure 3b) stabilized the α‐MnO_2_ tunnel structure.^[^ ^113^ ^]^ However the loss of K^+^ resulted in the phase transformation of α‐MnO_2_ into spinel Mn_3_O_4_, leading to the collapse of the tunnel structure.^[^ ^113^ ^]^ Yang. et al. utilized vanadium substitution of Mn in the tunnel wall, which enhances the structural uniformity of KMn_8_O_16_.^[^ ^24^ ^]^ Moreover, they demonstrated the V‐doped K_1.02_Mn_7.63_V_0.37_O_16_ can stabilize the tunnel framework structure during lithiation using in situ TEM observation, as shown in Figure 3c.^[^ ^24^ ^]^ Besides, Zhao. et al. revealed that Ti‐doping can also stabilize the tunnel framework structure of α‐MnO_2_ by in situ visualization of Ti‐MnO_2_ nanowires (Figure 3d).^[^ ^91^ ^]^ Overall, the incorporation of guest cations represents an effective strategy for optimizing the properties of MnO_2_ tunnel structures and improving their performance in various applications, particularly in the realm of energy storage.

#### 2.1.2 V‐Based Oxides

**Figure 4:**
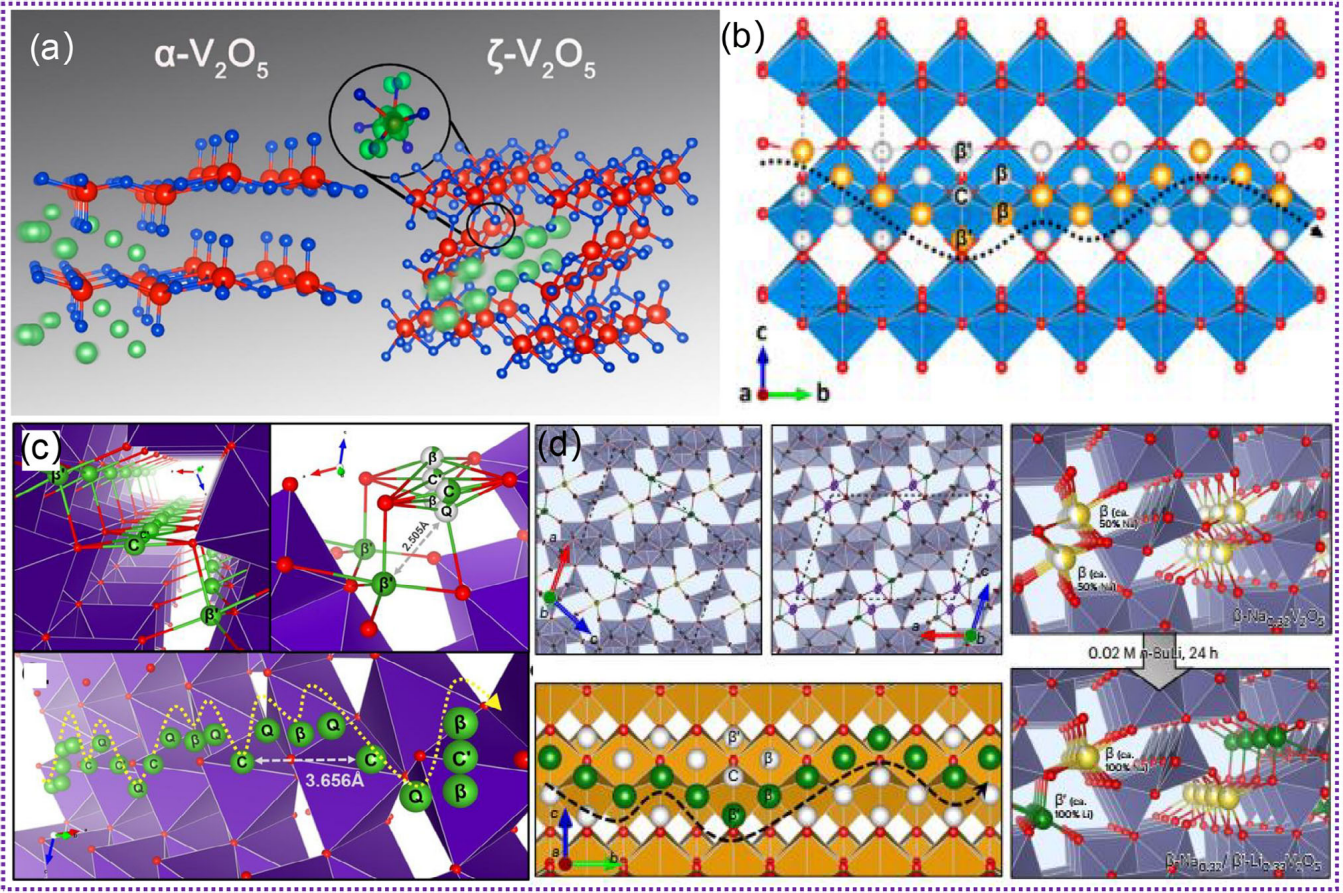
a) The simulated crystal structure model of the a‐V_2_O_5_ and ζ‐V_2_O_5_. Reproduced with permission.^[^ ^82^ ^]^ Copyright 2017, American Chemical Society. b) Schematic of the 1D tunnels of ζ‐V_2_O_5_ along different axes depicting the range of accessible interstitial sites. Reproduced with permission.^[^ ^83^ ^]^ Copyright 2023, American Chemical Society. c) Schematic of the 3D diffusion pathway of Li^+^ in Li_1.2_V_2_O_5_. Reproduced with permission.^[^ ^84^ ^]^ Copyright 2020, Wiley‐VCH GmbH. d) Single‐crystal XRD mapping of lithium‐ion diffusion in pre‐intercalated β‐Na_x_V_2_O_5_ and β‐K_x_V_2_O_5_. Reproduced with permission.^[^ ^87^ ^]^ Copyright 2024, Springer.

Tunnel‐structured vanadium oxides (ζ‐V_2_O_5_, Li_x_V_2_O_5_, Na_x_V_2_O_5_, K_x_V_2_O_5_. etc.) are promising electrode materials for LIBs due to their high abundance of interstitial sites and low diffusion barriers for Li^+^ migration.^[^ ^82^, ^83^, ^84^, ^85^, ^86^, ^87^ ^]^ Compared with a‐V_2_O_5_ (**Figure** **4**a), tunnel‐structured ζ‐V_2_O_5_ can provide ample locations for accommodating the insertion and extraction of Li^+^ during the charge/discharge cycles, facilitating high capacity and excellent cycle stability.^[^ ^82^ ^]^ Additionally, the accessibility of multi‐electron redox reactions on the vanadium centers further enhances the electrochemical performance. This feature allows for the transfer of multiple electrons per vanadium atom during the charge/discharge process, thus leading to higher energy storage capability and improved overall battery efficiency. Moreover, it also shows high theoretical capacity (441 mAh g^−1^), superior thermal and chemical stability, low‐stress accumulation upon cation insertion, and outstanding cyclability.^[^ ^83^, ^116^ ^]^ Figure 4b exhibits the accessible interstitial sites of Li^+^ in the tunnels of ζ‐V_2_O_5_ crystal, which reveals multiple interstitial sites along the 1D tunnel.^[^ ^83^ ^]^ Furthermore, the tunability of the V‐based oxides by varying the cation composition (e.g., Li, Na, K) provides a versatile platform for tailoring the electrochemical properties to meet specific performance requirements.^[^ ^84^, ^85^, ^86^, ^87^ ^]^ This adaptability opens up opportunities for customizing the electrode materials based on the desired characteristics, such as energy density, power density, and cycling stability, thereby offering a wide range of potential applications in the field of energy storage. Recently, Handy. et al. accurately identified Li‐ion sites and diffusion pathways in the tunnel of Li_1.2_V_2_O_5_ by operando powder X‐ray diffraction,^[^ ^84^ ^]^ as illustrated in Figure 4c. By examining topochemical insertion/extraction of Li^+^ in tunnel‐structured ζ‐V_2_O_5_ polymorph, they successfully captured the sequence of lattice interstitial sites filled (and emptied) by Li^+^ up to high depths of discharge.^[^ ^84^ ^]^ Furthermore, Luo. et al. mapped the Li^+^ site preferences and occupancies in pre‐intercalated β‐Na_x_V_2_O_5_ and β‐K_x_V_2_O_5_ by operando synchrotron X‐ray diffraction, as shown in Figure 4d.^[^ ^87^ ^]^ They elucidated the effect of pre‐intercalation in modifying the host lattice and altering the diffusion pathway.^[^ ^87^ ^]^ Although considerable efforts have been achieved, the underlying mechanism by which tunnel affects the insertion/extraction of Li^+^ is poorly understood by in situ TEM studies. Combined with in situ TEM, a more comprehensive understanding of the behavior of tunnel‐structured V‐based oxides in LIBs can be obtained. It can also contribute to a profound foundational understanding of the future development of high‐performance LIBs and inspire further exploration and improvement in electrode materials.

#### 2.1.3 Prussian Blue Analogues

**Figure 5:**
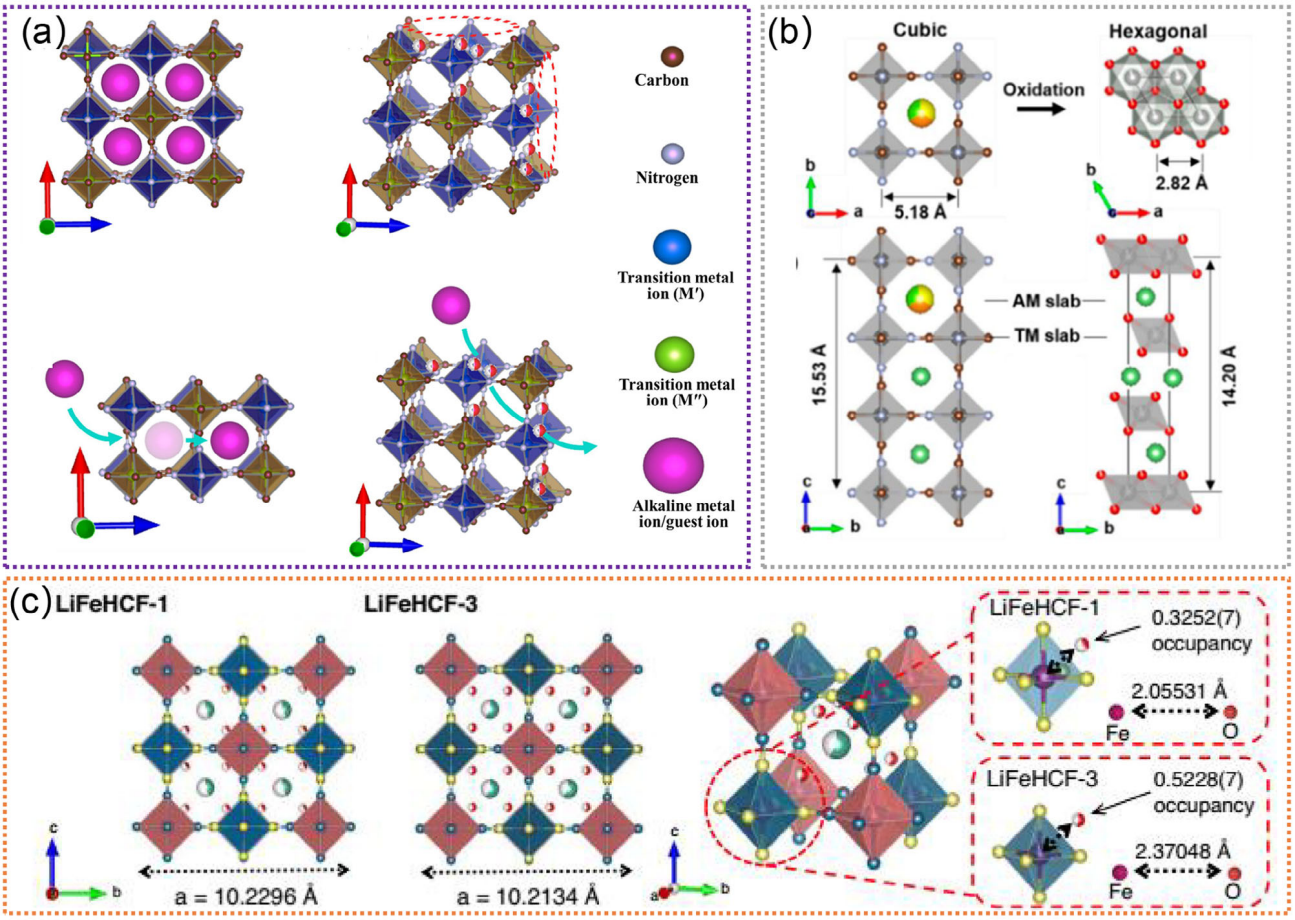
a) Schematic illustrations of Prussian blue analogs, and the insertion ions diffuse via^[^ ^99^ ^]^ channels. Reproduced with permission.^[^ ^117^ ^]^ Copyright 2021, Elsevier Ltd. b) Schemes of the lithiated Prussian blue analogs (left) and the LiNi_x_Co_y_Mn_z_O_2_ (right) unit cells along [001] and^[^ ^99^ ^]^ projections, respectively. Reproduced with permission.^[^ ^118^ ^]^ Copyright 2020, American Chemical Society. c) Comparison of the crystal cell and coordination environment between zeolitic water and Fe(CN)_6_ octahedra of LiFeHCF‐1 and LiFeHCF‐3 samples. Reproduced with permission.^[^ ^110^ ^]^ Copyright 2022, Springer.

Prussian blue analogs (PBAs) have garnered significant attention as electrode materials due to their ability to host a wide range of ions, making them suitable for deployment in various rechargeable battery systems.^[^ ^110^, ^117^, ^118^, ^119^, ^120^, ^121^, ^122^ ^]^ As shown in **Figure** **5**a, the 3D open framework of PBAs with large ionic channels enables highly reversible (de)intercalation of Li^+^ ions in non‐aqueous electrolytes, making them versatile candidates for LIBs.^[^ ^117^ ^]^ Unfortunately, the activation of only one transition‐metal species during the charge/discharge process limits the full utilization of the PBA's redox capabilities, leading to underutilization of the framework's potential for energy storage. Interestingly, Park et al. proposed a new LiNi_x_Co_y_Mn_z_O_2_ (NCM) material by a chemical lithiation process (Figure 5b), which displays a high discharge capacity of 222 mAh g^−1^ at 0.1 C.^[^ ^118^ ^]^ As‐prepared NCM exhibits a LiO_2_ slab space of 2.637 Å close to the ideal value of 2.64 Å, thus leading to favorable structural stability and reversibility during the long charging/discharging cycles.^[^ ^118^ ^]^ This finding highlights the critical role of structural control and order within the material in achieving enhanced energy density and overall performance in LIB applications. Recently, Zhang et al. reported a lithium‐containing Prussian blue hexacyanoferrate material (LiFeHCF), which shows a high discharge capacity of 142 mAh g^−1^ at 19 mA g^−1^.^[^ ^110^ ^]^ As displayed in Figure 5c, the LiFeHCF‐1 exhibits a lattice parameter increase from 10.2134 to 10.2296 Å in comparison to LiFeHCF‐3, which indicates that there is more space available for Li^+^ ions to move within the crystal structure of LiFeHCF‐1, facilitating enhanced mobility and diffusion of Li^+^ ions.^[^ ^110^ ^]^ Such findings underscore the potential for optimizing the structure of lithium‐containing Prussian blue hexacyanoferrate materials to facilitate improved Li^+^ ion transport, which is crucial for enhancing the performance of LIBs. It is generally thought that 3D open framework of PBAs plays a significant role in the operation of LIBs. However, the precise reaction mechanisms occurring within this structure are not yet fully understood by some effective characterization techniques. Continued research efforts that utilize advanced characterization methods such as in situ TEM, XRD, and other analytical tools are essential for unraveling the intricacies of the reaction mechanisms occurring within the 3D open framework of PBA materials. By employing these sophisticated techniques, researchers can gain real‐time insights into the structural and chemical changes that take place during the operation of LIBs, leading to a more comprehensive understanding of the underlying processes. This, in turn, paves the way for the development of improved energy storage materials and technologies.

### 2.2 Anode Materials

Tunnel‐structured anode materials are essential for accommodating large volumes of Li^+^ ions and maintaining structural integrity during cycling. Below, we discuss key anode materials studied using in situ TEM techniques. **Table** **2** comprehensively summarizes the electrochemical performances of anode materials in LIBs.

**Table 2:** Summary of the electrochemical performances of anode materials in LIBs.

Electrode material	Electrolyte	Rate performance	Discharge capability	Cycle capability	Refs.
Ti_2_Nb_10_O_29_ cages	1 M LiPF_6_	44.4% retention (30 A g^−1^)	302.5 mA h g^−1^ (100 mA g^−1^)	_	[78]
H_2_TiNb_6_O_18_	1 M LiPF_6_	27% retention (1 A g^−1^)	18 mAh g^−1^ (20 mA g^−1^)	_	[79]
RS‐Nb_2_O_5_	1.2 M LiPF_6_	70% retention (1 A g^−1^)	269 mAh g^−1^ (20 mA g^−1^)	400 cycles, 100% retention	[121]
Nb_2_O_5_@C	1 M LiPF_6_	22% retention (20 A g^−1^)	240 mAh g^−1^ (100 mA g^−1^)	800 cycles, 109% retention	[122]
MSC‐Nb_2_O_5_	1 M LiPF_6_	_	270 mAh g^−1^ (50 mA g^−1^)	10 cycles, 48% retention	[123]
Nb_14_W_3_O_44_	1 M LiPF_6_	38.1% retention (8.9 A g^−1^)	249.2 mAh g^−1^ (89 mA g^−1^)	200 cycles, 98% retention	[124]
Co‐Nb_2_O_5_	1 M LiPF_6_	58.6% retention (5 A g^−1^)	256.1mAh g^−1^ (100 mA g^−1^)	500 cycles, 90% retention	[125]
T‐Nb_2_O_5_	1 M LiPF_6_	105% retention (0.1 A g^−1^)	435.1mAh g^−1^ (100 mA g^−1^)	200 cycles, 101% retention	[126]
a‐Nb_2_O_5_	1.2 M LiPF_6_	43% retention (1 A g^−1^)	243 mAh g^−1^ (200 mA g^−1^)	400 cycles, 85% retention	[127]
TiO_2_/MXene	1 M LiPF_6_	32.5% retention (1 A g^−1^)	169mAh g^−1^(50 mA g^−1^)	_	[128]
SC‐l Ti_3_C_2_(OH)_2_	1 M LiPF_6_	_	242 mA g^−1^ (100 mA g^−1^)	250 cycles, 36.7% retention	[129]
Al‐Nb_2_O_5_@NC	1 M LiPF_6_	25.1% retention (20 A g^−1^)	240 mAh g^−1^ (100 mA ^−1^)	9900 cycles, 78% retention	[130]
N‐C@MSC‐Nb_2_O_5_	1 M LiPF_6_	48% retention (16 A g^−1^)	270 mAh g^−1^ (50 mA g^−1^)	1000 cycles, 83% retention	[131]
In_0.5_Nb_24.5_O_62_‐F90‐6	1 M LiPF_6_	37.9% retention (2.42 A g^−1^)	199.2mAh g^−1^ (12.1 mA g^−1^)	200 cycles, 114% retention	[132]
Ru‐doped Nb_2_O_5_	1 M LiPF_6_	58.9% retention (16 A g^−1^)	257.9 mAh g^−1^ (200 mA g^−1^)	3000 cycles, 58% retention	[133]
Nb_2_O_5_‐AIB30	1 M LiPF_6_	16.7% retention (10 A g^−1^)	211 mAh g^−1^ (40 mA g^−1^)	300 cycles, 80% retention	[134]
Nb_2_O_5_@TiO_2_c‐13	1 M LiPF_6_	68% retention (5 A g^−1^)	232.1 mAh g^−1^ (100 mA g^−1^)	1000 cycles, 88% retention	[135]
Nb_2_O_5_@NC	1 M LiPF_6_	61.% retention (6 A g^−1^)	224 mAh g^−1^ (100 mA g^−1^)	2000 cycles, 67% retention	[136]
TiO_2_− x–C–Sn	1 M LiPF_6_	35.6% retention (5 A g^−1^)	1562 mAh g^−1^ (100 mA g^−1^)	200 cycles, 61% retention	[137]
SnS/Ti_3_C_2_T_x_	1 M LiPF_6_	76.8% retention (5 A g^−1^)	1254 mAh g^−1^ (100 mA g^−1^)	180 cycles, 96% retention	[138]
LTP‐ TiO_2_/MXene	1 M LiPF_6_	46% retention (1 A g^−1^)	466mAh g^−1^ (50 mA g^−1^)	500 cycles, 95% retention	[139]
RDS/d‐Ti_3_C_2_T_x_	1 M LiPF_6_	_	457 mAh g^−1^ (50 mA g^−1^)	500 cycles, 107% retention	[140]
P‐Ti_3_C_2_	1 M LiPF_6_	41.1% retention (0.8 A g^−1^)	310 mAh g^−1^ (100 mA g^−1^)	3000 cycles, 100% retention	[141]
Ti_3_C_2_T_x_‐T nanosheets	1 M LiPF_6_	75.6% retention (1 A g^−1^)	440 mAh g^−1^ (100 mA g^−1^)	200 cycles, 110% retention	[142]
**V0**._2_‐Ti_3_C_2_T_x_	1 M LiPF_6_	47.1% retention (5 A g^−1^)	251.3 mAh g^−1^ (100 mA g^−1^)	180 cycles, 69.% retention	[143]
N–Ti_3_C_2_T_x_/P	1 M LiPF_6_	50% retention (2 A g^−1^)	1160 mAh g^−1^ (100 mA g^−1^)	200 cycles, 75% retention	[144]
Co_3_O4/Ti_3_C_2_T_x3_	1 M LiPF_6_	105% retention (0.05 A g^−1^)	611.9 mA h g^−1^ (500 mA g^−1^)	900 cycles, 242% retention	[145]
Ti_3_C_2_T_x_/CNTs@P	1 M LiPF_6_	27.3% retention (52A g^−1^)	2598 mA h g^−1^ (130mA g^−1^)	500 cycles, 83% retention	[146]
Li‐Nb_2_CT_x_‐400	1 M LiPF_6_	_	985mAh g^−1^ (50 mA g^−1^)	2000 cycles, 75% retention	[147]
Ni(OH)_2/d_‐Ti_3_C_2_	1 M LiPF_6_	55.6% retention (2 A g^−1^)	615.2mAh g^−1^ (100 mA g^−1^)	1000 cycles, 106% retention	[148]
Sn@Ti_3_C_2_	1 M LiPF_6_	29.6% retention (3 A g^−1^)	1039mAh g^−1^ (500 mA g^−1^)	250 cycles, 64% retention	[149]
Ti_3_C_2_(OH)_2_ NRs	1 M LiPF_6_	27.3% retention (1 A g^−1^)	292.4mAh g^−1^ (100 mA g^−1^)	250 cycles, 49% retention	[150]
in‐Ti_3_C_2_	1 M LiPF_6_	_	123.6mAh^−1^ (260 mA g^−1^)	75 cycles, 96% retention	[151]
p‐Ti_3_C_2_T_x_/CNT	1 M LiPF_6_	26.4% retention (3.2 A g^−1^)	1250mAh g^−1^ (160 mA g^−1^)	_	[152]
PVP‐Sn(IV)@Ti_3_C_2_	1 M LiPF_6_	30.4% retention (3 A g^−1^)	1487 mAh g^−1^ (100 mA g^−1^)	500 cycles, 94% retention	[153]

#### 2.2.1 Nb‐Based Oxides

**Figure 6:**
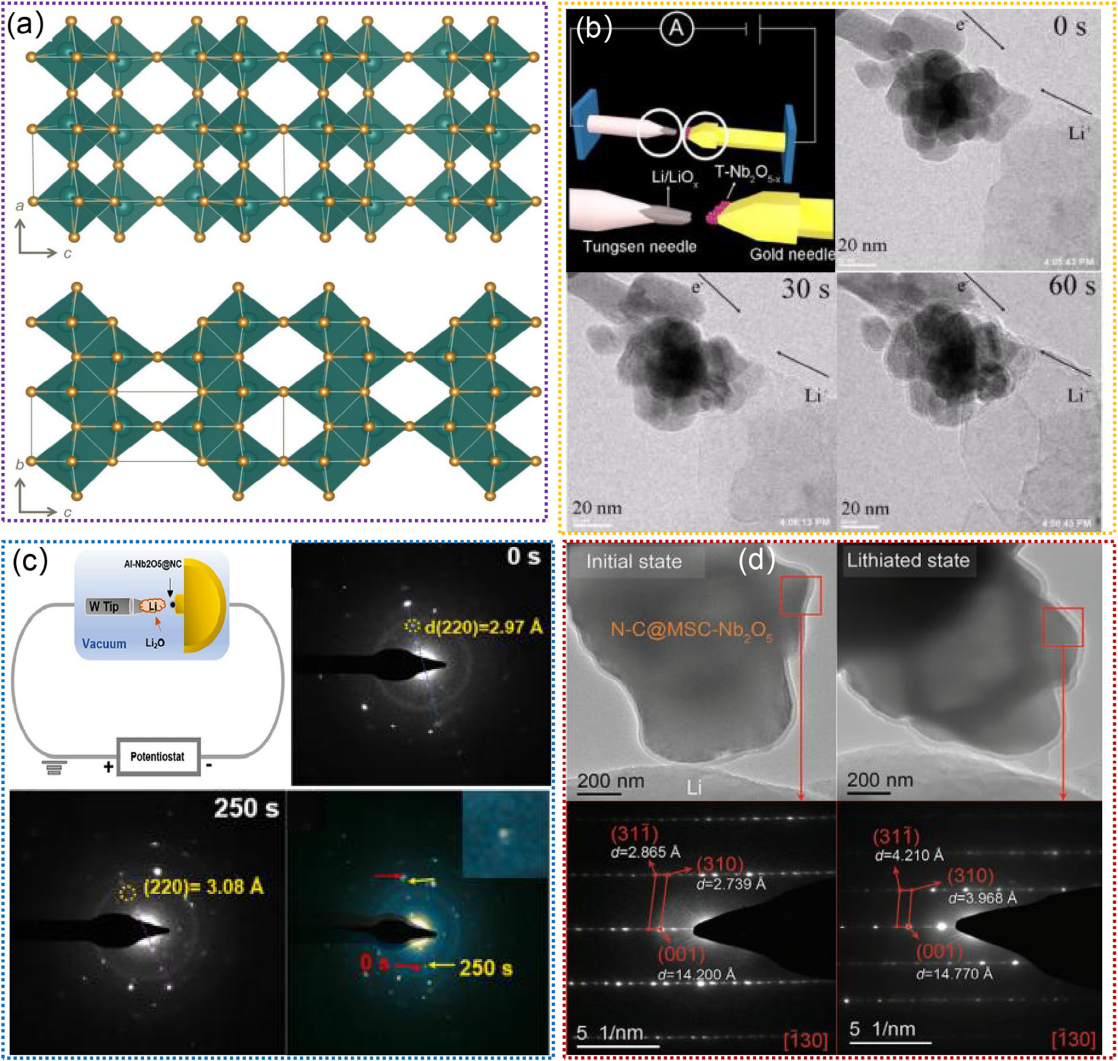
a) The simulated crystal structure model of the R‐Nb_2_O_5_. Reproduced with permission.^[^ ^128^ ^]^ Copyright 2023, The Royal Society of Chemistry. b) In situ TEM experiment for the investigation of the lithiation mechanism of T‐Nb_2_O_5‐x_. Reproduced with permission.^[^ ^129^ ^]^ Copyright 2021, Elsevier Ltd. c) Evolution of morphology and microstructure of Al‐Nb_2_O_5_@NC during lithiation by in situ TEM. Reproduced with permission.^[^ ^130^ ^]^ Copyright 2023, Elsevier Ltd. d) The in situ TEM images and corresponding SAED patterns of N‐C@MSC‐Nb_2_O_5_ particles. Reproduced with permission.^[^ ^131^ ^]^ Copyright 2020, Wiley‐VCH GmbH.

Nb‐based oxides, such as Nb_2_O_5_, have been extensively studied as anode materials for LIBs because their unique tunnel structures facilitate the fast insertion and extraction of Li^+^.^[^ ^121^, ^122^, ^123^, ^124^, ^125^ ^]^ Such internal tunnels provide convenient pathways for Li^+^ migration and diffusion, thus allowing for efficient and reversible electrochemical reactions.^[^ ^126^, ^127^, ^128^, ^129^ ^]^ Additionally, the tunnel‐structured Nb_2_O_5_ offers a high degree of structural stability (**Figure** **6**a), which is essential for prolonged cycling performance and overall durability in LIBs. To reveal the precise electrochemical reaction mechanisms occurring within these tunnels, in situ TEM has been considered as a powerful technique for visualizing their tunnels during the (de)lithiation processes. For example, Yan. et al. confirmed the small volume expansion and phase‐boundary‐free process of T‐Nb_2_O_5‐x_ during the lithiation process by using in situ TEM, as illustrated in Figure 6b.^[^ ^129^ ^]^ This structure shows a simultaneously low barrier for electron and ion transfer. To improve the performance of T‐Nb_2_O_5_, numerous efforts have been focused on atomic doping or forming a composite with carbon materials.^[^ ^130^, ^131^, ^132^, ^133^ ^]^ Zhu. et al. designed the aluminum‐doped T‐Nb_2_O_5_ embedded in the N‐doped carbon network (Al‐Nb_2_O_5_@NC).^[^ ^130^ ^]^ By in situ TEM (Figure 6c), they revealed the intercalation‐type electrochemical reaction and good crystalline integrity of Al‐Nb_2_O_5_@NC. Song. et al. reported a high‐performance N‐C@MSC‐Nb_2_O_5_ (micrometer single‐crystal H‐Nb_2_O_5_) electrode by introducing an amorphous N‐doped carbon (N‐C) shell. According to in situ TEM observation (Figure 6d), they found that the open diffusion tunnel of Li^+^ is parallel with the orientation of structural expansion, which could retain straight rather than twists during (de)lithiation processes.^[^ ^131^ ^]^ Overall, tunnel‐structured Nb‐based oxides show promising potential for applications in LIBs, which also present a compelling avenue for further research and development in the field of advanced energy materials.

#### 2.2.2 Ti‐Based Oxides

**Figure 7:**
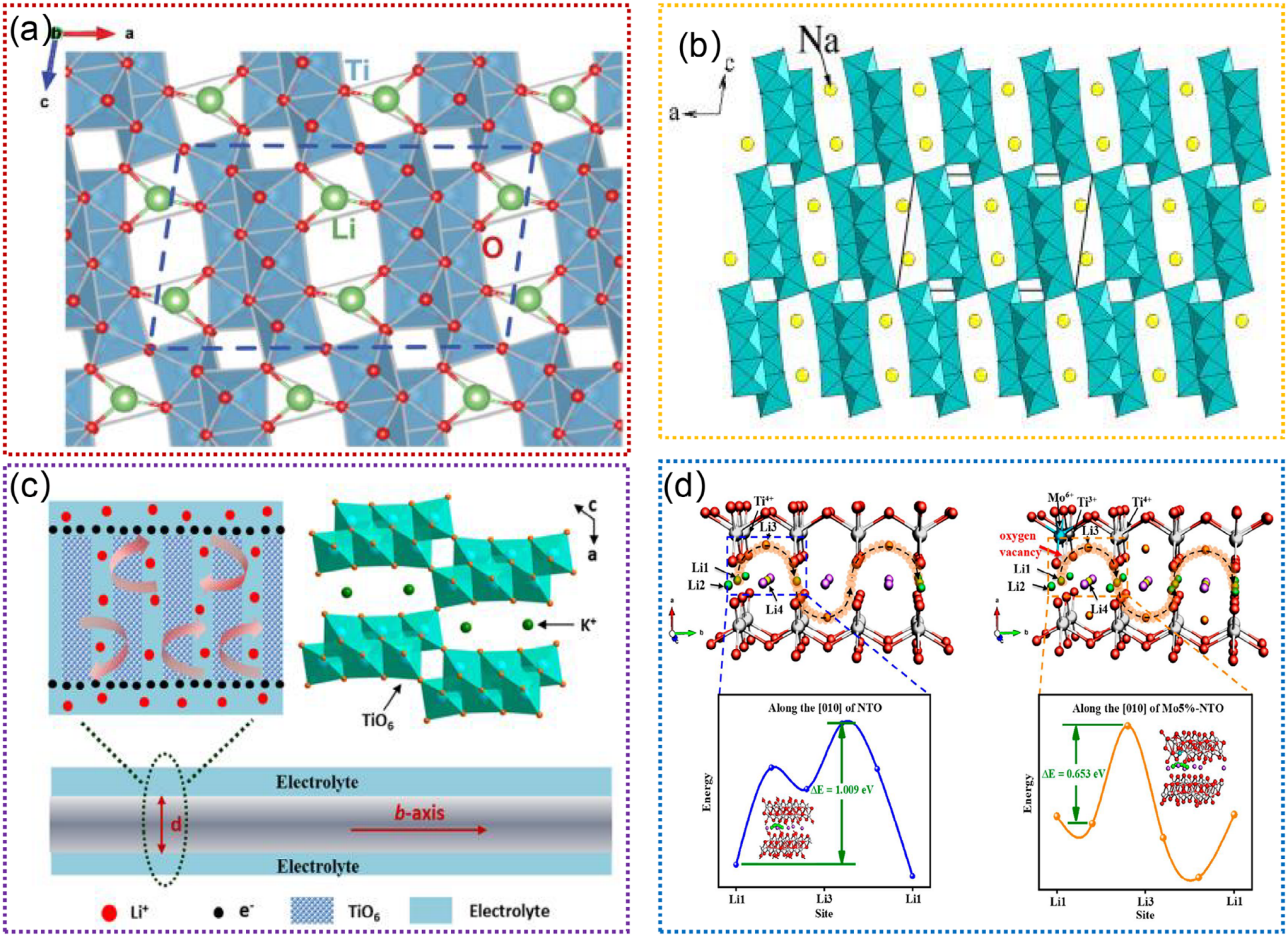
a) The simulated crystal structure model of the Li_2_Ti_6_O_13_. Reproduced with permission.^[^ ^157^ ^]^ Copyright 2018, AIP Publishing. b) Schematic of the crystal structure of Na_2_Ti_6_O_13_ (octahedral:Ti‐O_6_, spheres:Na). Reproduced with permission.^[^ ^158^ ^]^ Copyright 2015, Elsevier Ltd. c) Structure schematic and transport path of Li^+^ and electron in K_2_Ti_6_O_13_. Reproduced with permission.^[^ ^159^ ^]^ Copyright 2020, Elsevier Ltd. d) The structural models and energy diffusion paths of Li^+^ in Na_2_Ti_6_O_13_ and Mo5%‐Na_2_Ti_6_O_13_. Reproduced with permission.^[^ ^89^ ^]^ Copyright 2023, Elsevier Ltd.

Ti‐based oxides (e.g., Li_2_Ti_6_O_13_, Na_2_Ti_6_O_13_, K_2_Ti_6_O_13_) are promising anodes due to their low volume expansion and high structural stability,^[^ ^154^, ^155^, ^156^, ^157^, ^158^, ^159^ ^]^ which facilitate the reversible insertion and extraction of Li^+^ ions. These tunneled structures can promote a high degree of structural stability, which is crucial for ensuring long‐term cycling performance and overall durability in LIBs. Additionally, the presence of these tunnels allows for the accommodation and diffusion of lithium ions, which is essential for optimizing the charge storage capability of the material. **Figure** **7**a shows the crystal structure of Li_2_Ti_6_O_13_, in which all Ti atoms are surrounded by six oxygen atoms forming distorted TiO_6_ octahedra.^[^ ^157^ ^]^ Insertion of Li^+^ into Li_2_Ti_6_O_13_ occurs at an average voltage of 1.5 V, which delivers a high discharge capacity of 250 mAh g^−1^.^[^ ^157^ ^]^ Compared with Li_2_Ti_6_O_13_, Na_2_Ti_6_O_13_ exhibits a different crystal structure,^[^ ^158^ ^]^ as shown in Figure 7b. Notably, Na_2_Ti_6_O_13_ undergoes a real topotactical Li^+^ insertion reaction with retention of the skeleton Ti‐O framework structure.^[^ ^88^, ^158^ ^]^ It can deliver a maximum theoretical capacity of 297 mAh g^−1^.^[^ ^156^ ^]^ For K_2_Ti_6_O_13_, it shows almost the same crystal structure as Na_2_Ti_6_O_13_, as illustrated in Figure 7c.^[^ ^159^ ^]^ When used as an anode for LIBs, it can display superior rate capability and excellent cycling stability due to its good electronic conductivity and large open framework.^[^ ^159^ ^]^ To improve the performance of Ti‐based oxides, introducing guest cation ions into the crystal structure is an effective strategy. For example, Liu. et al. developed the proton exchange‐insertion‐exfoliation method to prepare Na_2_Ti_6‐x_Mo_x_O_13_ (NTMO).^[^ ^89^ ^]^ Compared with pristine Na_2_Ti_6_O_13_, the NTMO shows faster Li^+^ diffusion kinetics (Figure 7d). Moreover, the doped NTMO can boost the facile Li^+^ ion transport channels and expose facets with more reversible active sites.^[^ ^89^ ^]^ Overall, the tunnel‐structured Ti‐based oxides represent a significant area of interest in materials research, particularly in energy storage devices. Although considerable efforts have been focused on developing the Ti‐based oxides, the underlying mechanism by which tunnel affects the insertion/extraction of Li^+^ is poorly understood by in situ TEM. Continued studies and developments of these materials hold the potential to further advance the performance and efficiency of LIBs by leveraging the capabilities of in situ TEM techniques.

## 3 In Situ TEM Studies of Tunnel‐Structured Materials for SIBs

Sodium‐ion batteries (SIBs) have become a prominent trend in the progression of secondary batteries due to the natural abundance and cost‐effectiveness of sodium. SIBs share many similarities with LIBs, including the manufacturing process and “rocking‐chair” working principle.^[^ ^160^, ^161^, ^162^, ^163^, ^164^ ^]^ Nevertheless, several scientific challenges still require resolution before the performance of SIBs can rival that of LIBs. In particular, the larger ionic size of Na^+^ relative to Li^+^ results in slow ion diffusion in active materials and low energy efficiencies in batteries.^[^ ^165^, ^166^, ^167^ ^]^ Interestingly, tunnel‐type materials can provide a wide diffusion path and accommodate structural strain during the Na^+^ (de)insertion process, which has been extensively studied for SIBs.^[^ ^168^, ^169^, ^170^ ^]^ Directly observing the evolution of tunnels during sodiation/desodiation processes is crucial for revealing the underlying mechanisms of electrochemical reactions. In this section, we offer a summary of in situ TEM studies on reaction mechanism, phase transformation, structural evolution, and performance degradation of tunnel‐type electrode materials for SIBs.

Tunnel‐type nanomaterials, such as Mn‐based oxides,^[^ ^169^, ^170^, ^171^, ^172^, ^173^, ^174^ ^]^ Ti‐based oxides,^[^ ^175^, ^176^, ^177^, ^178^, ^179^ ^]^V‐based oxides,^[^ ^180^, ^181^, ^182^, ^183^, ^184^ ^]^ and Nb‐based oxides,^[^ ^185^, ^186^, ^187^, ^188^, ^189^ ^]^ etc. have been extensively studied as electrode materials for SIBs because of their unique tunnels for free and fast Na^+^ insertion/extraction. In this section, we will classify and summarize tunnel‐structured nanomaterials based on their roles as cathode and anode materials in SIBs. **Table** **3** comprehensively summarizes the electrochemical performances of cathode materials in SIBs.

**Table 3:** Summary of the electrochemical performances of cathode materials in SIBs.

Electrode material	Electrolyte	Rate performance	Discharge capability	Cycle capability	Ref.
NMO‐2	1M NaClO_4_	76.6% retention (3 A g^−1^)	116.3mAh g^−1^ (10 mA g^−1^)	800 cycles, 82.3% retention	[164]
Tunnel‐type Na_0.44_MnO_2_	1 M NaClO_4_	97.2% retention (0.12 A g^−1^)	115 mA h ^−1^ (7.2 mA g^−1^)	300 cycles, 80% retention	[165]
Na_0.42_Mn_0.96_Ti_0.04_O_2_	1 M NaClO_4_	70.9% retention (2 A g^−1^)	116.4 mAh g^−1^ (20 mA g^−1^)	5000 cycles, 67.0% retention	[167]
NMO‐3M	1 M NaClO_4_	67.% retention (1.2 A g^−1^)	178.9 mAh g^−1^ (12 mA g^−1^)	100 cycles, 77.8% retention	[168]
Na_0.44_MnO_2_	1 M Na_2_SO_4_	41.2% retention (0.605 A g^−1^)	52 mAh ^−1^ (121 mA g^−1^)	300 cycles, 65.4% retention	[171]
NMO‐TM	1 M NaClO_4_	91.3% retention (0.242 A g^−1^)	110 mAh g^−1^ (121 mA g^−1^)	200 cycles, 93. 6% retention	[172]
NMOL0.06	1 M NaClO_4_	77.3% retention (0.6 A g^−1^)	110.7mAh g^−1^ (240 mA g^−1^)	400 cycles, 90.2% retention	[173]
L/T‐NaMT‐1	1 M NaClO_4_	67.2% retention (0.24 A g^−1^)	152.0mAh g^−1^ (240 mA g^−1^)	300 cycles, 71.0% retention	[174]
Na_0.282_V_2_O_5_	1 M NaPF_6_	56.9% retention (1 A g^−1^)	240 mAh g^−1^ (50 mA g^−1^)	400 cycles. 83% retention	[180]
V_1.5_Cr_0.5_O_4.5_H/CNT	1 M NaPF_6_	70.2% retention (0.9 A g^−1^)	306 mAh g^−1^ (15 mA g^−1^)	100 cycles. 77.1% retention	[181]
V_2_O_5_/Ca_0.17_V_2_O_5_ film	1 M NaClO_4_/PC	79.9% retention (0.7 A g^−1^)	153.5 mAh g^−1^ (99 mA g^−1^)	100 cycles. 104% retention	[183]
NaV_6_O_15_	1 M NaClO_4_	56.9% retention (10 A g^−1^)	217.2 mA h g^−1^ (100 mA g^−1^)	500 cycles. 96% retention	[184]
V_2_O_5_‐Vo‐A	1 M NaPF_6_	64% retention (1 A g^−1^)	325 mAh g^−1^ (50 mA g^−1^)	360 cycles. 94.5% retention	[190]
β‐NaVOPO_4_	1 M NaClO_4_	_	316 mAh g^−1^ (17 mA g^−1^)	50 cycles. 19% retention	[191]
H‐PB	1 M NaClO_4_	70.3% retention (12 A g^−1^)	60 mAh g^−1^ (240 mA g^−1^)	2000 cycles. 62% retention	[192]
MNHCF‐3	1 M NaClO_4_	78.4% retention (3 A g^−1^)	136.5 mA h g^−1^ (15 mA g^−1^)	1700 cycles. 82.6% retention	[193]
HE‐PBA	1.7 M NaClO_4_	39.0% retention (5 A g^−1^)	118.6 mA h g^−1^ (100 mA g^−1^)	1800 cycles. 81.2% retention	[194]
FeMnCu	1 M NaClO_4_	66.48% retention (0.5 A g^−1^)	127 mAh g^−1^ (30 mA g^−1^)	500 cycles. 60.6% retention	[195]
PB‐130	1 M NaClO_4_	75.7% retention (2 A g^−1^)	113.6 mAh g^−1^ (30 mA g^−1^)	1200 cycles. 85.5% retention	[196]
HEPBA‐Etched‐10	1 M NaClO_4_	60.4% retention (4 A g^−1^)	126.5mAh g^−1^ (100 mA g^−1^)	1000 cycles. 75.6% retention	[197]
FeFe(CN)_6_	1 M NaPF_6_	84% retention (2.4 A g^−1^)	109mAh g^−1^ (60 mA g^−1^)	150 cycles. 95.8% retention	[198]
LQ‐NaFe	1 M NaPF_6_	_	140 mA h g^−1^ (25 mA g^−1^)	70 cycles. 36.5% retention	[199]

### 3.1 Cathode Materials

Tunnel‐structured cathode materials play a pivotal role in advancing SIBs by enabling rapid Na^+^ insertion/extraction and ensuring structural stability throughout cycling. In the following sections, we delve into the exploration of key tunnel‐structured cathode materials through in situ TEM techniques, shedding light on their structural evolution, electrochemical behavior, and performance characteristics.

#### 3.1.1 Mn‐Based Oxides

**Figure 8:**
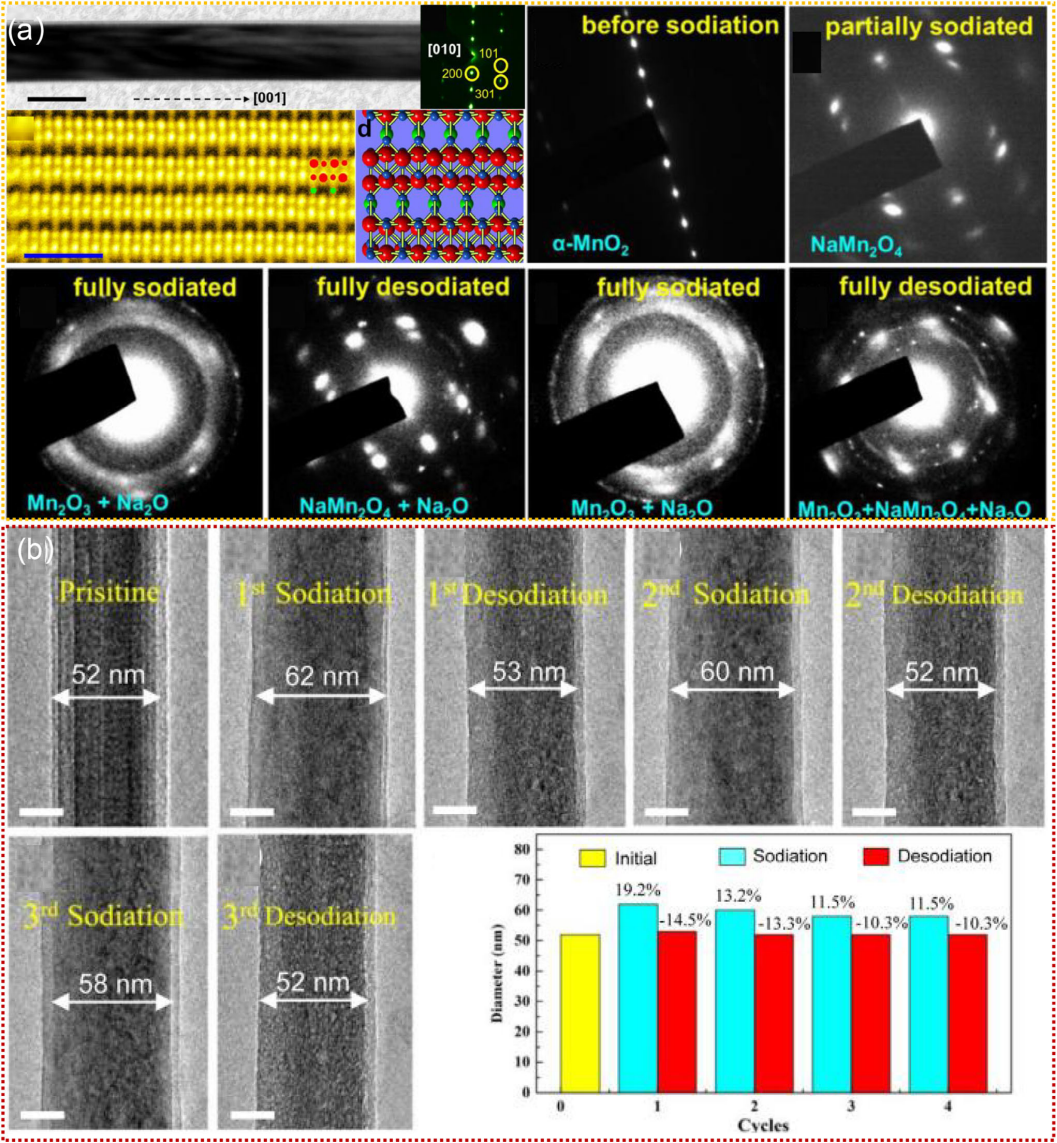
a) TEM images of one α‐MnO_2_ nanowire with the atomic model, and the corresponding SAED patterns of the α‐MnO_2_ nanowire at various (de)sodiation states. Reproduced with permission.^[^ ^166^ ^]^ Copyright 2016, Elsevier Ltd. b) Microstructure evolutions of τ‐MnO_2_ nanowires during the (de)sodiation processes. Reproduced with permission.^[^ ^169^ ^]^ Copyright 2021, Elsevier Ltd.

Mn‐based oxides with tunnel structures have garnered significant attention for their application as cathode materials in SIBs. Among them, Na_x_MnO_2_ nanomaterials are well known for the different polymorphs possessing various open crystallographic frameworks to facilitate the initial Na^+^ insertion, whose initial discharge capacity can reach up to 350 mAh g^−1^.^[^ ^168^, ^169^, ^170^, ^171^ ^]^ Nevertheless, their Na storage mechanisms remain elusive because of the complicated tunnel structures, and the transport kinetics of Na^+^ in such tunnels and concomitant tunnel structure evolution are still poorly understood. To reveal the tunnel structure evolution and Na^+^ storage mechanism of Na_x_MnO_2_ nanomaterials, considerable studies have utilized in situ TEM to track their dynamic morphological and structural evolution during the (de)sodiation processes. For example, Yuan. et al. revealed the morphology and phase evolution of α‐MnO_2_ nanowire during(de)sodiation by using in situ TEM, as shown in **Figure** **8**a.^[^ ^166^ ^]^ The first sodiation process started with tunnel‐based Na^+^ intercalation and then experienced the intermediate phase formation of Na_0.5_MnO_2_ as a result of tunnel degradation and ended with the Mn_2_O_3_ phase.^[^ ^166^ ^]^ The inserted Na^+^ could be partially extracted, and the subsequent cycles were dominated by the reversible conversion reaction between Na_0.5_MnO_2_ and Mn_2_O_3_.^[^ ^166^ ^]^ Cai. et al. utilized in situ TEM to visualize anisotropic sodiation degrees and Na^+^ storage mechanisms of todorokite‐type MnO_2_ (τ‐MnO_2_), as illustrated in Figure 8b.^[^ ^169^ ^]^ These studies provide valuable insights into Na^+^ storage mechanisms of tunnel‐type Na_x_MnO_2_ and help to further improve the electrochemical performance of these materials by proper structure engineering and chemical modification. Notably, cation substitution is considered an efficient approach to adjust the crystal structure and enhance the electrochemical properties of manganese oxides. For instance, the Ti substitution for Mn sites could induce a contraction of the TMO_6_ octahedron, enlarge the spacing of the Na layer in P2‐Na_2/3_MnO_2,_ and inhibit electron delocalization, which significantly enhances the sodium storage capacity.^[^ ^162^, ^174^ ^]^ Chen. et al. reported that 5% Ni doping could improve the reversible capacity, rate performance, cycling life, and reaction kinetics of Na_0.6_Mn_1‐x_Ni_x_O_2_.^[^ ^185^ ^]^ Overall, tunnel‐structured Mn‐based oxides exhibit promising potential for applications in SIBs, which also present a compelling avenue for further development in energy storage systems.

#### 3.1.2 V‐Based Oxides

**Figure 9:**
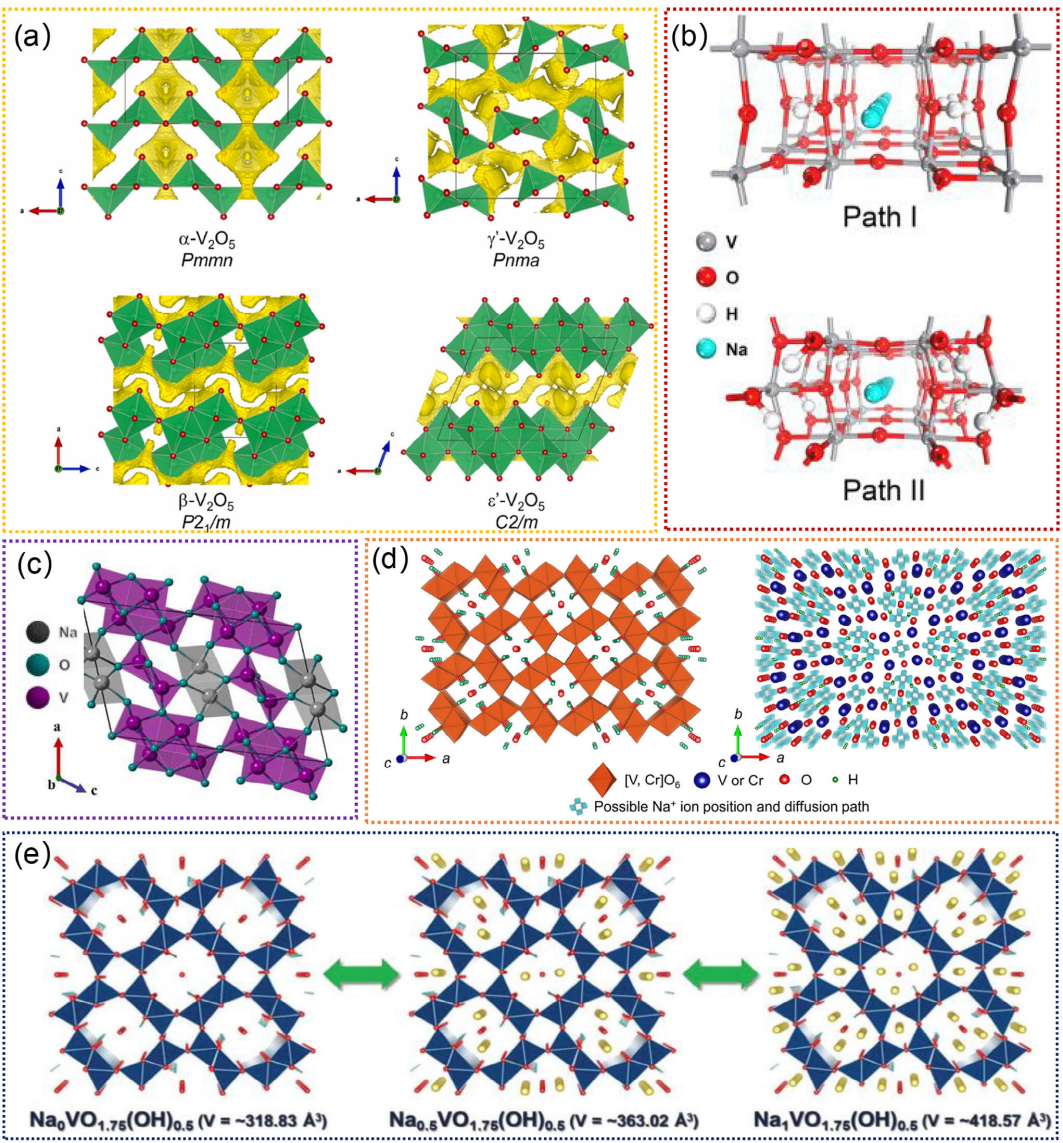
a) The simulated crystal structures of different V_2_O_5_ forms. VO_5_ square‐based pyramids and VO_6_ octahedra (green), and oxygen atoms (red spheres). Reproduced with permission.^[^ ^205^ ^]^ Copyright 2024, Elsevier Ltd. b) Na‐ion diffusion pathway I along the [010] and pathway II along the [001] directions of H_2_V_2_O_5_. Reproduced with permission.^[^ ^200^ ^]^ Copyright 2017, American Chemical Society. c) Crystal structure of β‐NaV_6_O_15_ along the b‐axis. Reproduced with permission.^[^ ^184^ ^]^ Copyright 2021, American Chemical Society. d) Crystal structure of V_1.5_Cr_0.5_O_4.5_H, and possible Na^+^ positions and diffusion paths by 3D bond‐valence‐energy landscape (BVEL) analysis. Reproduced with permission.^[^ ^181^ ^]^ Copyright 2020, Elsevier Ltd. e) Structural change of Na_x_VO_1.75_(OH)_0.5_ (0 ≤ *x* ≤ 1) predicted using first‐principles calculations. Reproduced with permission.^[^ ^21^ ^]^ Copyright 2019, Wiley‐VCH GmbH.

Vanadium oxides have emerged as attractive cathode materials for SIBs due to their favorable electrochemical properties, including their high capacity, good cycling stability, and relatively low cost.^[^ ^200^, ^201^, ^202^, ^203^, ^204^, ^205^ ^]^ Additionally, the ability to accommodate the (de)intercalation of Na^+^ within their structure further enhances their appeal as electrode materials for SIBs. For example, layer‐structural V_2_O_5_ exhibits various polymorphs, including α‐V_2_O_5_, β‐V_2_O_5_, γ‐V_2_O_5_, ε‐V_2_O_5_, as depicted in **Figure** **9**a.^[^ ^205^ ^]^ Owing to the high oxidation state of vanadium and a theoretical capacity of 443 mAh g^−1^, these materials are highly attractive as electrode materials for SIBs.^[^ ^184^, ^228^ ^]^ Nevertheless, the low electronic conduction and slow diffusion of Na^+^ ions lead to inadequate rate performance and cycling stability.^[^ ^200^, ^205^ ^]^ Incorporating hydrogen provides an alternative method to modify the atomic and electronic structures of vanadium oxides. Shi et al. demonstrated an H^+^‐incorporated α‐V_2_O_5_ (H_2_V_2_O_5_), which shows enlarged diffusion channels along the [001] and [010] directions (Figure 9b).^[^ ^200^ ^]^ Moreover, the atomic structure of H_2_V_2_O_5_ presents the most favorable conditions for rapid Na^+^ transport.^[^ ^200^ ^]^ To further improve the performance, pre‐intercalation of cations (e.g., Na^+^, K^+^, etc.) is the most effective and facile strategy to stabilize the 2D layer structure, and the intercalation of cations between the layers creates a 3D tunnel structure that acts as “pillars” to stabilize the crystal structure during the charging/discharging process.^[^ ^180^, ^184^ ^]^ Osman et al. proposed a 3D pillaring tunnel structure of NaV_6_O_15_ (NVO) nanorods (Figure 9c), which demonstrates rapid ion and electron transport, resulting in exceptional rate performance and excellent cycling stability.^[^ ^184^ ^]^ KO et al. reported a tunnel‐type V_1.5_Cr_0.5_O_4.5_H with a tetragonal crystal system and I 4/m space group.^[^ ^181^ ^]^ It consists of infinite chains of VO_6_ and CrO_6_ octahedra sharing their edge or point with each other (Figure 9d), resulting in large vacant sites in the crystal structure, such as [2 × 2] and [1 × 1] tunnels.^[^ ^181^ ^]^ They revealed that 3 mol of Na^+^ ions per 2 mol of transition metal ions, such as V and Cr, can be intercalated into the crystal structure of V_1.5_Cr_0.5_O_4.5_H nanorods, demonstrating the outstanding Na^+^ storage capacity. Moreover, the hollandite‐type VO_1.75_(OH)_0.5_ with large tunnels enables accommodation of the large Na^+^ ions, exhibiting fast Na^+^ ion diffusion capability. Interestingly, Na^+^ pre‐intercalated VO_1.75_(OH)_0.5_ can lead to the formation of a stable intermediate phase during the Na^+^ (de)intercalation process, and the formed Na_x_VO_1.75_(OH)_0.5_ can undergo a single‐phase reaction with a sloppy charging/discharging curve.^[^ ^21^ ^]^ As shown in Figure 9e, the Na_x_VO_1.75_(OH)_0.5_ exhibits large tunnels for Na^+^ accommodation and diffusion, which can effectively stabilize the tunnel structure of VO_1.75_(OH)_0.5_.^[^ ^21^ ^]^ The open structure of hollandite‐type Na_x_VO_1.75_(OH)_0.5_ contributes to its excellent rate capability and cycling stability. Overall, tunnel‐type vanadium oxides have garnered significant interest as potential electrode materials for SIBs. These materials possess a unique tunnel structure that allows for the reversible intercalation and deintercalation of Na^+^, enabling high capacity and excellent cycling stability. However, understanding and addressing challenges related to complex phase transitions of the tunnel structure is crucial. Further optimization efforts should focus on elucidating these complexities for enhanced performance.

#### 3.1.3 Prussian Blue Analogues

**Figure 10:**
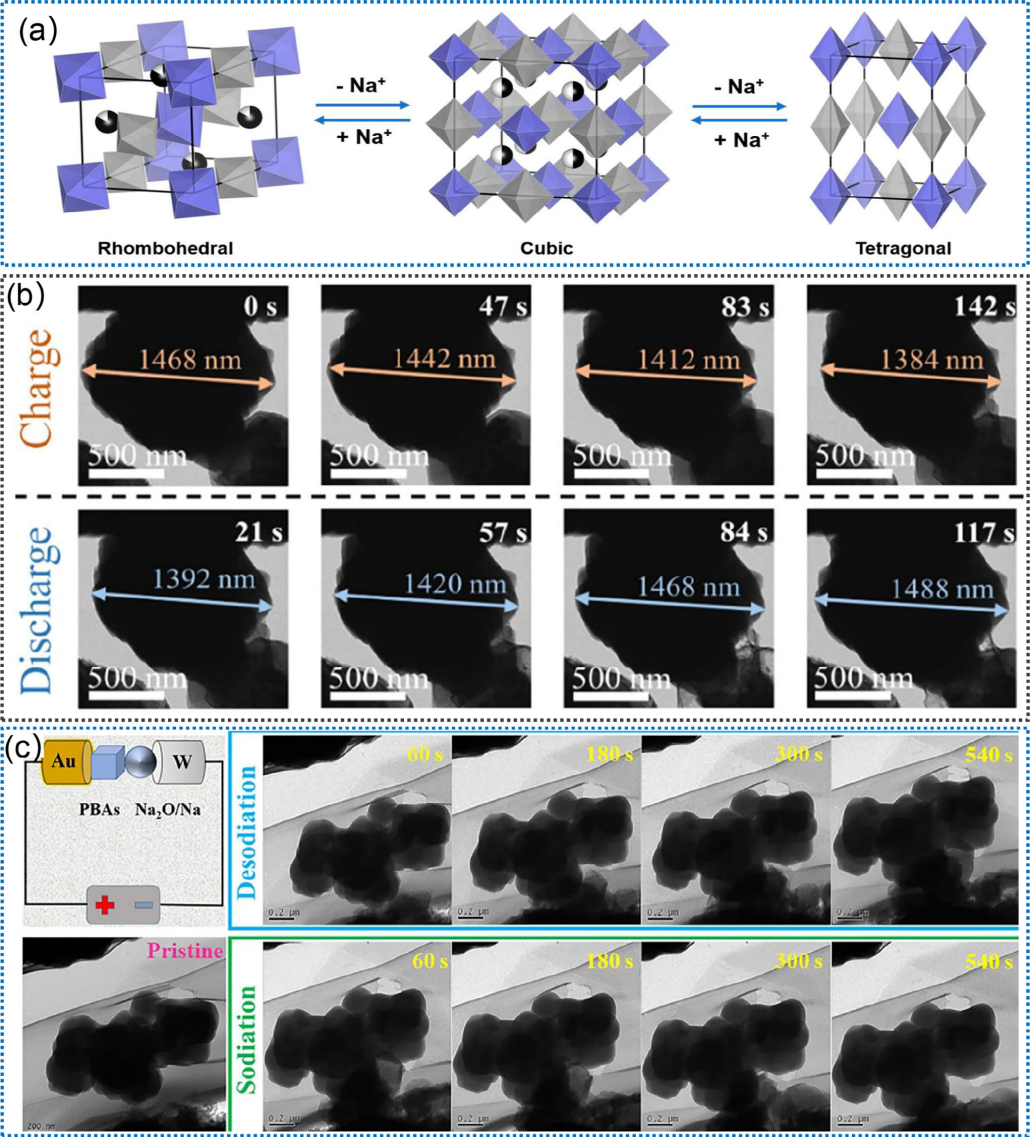
a) The crystal structural changes of Na_2‐x_Fe[Fe(CN)_6_]_y_ during Na^+^ (de)insertion. Reproduced with permission.^[^ ^206^ ^]^ Copyright 2022, American Chemical Society. b) In situ TEM observations of high‐quality Prussian blue during (de)sodiation processes. Reproduced with permission.^[^ ^207^ ^]^ Copyright 2022, American Chemical Society. c) Schematic illustration of in situ TEM setup, and in situ TEM measurements of Mn‐based Prussian blue analogues during (de)sodiation processes. Reproduced with permission.^[^ ^208^ ^]^ Copyright 2024, American Chemical Society.

PBAs have been extensively studied as promising cathode materials for SIBs due to their 3D open frameworks and large interstitial sites, which allow reversible Na^+^ storage and fast ion transportation.^[^ ^206^, ^214^ ^]^ For example, Qin et al. proposed a highly crystallized Prussian blue (Na_2_Fe_4_[Fe(CN)_6_]_3_), which exhibits efficient electron transfer and smooth ion diffusion, resulting in excellent rate performance and long‐term cycling stability in Na^+^ ion storage.^[^ ^214^ ^]^ Wang et al. reported a highly crystallized Na_2‐x_Fe[Fe(CN)_6_]_y_, which displays a highly reversible Na^+^ ion storage, as illustrated in **Figure** **10**a.^[^ ^206^ ^]^ By suppressing the structure defects, the material demonstrates sufficient Na^+^ storage sites and fast cation migration channels.^[^ ^206^ ^]^ This underscores the importance of structural integrity in facilitating efficient Na^+^ ion transport and storage, thus showcasing the material's potential for high‐performance energy storage applications. Nevertheless, PBAs usually suffer from low capacity utilization due to the high ratio of vacancy defects in Fe(CN)_6_.^[^ ^207^, ^208^ ^]^ These vacancy defects can significantly impact the material's ability to effectively store and release ions, thereby limiting its overall capacity utilization. To overcome this limitation, Ran et al. proposed a high‐entropy strategy to enhance both the specific capacity and capacity retention by introducing equimolar Co, Fe, Cu, and Mn at the Ni sites in PBA frameworks.^[^ ^209^ ^]^ The introduced four transition metals significantly increase the electrochemically active sites, and more stable hosts for Na^+^ ion (de)intercalation^[^ ^209^ ^]^ Li et al. reported a beneficial and applicable strategy to introduce Fe into PBA materials for SIBs, which displays low vacancies and good cycling stability after 300 cycles.^[^ ^208^ ^]^ Moreover, they demonstrated that high‐quality Prussian blue allows fast Na^+^ ion mobility and a high degree of reversibility during the charging/discharging process by in situ TEM (Figure 10b). This finding underscores the importance of material quality in facilitating efficient ion transport and reversible electrochemical reactions, thereby highlighting the potential of high‐quality PBAs for advanced SIB applications. Recently, Peng et al. presented Na‐rich Mn‐based PBAs that exhibit exceptional rate capability and remarkably long‐term cycling stability (90.1% of its capacity after 10,000 cycles).^[^ ^208^ ^]^ In situ TEM revealed that the exceptional performance of this electrode stems from its highly reversible three‐phase transformations and the synergistic effects of the tri‐metal (Mn‐Ni‐Fe) composition (Figure 10c). These findings highlight the importance of understanding the intricate structural and compositional dynamics of the PBA materials during the charging/discharging processes. By leveraging reversible phase transformations and the synergistic effects of multiple metals, these sodium‐rich, Mn‐based Prussian blue analogues demonstrate significant promise for robust and efficient energy storage applications, offering insights for the development of advanced SIB technologies.

### 3.2 Anode Materials

Tunnel‐structured anode materials play a crucial role in SIBs by providing a framework to accommodate the large volumes of Na^+^ ions during charge and discharge cycles while preserving structural stability. Below, we will discuss key anode materials studied in SIBs by using in situ TEM techniques. **Table** **4** comprehensively summarizes the electrochemical performances of anode materials in SIBs.

**Table 4:** Summary of the electrochemical performances of anode materials in SIBs.

Electrode material	Electrolyte	Rate performance	Discharge capability	Cycle capability	Refs.
KTO@NCNFs	1 M NaPF_6_	37.6% retention (1 A g^−1^)	167.0mAh g^−1^ (20 mA g^−1^)	5000 cycles. 302% retention	[175]
NNTO	1 M NaClO_4_	9.2% retention (2 A g^−1^)	212.5mAh g^−1^ (20 mA g^−1^)	4000 cycles. 71.8% retention	[177]
K_2_Ti_6_O_13_ nanowire	1 M NaPF_6_	33% retention (1 A g^−1^)	186 mAh g^−1^ (20 mA g^−1^)	100 cycles. 83% retention	[178]
Na_2_Ti_6_O_13_ nanorods	1 M NaPF_6_	74% retention (1 A g^−1^)	172 mAh g^−1^ (100 mA g^−1^)	800 cycles. 97% retention	[179]
K_0.2_TiO_2_	1 M NaClO_4_	52% retention (0.671 A g^−1^)	105 mAh g^−1^ (42 mA g^−1^)	600 cycles. 94.5% retention	[215]
Mo_0.1_TiO_2_‐x@C	1 M NaPF_6_	56.2% retention (2 A g^−1^)	501mAh g^−1^ (500 mA g^−1^)	3000 cycles. 122% retention	[216]
CoSe_2_@Ti_3_C_2_Tx	1 M NaSO_3_CF_3_	34.4% retention (20 A g^−1^)	820.1mAhg^−1^ (100 mA g^−1^)	1300 cycles. 100% retention	[217]
TiO_2_@CNT@C el	1 M NaClO_4_	50.2% retention (4 A g^−1^)	277mAh g^−1^ (50 mA g^−1^)	1000 cycles. 93% retention	[218]
S‐TiO_2_/C	1 M NaClO_4_	42.2% retention (15 A g^−1^)	149mAh g^−1^ (5000 mA g^−1^)	500 cycles. 100% retention	[219]
Nb_2_O_5_/rGO‐H	1 M NaPF_6_	32.5% retention (10 A g^−1^)	422mAh g^−1^ (50 mA g^−1^)	2000 cycles. 91% retention	[190]
NTO/CT	1 M NaClO_4_	41.8% retention (3 A g^−1^)	350mAh g^−1^ (100 mA g^−1^)	1000 cycles. 30.5% retention	[220]
Nb_2_O_5_@C/rGO‐50 d	1 M NaPF_6_	38.6% retention (3 A g^−1^)	285mAh g^−1^ (25 mA g^−1^)	_	[221]
NF@C‐650	1 M NaPF_6_	49.8% retention (4 A g^−1^)	245mAh g^−1^ (50 mA g^−1^)	1000 cycles. 100% retention	[192]
Nb_2_O_5_@3D PRS	1 M NaClO_4_	35.8% retention (2.5 A g^−1^)	716mAh g^−1^ (50 mA g^−1^)	7500 cycles. 100% retention	[222]
G‐Nb_2_O_5_ nanosheets	1 M NaPF_6_	_	230mAh g^−1^ (50 mA g^−1^)	1000 cycles. 77.6% retention	[223]
G@mNb_2_O_5_	1 M NaPF_6_	62.5% retention (0.5 A g^−1^)	293mAh g^−1^ (50 mA g^−1^)	2000 cycles. 100% retention	[193]
Nb_2_O_5_‐x@MEC	1 M NaPF_6_	28.9% retention (20 A g^−1^)	450mAh g^−1^ (200 mA g^−1^)	1000 cycles. 96.8% retention	[194]
Nb‐O/N@C	1 M NaClO_4_	42.7% retention (10 A g^−1^)	959.1mAh g^−1^ (100 mA g^−1^)	8000 cycles. 124% retention	[195]
Bi_0.67_NbS_2_	1 M NaPF_6_	75.7% retention (36.1 A g^−1^)	325mAh g^−1^ (361 mA g^−1^)	5000 cycles. 103% retention	[196]
T‐Nb_2_O_5_‐C‐rGO	1 M NaPF_6_	82% retention (10 A g^−1^)	240mAh g^−1^ (100 mA g^−1^)	1000 cycles. 68% retention	[224]
Ti2C/NTO	1 M NaPF_6_	74.% retention (5 A g^−1^)	409mAh g^−1^ (100 mA g^−1^)	4500 cycles. 74% retention	[197]
m‐Nb_2_O_5_/CNF	1 M NaPF_6_	62.7% retention (20 A g^−1^)	1053mAh g^−1^ (100 mA g^−1^)	500 cycles. 92% retention	[191]
Nb_2_O_5_@WS_2_ CNFs	1 M NaClO_4_	17.3% retention (10 A g^−1^)	305mAh g^−1^ (200 mA g^−1^)	200 cycles. 62% retention	[198]
α‐Nb_2_O_5_@C@Ti_3_C_2_	1 M NaClO_4_	28.9% retention (20 A g^−1^)	689.2mAh g^−1^ (20 mA g^−1^)	3000 cycles. 99.1% retention	[199]
Ti_2_Nb_2_O_9_	1 M NaPF_6_	_	244mAh g^−1^ (100 mA g^−1^)	2000 cycles. 75% retention	[205]
hollandite K_x_TiO_2_	1 M NaPF_6_	51% retention (1 A g^−1^)	131 mAh g^−1^ (20 mA g^−1^)	1000 cycles. 100% retention	[225]

#### 3.2.1 Ti‐Based Oxides

**Figure 11:**
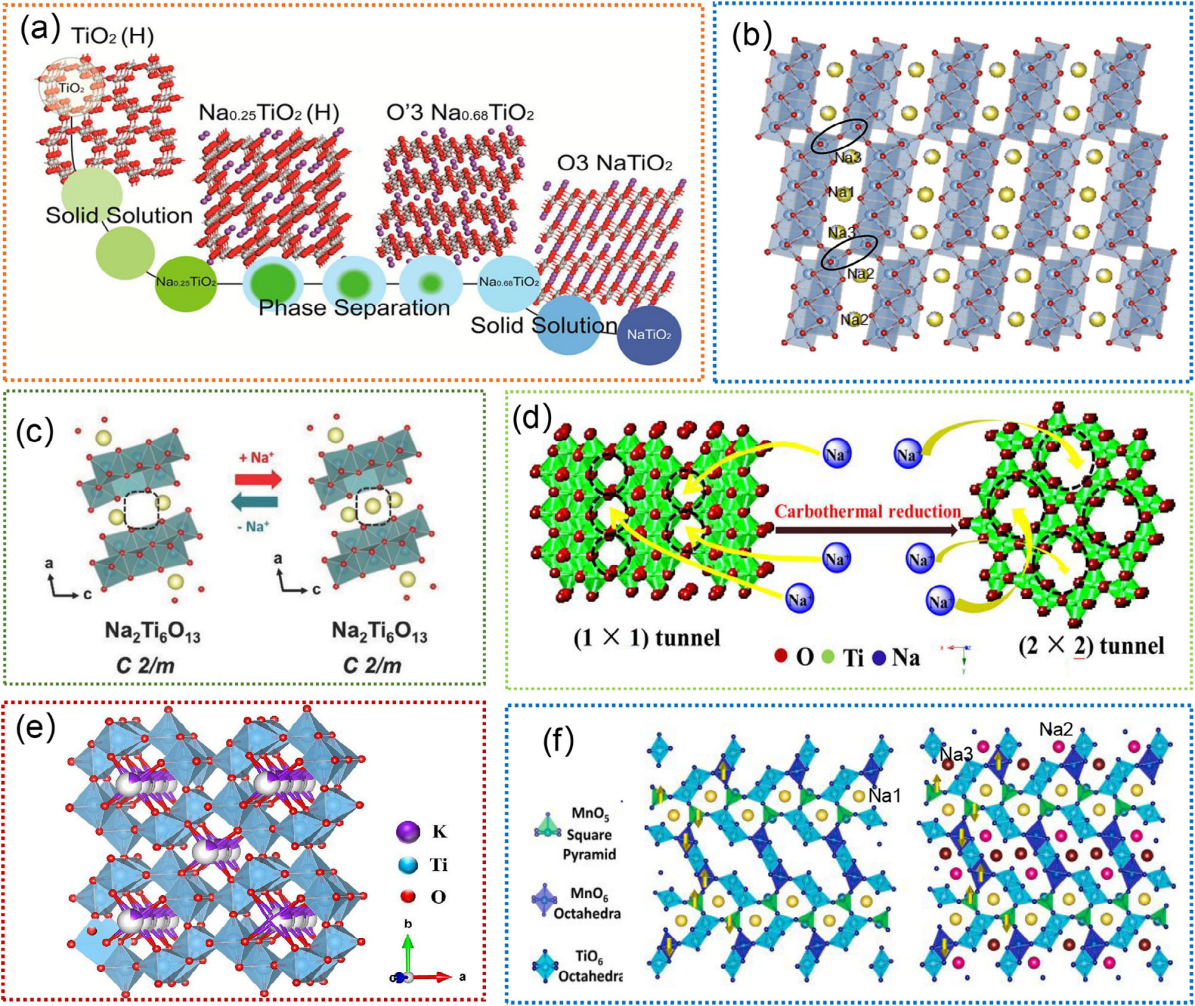
a) The simulated crystal structure models of hollanditeTiO_2_ and Na_x_TiO_2_. Reproduced with permission.^[^ ^227^ ^]^ Copyright 2017, American Chemical Society. b) Schematic crystal structure of Na_2_Ti_7_O_15_. Reproduced with permission.^[^ ^226^ ^]^ Copyright 2016, Elsevier Ltd. c) Schematic diagram of structure evolution of Na_2_Ti_6_O_13_ during the Na^+^ insertion/extraction process. Reproduced with permission.^[^ ^177^ ^]^ Copyright 2018, Wiley‐VCH GmbH. d) Schematic structure illustration of K_2_Ti_6_O_13_ precursor (left) and the hollandite K_x_TiO_2_ product. Reproduced with permission.^[^ ^225^ ^]^ Copyright 2017, American Chemical Society. e) The crystal structure of hollandite‐type K_1.28_Ti_8_O_16_. Reproduced with permission.^[^ ^175^ ^]^ Copyright 2022, Elsevier Ltd. f) The simulated crystal structure models of (de)intercalated Na_x_Mn_4_Ti_5_O_18_. Reproduced with permission.^[^ ^176^ ^]^ Copyright 2024, Elsevier Ltd.

Ti‐based oxides, particularly those with a tunnel structure, have shown exceptional sodium ion storage capacity. Hollandite‐type TiO_2_ nanomaterials are one of the most attractive anodes for SIBs due to their larger tunnels for fast insertion and diffusion of Na^+^.^[^ ^200^, ^201^, ^202^, ^203^, ^204^, ^205^, ^206^, ^207^, ^214^, ^215^, ^225^, ^226^, ^227^, ^228^ ^]^ The c‐axis of TiO_2_ is recognized as a potential pathway for Na^+^ diffusion and insertion.^[^ ^227^ ^]^ Notably, a monoclinic Na_0.25_TiO_2_ (I2/m) can be formed upon the initial insertion of Na^+^, and then a layered O3‐NaTiO_2_ phase is generated after a high concentration of Na^+^ insertion, as illustrated in **Figure** **11**a.^[^ ^227^ ^]^ Such continuous phase transitions can easily cause the disappearance of the TiO_2_ tunnels, resulting in a rapid decline in sodium storage capacity. To optimize its sodium storage performance, pre‐insertion of Na^+^/K^+^ into the TiO_2_ crystal is considered an effective strategy. For example, Tao. et al. demonstrated Na_2_Ti_7_O_15_ (Figure 11b) can be sodiated to Na_3.5_Ti_7_O_15_ with minimal lattice expansion upon Na^+^ intercalation.^[^ ^226^ ^]^ Additionally, the insertion of Na^+^ can enhance its conductivity, thus improving its sodium storage properties.^[^ ^226^ ^]^ Wu. et al. revealed that tunnel Na_2_Ti_6_O_13_ crystal structure exhibits no significant structural variation during Na^+^ insertion/extraction.^[^ ^177^ ^]^ During Na^+^ insertion, Na^+^ initially occupies 2d sites, then progresses to occupy 4i sites and eventually reaches 2c positions, as illustrated in Figure 11c.^[^ ^177^ ^]^ Besides, they demonstrated the high ionic conductivity and superior cycling stability of the tunnel Na_2_Ti_6_O_13_ anodes. Zhang. et al. reported that the hollandite K_x_TiO_2_ with large (2 × 2) tunnels could accommodate more Na^+^ and facilitate the Na^+^ diffusion in the crystal structure, as illustrated in Figure 11d.^[^ ^225^ ^]^ Owing to the open tunnel, the K_x_TiO_2_ hollandite can deliver a stable reversible capacity of 131 mAh g^−1^ and superior rate capability.^[^ ^225^ ^]^ Recently, Chen. et al. demonstrated a novel 2 × 2 tunnel‐structured K_1.28_Ti_8_O_16_ (Figure 11e), which could contain more Na^+^, promote the Na^+^ diffusion in the tunnels, and maintain structural stability.^[^ ^175^ ^]^ Another typical method is recombination with multivalent ions, which could effectively enhance electronic conductivity and reduce structural damage during Na^+^ insertion/extraction. For instance, tunnel‐structured Na_x_Mn_y_Ti_z_O_2_ materials are considered promising cathodes for SIBs due to their favorable tunnels and robust structural framework.^[^ ^172^, ^176^ ^]^ Mandal. et al. found that Z‐shaped tunnel and polyhedral pentagonal void of Na_4_Mn_4_Ti_5_O_18_ could act as guest sites for reversible Na^+^ migration, as shown in Figure 11f.^[^ ^176^ ^]^ Such compounds present two redox centers of Mn^3+^/Mn^4+^ and Ti^3+^/Ti^4+^, which can greatly enhance the sodium storage capacity. Despite significant efforts that have been dedicated to the studies on tunnel‐structured Ti‐based oxides, the complex phase transitions of tunnel structure and sodium/vacancy ordering are still not clearly elucidated. By combining in situ TEM, a more comprehensive understanding of the behaviors of tunnel‐structured Ti‐based oxides in SIBs is urgent and necessary.

#### 3.2.2 Nb‐Based Oxides

**Figure 12:**
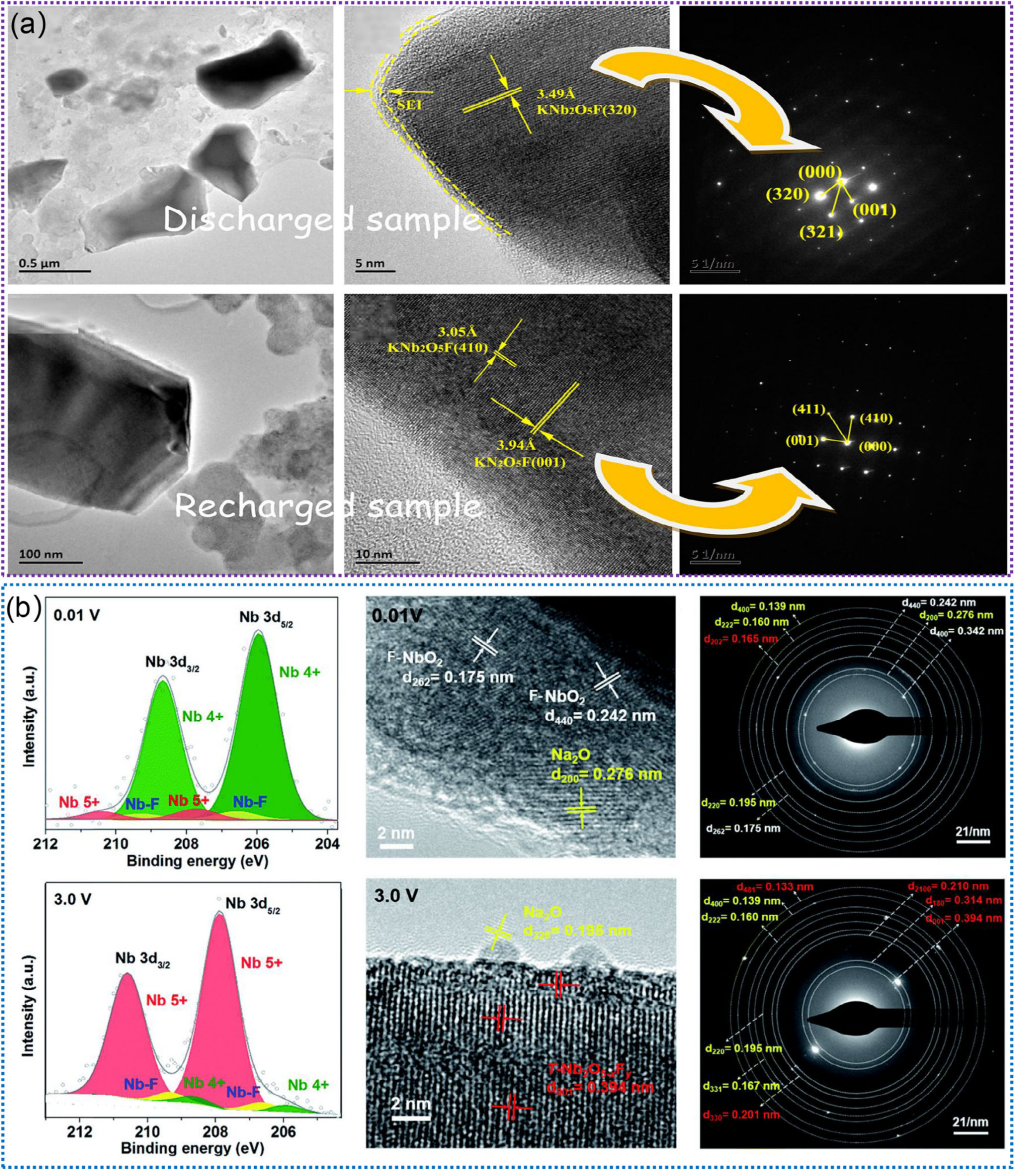
a) TEM, HRTEM images and SAED pattern of KNb_2_O_5_F sample after sodiation and desodiation process, respectively. Reproduced with permission.^[^ ^187^ ^]^ Copyright 2016, American Chemical Society. b) The XPS and HRTEM images of the T‐Nb_2_O_5‐x_F_y_/C‐NBs electrode after sodiation (0.01 V) and desodiation (3.0 V). Reproduced with permission.^[^ ^189^ ^]^ Copyright 2019, The Royal Society of Chemistry.

Nb‐based oxides typically demonstrate a conventional sodium intercalation storage mechanism, making them well‐suited as anode materials for SIBs. As a prototypical pseudo‐capacitive material, Nb_2_O_5_ has demonstrated a remarkable high‐rate charge storage capability in Na^+^ ion storage.^[^ ^185^, ^186^, ^187^ ^]^ Li et al. developed an amorphous hydrogenated Nb_2_O_5_ nanomateria, which exhibits rapid and sustainable Na^+^ ion storage.^[^ ^185^ ^]^ Notably, expanding lattice spacing, enhancing conductivity, and reinforcing capacitive reactions at the interface are crucial for achieving superior Na^+^ ion storage performance. For example, Liu et al. employed a template‐directing method to uniformly encapsulate S‐doped T‐Nb_2_O_5_ hollow nanospheres within an S‐doped graphene network, forming S‐Nb_2_O_5_.^[^ ^186^ ^]^ This 3D porous structure not only facilitated efficient electron transmission pathways but also provided an excellent ionic conductive channel, thereby enhancing Na^+^ ion storage performance. The S‐Nb_2_O_5_ anode demonstrated a reversible capacity of 215 mAh g^−1^ at 0.5 C over more than 100 cycles, while maintaining a stable capacity of 100 mAh g^−1^ at 20 C after 3000 cycles.^[^ ^186^ ^]^ Han et al. proposed a novel bronze phase of KNb_2_O_5_F with a tunnel‐type open framework by doping equimolar KF into T‐Nb_2_O_5_, where K and F serve as channel supporters and ligand substitutes. TEM observations (**Figure** **12**a) revealed its minimal cell volume change and high reversibility, which can be attributed to its robust open framework.^[^ ^187^ ^]^ Moreover, the low conductivity of Nb‐based materials can be improved by constructing the niobium oxide/carbon composites, leading to improved electrochemical performance. Wang et al. developed ultrafine niobium oxide nanocrystalline/reduced graphene oxide (Nb_2_O_5_ NCs/rGO) composite via a hydrolysis route, which not only enhances charge transfer but also mitigates the volume changes during cycles, leading to superior rate and cycle performance.^[^ ^188^ ^]^ Wu et al. proposed an approach to enhance the electrochemical properties of T‐Nb_2_O_5_ through fluorine substitution and carbon modification strategies.^[^ ^189^ ^]^ The sodium‐driven compositional and structural changes of the electrodes during sodiation/desodiation were studied by XPS and HRTEM analysis (Figure 12b). These characterization results confirmed that the obtained orthorhombic niobium oxyfluoride/carbon nanobelt composite (T‐Nb_2_O_5‐x_F_y_/C‐NBs) possesses a hierarchical nanoarchitecture with T‐Nb_2_O_5‐x_F_y_ nanoslabs uniformly embedded in a carbon nanobelt matrix to form arrays. This configuration facilitates excellent electron conductivity, electron/ion transport, and structural stability over cycling. Despite considerable research efforts dedicated to tunnel‐structured Nb‐based oxides, the intricate phase transitions of the tunnel structure and sodium/vacancy ordering remain inadequately understood. By combining in situ TEM, a more comprehensive understanding of the behaviors of tunnel‐structured Nb‐based oxides in SIBs is urgent and necessary.

## 4 In Situ TEM Studies of Tunnel‐Structured Materials for PIBs

Potassium ion batteries (PIBs) have garnered significant interest within the scientific community because of their distinctive properties and potential as future battery systems. Compared to LIBs and SIBs, PIBs present an attractive alternative due to the abundance of potassium resources and the lower redox potential of K^+^/K compared to that of Na^+^/Na (−2.93 V vs −2.71 V).^[^ ^229^, ^230^, ^231^, ^232^ ^]^ However, a significant challenge in PIBs lies in identifying suitable electrode materials, especially anode materials, capable of accommodating the huge volume expansion caused by K^+^ ions insertion. So far, several electrode materials for PIBs have been proposed, including layered 2D materials and tunnel‐structured transition metal compounds.^[^ ^233^, ^234^, ^235^ ^]^ Notably, tunnel‐type nanomaterials have been identified as highly promising candidates for PIBs owing to their capacity to offer a broad diffusion path and accommodate structural strain during the K^+^ (de)insertion process.^[^ ^236^, ^237^, ^238^, ^239^, ^240^, ^241^, ^242^ ^]^ Optimizing the design and synthesis of such materials could lead to significant advancements in PIB technology, potentially enhancing the overall performance and longevity of PIBs. It's true that while some aspects of the PIB technology have been partially addressed, such as the battery discharge, the charge storage mechanisms of the electrode are still relatively unknown. Given that PIB research is in its early stages, understanding the fundamental charge storage mechanisms is crucial for the development of effective electrode materials and overall battery optimization. Further characterization (*e.g*., in situ TEM, XRD, and Raman, etc.) of the charge storage mechanisms will be essential for advancing PIB technology and unlocking its full potential. In this section, we provide a summary of in situ TEM studies focusing on tunnel‐type nanomaterials based on their roles as cathode and anode materials for PIBs, including the reaction mechanism, phase transformations, structural evolution, and performance degradation. **Table** **5** comprehensively summarizes the electrochemical performances of cathode materials in PIBs.

**Table 5:** Summary of the electrochemical performances of cathode materials in PIBs.

Electrode material	Electrolyte	Rate performance	Discharge capability	Cycle capability	Ref.
KMO/CNT‐30	1 M KPF_6_	32% retention (0.5 A g^−1^)	309.4 mA h g^−1^ (20 mA g^−1^)	100 cycles, 108% retention	[229]
K_0.3_Mn_0.95_Co_0.05_O_2_	1 M KPF_6_	45% retention (0.44 A g^−1^)	99mAh g^−1^ (22 mA g^−1^)	500 cycles, 75% retention	[243]
V_2_O_3_@PNCNFs	0.8 M KPF_6_	55.8% retention (1 A g^−1^)	356.8mAh g^−1^ (50 mA g^−1^)	500 cycles, 95.8% retention	[244]
Od‐V_2_O_3_@C	1 M KFSI	59.7% retention (2 A g^−1^)	721.4mAh g^−1^ (100 mA g^−1^)	1000 cycles, 81.% retention	[245]
V_2_O_3_/CNF	3 M KFSI	42.% retention (10 A g^−1^)	410mAh g^−1^ (100 mA g^−1^)	2500 cycles, 98% retention	[246]
V_2_O_3_@C	1 M KFSI/DME	53.6% retention (5 A g^−1^)	816.6 mAh g^−1^ (100 mA g^−1^)	1800 cycles, 105% retention	[240]
Od‐V_2_O_3_@C	1 M KFSI	59.7% retention (5 A g^−1^)	721.4 mAh g^−1^ (100 mA g^−1^)	1000 cycles, 81.2% retention	[247]
VO_2_ (a)	2.5 M KFSI	65% retention (0.5 A g^−1^)	50 mAh g^−1^ (100 mA g^−1^)	8500 cycles, 80% retention	[248]
K_0.486_V_2_O_5_	1 M KCl	24% retention (2 A g^−1^)	94 mAh g^−1^ (100 mA g^−1^)	50 000 cycles, 53% retention	[249]
KFMHCF‐1/2	1 M KPF_6_	37.7% retention (0.2 A g^−1^)	155.3 mAh g^−1^ (10 mA g^−1^)	450 cycles, 66% retention	[242]
Ni_2_ZnHCF	0.6 M K_2_SO_4_	66.4% retention (60 A g^−1^)	58.7 mAh g^−1^ (10 A g^−1^)	5000 cycles, 73.2% retention	[250]
KMFON	1 M KPF_6_	36.7% retention (0.5 A g^−1^)	104.2 mAh g^−1^ (20 mA g^−1^)	300 cycles, 74.4% retention	[251]
KNFHCF‐1/2	1 M KPF_6_	65.5% retention (0.5 A g^−1^)	81.6 mAh g^−1^ (10 mA g^−1^)	200 cycles, 78.6% retention	[252]
K_x_FeFe(CN)_6_	0.5 M KPF_6_	_	140 mAh g^−1^ (10 mA g^−1^)	300 cycles, 60% retention	[254]
KMF‐40	0.8 M KPF_6_	61% retention (0.5 A g^−1^)	120.5mAh g^−1^ (100 mA g^−1^)	100 cycles, 74.3% retention	[255]
KFe[Fe(CN)_6_]	1 M KBF_4_	_	79mAh g^−1^ (8 mA g^−1^)	500 cycles, 88% retention	[256]
K_0.220_Fe[Fe(CN)_6_]_0.805_·4.01H2O	0.8 M KPF_6_	55.4% retention (0.4 A g^−1^)	73.2mAh g^−1^ (50 mA g^−1^)	150 cycles, 86.5% retention	[257]
K_1.68_Fe_1.09_Fe(CN)_6_·2.1H2O	0.8 M KPF_6_	_	110mAh g^−1^ (20 mA g^−1^)	100 cycles, 81% retention	[258]
Fe[Fe(CN)_6_]	1 M KPF_6_	73.5% retention (0.5 A g^−1^)	124mAh g^−1^ (71 mA g^−1^)	500 cycles, 93% retention	[259]
RGO@PB@SSM	0.8 M KPF_6_	42% retention (0.4 A g^−1^)	96.8mAh g^−1^ (10 mA g^−1^)	305 cycles, 75% retention	[260]
K_1.92_Fe[Fe(CN)_6_]_0.94_·0.5H2O	0.05 M KClO_4_	35.2% retention (0.5 A g^−1^)	133mAh g^−1^ (13 mA g^−1^)	200 cycles, 92.8% retention	[261]
KFeII[FeIII(CN)_6_]	1 M KPF_6_	_	118.7mAh g^−1^ (10 mA g^−1^)	100 cycles, 80.5% retention	[262]
K_4_Fe(CN)_6_/C	1 M KPF_6_	38.2% retention (0.2 A g^−1^)	66.5mAh g^−1^ (20 mA g^−1^)	400 cycles, 74.8% retention	[263]
K_0.61_Fe[Fe(CN)_6_]_0.91_·0.32H2O	0.8 M KPF_6_	77% retention (0.5 A g^−1^)	124mAh g^−1^ (10 mA g^−1^)	500 cycles, 69% retention	[264]
K_1.87_Fe[Fe(CN)_6_]_0.97_·0.84H_2_O@PPy	0.8 M KPF_6_	95% retention (0.05 A g^−1^)	88.9mAh g^−1^ (50 mA g^−1^)	500 cycles, 86.8% retention	[265]
K_1.4_Fe_4_[Fe(CN)_6_]_3_	0.5 M KPF_6_	35% retention (0.6 A g^−1^)	71mAh g^−1^ (50 mA g^−1^)	100 cycles, 72.5% retention	[266]

### 4.1 Cathode Materials

Tunnel‐structured cathode materials are instrumental in driving the progress of PIBs by facilitating fast K^+^ insertion/extraction processes and maintaining structural integrity over extended cycling periods. These materials offer a unique framework for efficient ion transport, addressing the challenges posed by the larger size of K^+^ ions compared to Li^+^ ions. In the subsequent sections, we embark on a comprehensive exploration of pivotal tunnel‐structured cathode materials using in situ TEM techniques. Through these investigations, we will illuminate the intricate processes governing their structural evolution, electrochemical behavior, and performance characteristics.

#### 4.1.1 Mn‐Based Oxides

**Figure 13:**
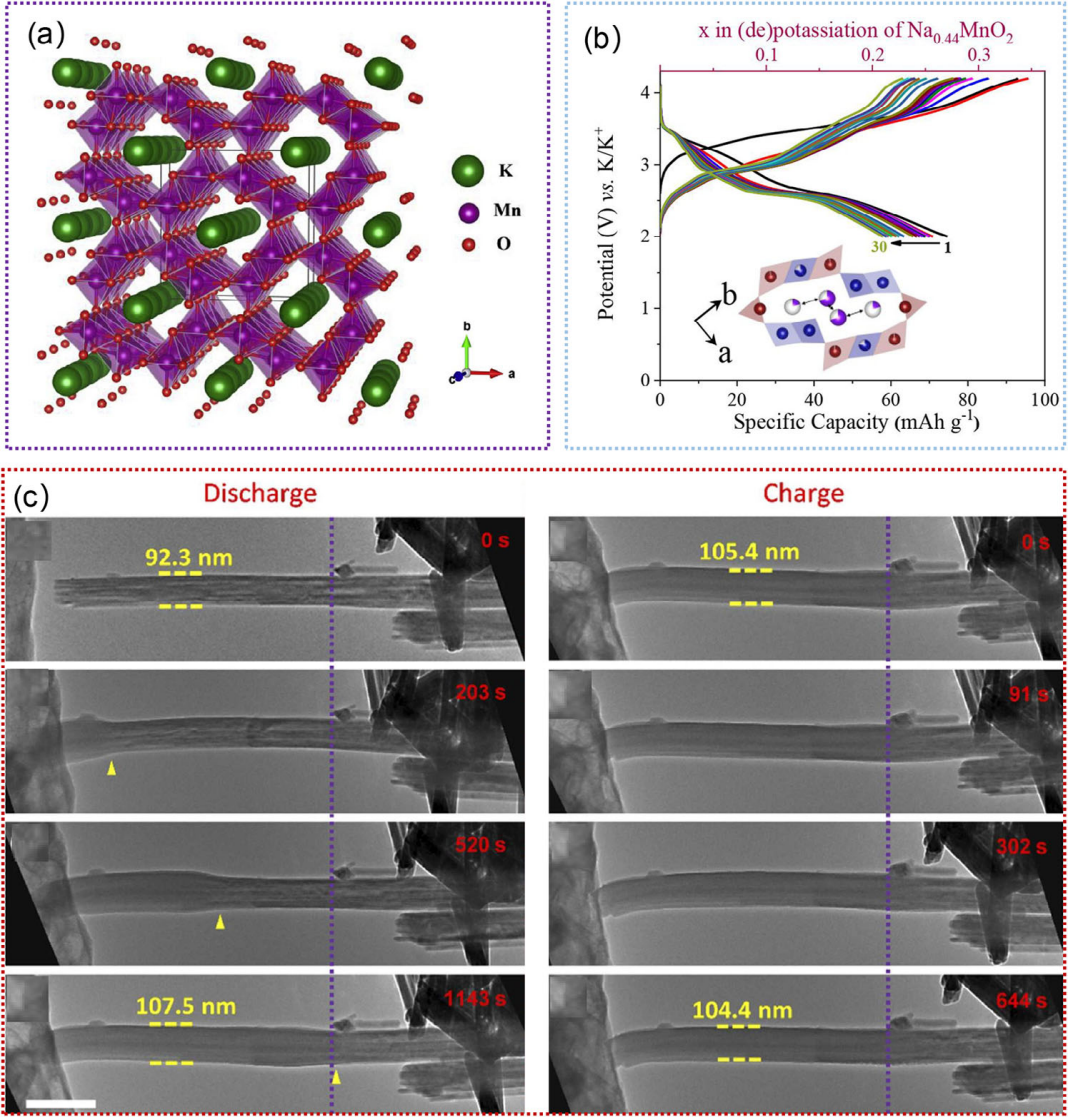
a) The corresponding crystal structural illustration of K_1.06_Mn_8_O_16_ along [001] direction. Reproduced with permission.^[^ ^229^ ^]^ Copyright 2018, Elsevier Ltd. b) The charge/discharge curves of Na_0.44_MnO_2_//K half‐cell, and the inset exhibits K^+^ interactions in the S‐tunnel after discharging. Reproduced with permission.^[^ ^235^ ^]^ Copyright 2022, American Chemical Society. c) In situ TEM studies of K‐(α‐MnO_2_) nanobattery at different charge/discharge states. Reproduced with permission.^[^ ^230^ ^]^ Copyright 2024, Elsevier Ltd.

Mn‐based oxides with unique tunnel structures possess the capability to accommodate K^+^ ions without undergoing fundamental structural breakdown, which exhibits great potential for advancing the development of high‐performance PIBs. Among them, the cryptomelane‐type compounds show molecular sieve structures constructed from double chains of edge‐shared MnO_6_ octahedral, forming unique (2 × 2) and (1 × 1) tunnels, as depicted in **Figure** **13**a.^[^ ^229^ ^]^ This characteristic makes them promising candidates for PIBs, offering the potential for high‐capability K^+^ ion storage with long‐term stability. Notably, 2 × 2 tunnels are often stabilized by adding cations (e.g., Na^+^, K^+^, NH^4+^, Ag^+^, etc.) during the synthesis process.^[^ ^230^, ^231^ ^]^ This step plays a crucial role in optimizing the structure and properties of the electrode materials for PIBs. For example, Vanam et al. demonstrated a tunnel‐type Na^+^ insertion material (Na_0.44_MnO_2_) by a solution combustion method, which exhibits a high capacity of 141 mAh g^−1^ (Figure 13b).^[^ ^235^ ^]^ Obviously, this deliberate design can effectively control the size and stability of the tunnels, leading to improved K^+^ ion diffusion kinetics and enhanced overall electrochemical performance of the material. By carefully tailoring the Na^+^ addition process, it is possible to optimize the cryptomelane‐type compounds for enhanced performance as electrode materials for PIBs, ultimately leading to improved energy storage capabilities and long‐term stability. Moreover, the in‐depth understanding revealed by in situ TEM provides invaluable insights that can serve as an instructive guide in the rational design of tunnel structures for high‐performance PIBs. For instance, Wang et al. demonstrated the irreversible volume expansion of α‐MnO_2_ nanowire during (de)potassiation processes by in situ TEM (Figure 13c), which provides a new approach to understanding the capacity loss mechanism during the (de)potassiation of α‐MnO_2_ cathodes.^[^ ^230^ ^]^ This analysis offers a direct visualization of the structural changes occurring at the nanoscale level, shedding light on the impact of volume expansion on the electrode material's long‐term performance. This new approach provides valuable insights that can guide the development of strategies to mitigate capacity loss and enhance the stability of α‐MnO_2_ cathodes in PIBs.

#### 4.1.2 V‐Based Oxides

**Figure 14:**
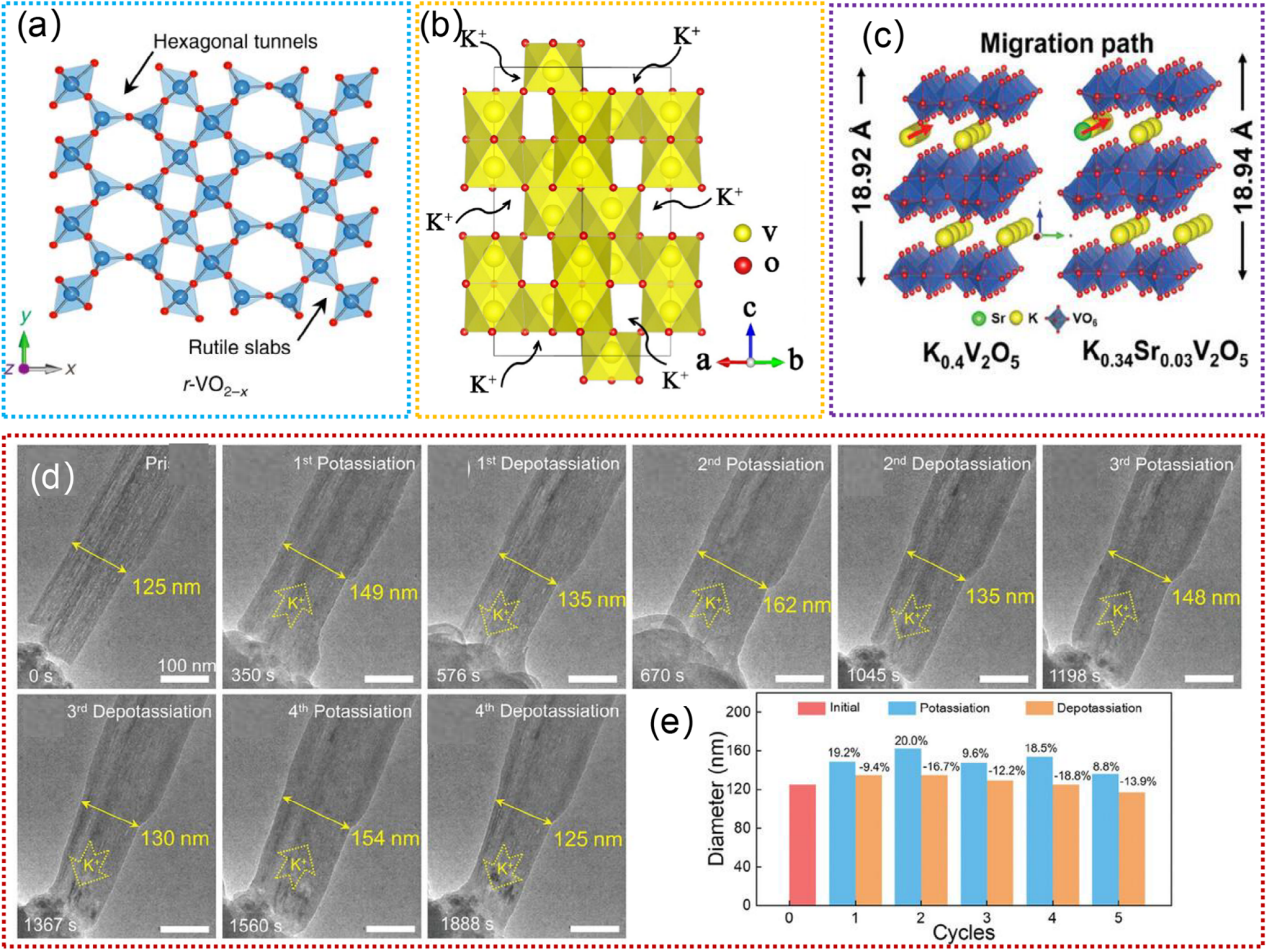
The a) atomic structure of the r‐VO_2_‐x. Reproduced with permission.^[^ ^270^ ^]^ Copyright 2018, Springer. b) Crystal structure model of V_2_O_3_. Reproduced with permission.^[^ ^244^ ^]^ Copyright 2018, Elsevier Ltd. c) The energy barrier and dedicated path of K^+^ migration of K_0.4_V_2_O_5_ and K_0.34_Sr_0.03_V_2_O_5_. Reproduced with permission.^[^ ^267^ ^]^ Copyright 2024, Wiley‐VCH GmbH. d) Schematic illustration of in situ experimental setup and snapshots of CaV_4_O_9_ during different (de)potassiation processes, and e) statistics of the cross sectional diameter changes versus the number of (de)potassiation cycles. Reproduced with permission.^[^ ^247^ ^]^ Copyright 2024, Wiley‐VCH GmbH.

Vanadium oxides (V_2_O_5_, V_2_O_3_, K_x_V_2_O_5_, etc.) are representative intercalation hosts and have been utilized in PIBs owing to their low cost and ability to exist in various oxidation states from V^2+^ to V^5+^.^[^ ^244^, ^247^, ^248^, ^249^, ^267^, ^268^, ^269^, ^270^, ^271^ ^]^ Besides, they present a notable distinction from conventional conversion‐type anodes due to their relatively small volume change when used as anodes, since the valence of vanadium rarely reaches zero at low voltage owing to the strong V‐O bond strength.^[^ ^267^ ^]^ For example, Liu et al. reported an isomeric vanadium oxide consisting of corundum‐type V_2_O_3_ and rutile r‐VO_2‐x_ core/shell structure, as illustrated in **Figure** **14**a.^[^ ^270^ ^]^ The r‐VO_2_ displays a tunnel structure that both K^+^ ions and electrons prefer to transport along the z‐axis tunnels, thus realizing high levels of energy storage at fast charging/discharging rates.^[^ ^270^ ^]^ In addition, V_2_O_3_ is also considered to be an attractive electrode material for PIBs due to its open tunnel structure consisting of a 3D V‐V framework (Figure 14b).^[^ ^244^ ^]^ This framework is well‐suited for efficiently facilitating the insertion of K^+^ ions, making V_2_O_3_ a promising candidate for use in high‐performance PIBs. Nevertheless, their practical applicability in PIBs is constrained by challenges such as low conductivity and drastic volume changes during extended cycling. Impressively, Oh et al. demonstrated that the potassium insertion K_0.4_V_2_O_5_ can effectively enhance both the electrical conductivity and potassium ion diffusion kinetics.^[^ ^267^ ^]^ Moreover, the introduction of Sr^2+^ ions in the K_0.4_V_2_O_5_ crystal could further reduce K^+^ ion diffusion energy barriers (Figure 14c), which significantly suppresses irreversible phase transition during the K^+^ ion storage process because of the robust interaction between Sr^2+^ and O^2−^.^[^ ^267^ ^]^ This advancement holds promise for addressing the limitations associated with vanadium oxide electrodes, potentially paving the way for improved performance and extended cycling stability in PIB applications. Recently, Wu et al. reported that porous CaV_4_O_9_ nanobelts can deliver a stable discharge capacity of 142 mAh g^−1^ at 0.1 A g^−1^ and excellent cycling life in the voltage range of 0.01–3 V (vs K^+^/K).^[^ ^247^ ^]^ Its fundamental reaction mechanisms associated with structural evolution and reaction kinetics were systematically investigated by in situ TEM, as illustrated in Figure 14d,e. The CaV_4_O_9_ nanobelts can buffer drastic stress accumulation and volume change during the (de)potassiation cycles, indicating its excellent structural stability.^[^ ^247^ ^]^ This characteristic suggests that CaV_4_O_9_ nanobelts possess an inherent ability to withstand the mechanical strains and volume fluctuations that occur during the insertion and extraction of K^+^ ions. Such structural robustness is a highly desirable trait in electrode materials for potassium‐ion batteries, as it can contribute to prolonged cycling stability and enhanced overall performance of the battery system. This new approach not only provides valuable insights into the intricate processes taking place within the anode materials but also offers guidance for the development of strategies aimed at enhancing the stability of vanadium oxides in PIBs.

#### 4.1.3 Prussian Blue Analogues

**Figure 15:**
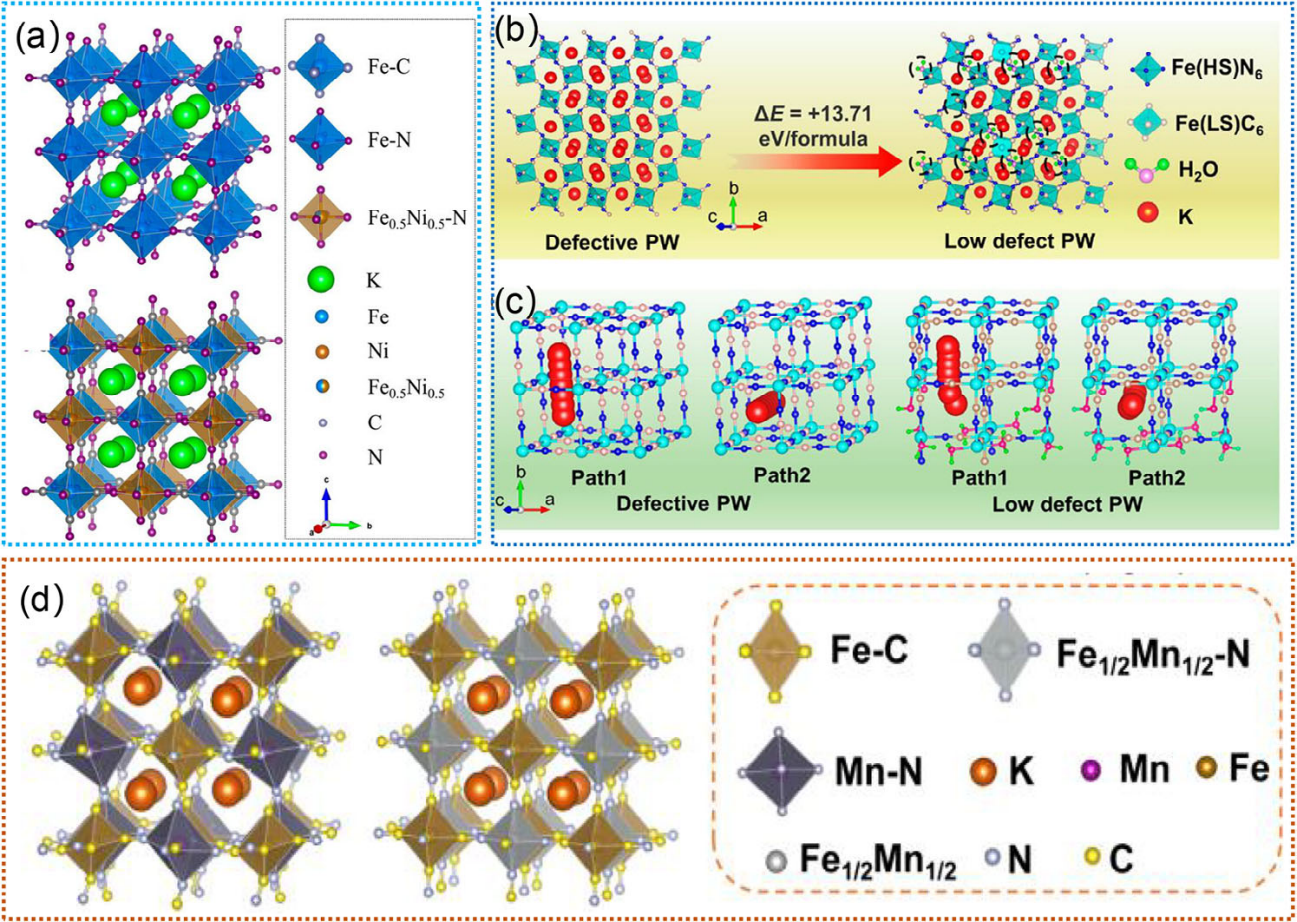
a) Crystal structure models of K_2_Fe[Fe(CN)_6_] and K_2_Ni_0.5_Fe_0.5_[Fe(CN)_6_]_0.89_·0.42H_2_O samples. Reproduced with permission.^[^ ^252^ ^]^ Copyright 2020, American Chemical Society. b) Calculated formation energy and c) possible K^+^ ion migration paths of the defective and low‐defect Prussian white structures. Reproduced with permission.^[^ ^275^ ^]^ Copyright 2023, American Chemical Society. d) The crystal structure models of K_2_Mn[Fe(CN)_6_] and K_1.47_Fe_0.5_Mn_0.5_[Fe(CN)_6_]·1.26H_2_O. Reproduced with permission.^[^ ^242^ ^]^ Copyright 2024, American Chemical Society.

Prussian blue analogues (PBAs) have been widely studied as electrode materials for PIBs due to their open 3D framework and easily adjustable composition, which facilitates rapid K^+^ ion insertion/extraction.^[^ ^250^, ^251^, ^272^, ^273^, ^274^, ^275^ ^]^ Unfortunately, the rapid nucleation and growth rate can lead to the formation of [Fe(CN)_6_]^4−^ vacancies, ultimately reducing the active sites and causing structural deterioration during cycling.^[^ ^251^, ^273^, ^274^, ^275^ ^]^ Additionally, complex phase transitions occurring during the K^+^ (de)insertion process indeed result in low specific capacity and poor cycling stability.^[^ ^242^, ^252^, ^275^ ^]^ These challenges underscore the need for further research and development to address these issues and optimize the performance of PBAs in PIB applications. To address these challenges, researchers have explored various optimization methods, including the incorporation of specific agents during synthesis and the design of multielement components. For example, Huang et al. reported Ni_x_Zn_y_HCF bimetallic PBAs that demonstrated a synergistic effect between stable Ni^2+^ and high‐voltage Zn^2+^ in Ni_2_Zn_1_HCF, which shows ultrafast near‐pseudocapacitance intercalation and super‐stable potassium storage.^[^ ^250^ ^]^ Chong et al. designed Ni‐substituted PBAs by a one‐step hydrothermal approach (**Figure** **15**a), which displays abundant electrochemically active Fe‐ions, exceptional electrochemical kinetics, and admirable structural stability attributed to inert Ni‐ions.^[^ ^252^ ^]^ Li et al. proposed a template‐engaged reduction method by using KI as the reducing agent to prepare K‐rich Prussian white with low defects and water content.^[^ ^275^ ^]^ It's fascinating that the research suggests that low‐defect Prussian white exhibits a more stable structure (as depicted in Figure 15b), and lower energy barriers (as illustrated in Figure 15c).^[^ ^275^ ^]^ This finding underscores the importance of defect reduction in enhancing the stability and overall performance of Prussian white, further highlighting the significance of defect engineering in the development of advanced materials for PIB applications. Despite their effectiveness, the high cost associated with chelating agents poses significant challenges for large‐scale industrialization. Recently, Zhou et al. proposed a defect‐free potassium iron manganese hexacyanoferrate (K_1.47_Fe_0.5_Mn_0.5_[Fe(CN)_6_]·1.26H_2_O, KFMHCF‐1/2) as the electrode material for PIBs.^[^ ^242^ ^]^ As‐prepared KFMHCF‐1/2 exhibits a 3D open framework comprising N‐coordinated Mn or Fe_0.5_Mn_0.5_ and C‐coordinated Fe via connecting alternately with cyano‐groups, as illustrated in Figure 15d.^[^ ^242^ ^]^ The Fe‐Mn binary synergistic and defect‐free effects play a crucial role in inhibiting cell volume change and octahedral slip during the K^+^ ion (de)insertion process. This inhibition effectively prevents phase transformation behavior (monoclinic ↔ cubic), leading to a zero‐strain solid solution mechanism.^[^ ^242^ ^]^ By employing Fe and Mn as dual active sites, this approach offers a promising strategy to achieve enhanced stability and structural integrity. Although PBA materials play a pivotal role in the operation of PIBs, the precise reaction mechanisms occurring within this structure are not yet fully understood. Continued research efforts utilizing advanced methods such as in situ TEM, XRD, and other analytical tools are crucial for unraveling the intricacies of the reaction mechanisms within the 3D open framework of PBA materials. This deeper insight into the reaction mechanisms and structural changes during the operation of PIBs will enable the design and optimization of next‐generation energy storage materials with improved performance, stability, and efficiency.

### 4.2 Anode Materials

Tunnel‐structured anode materials are essential components in PIBs, offering a vital framework to house the substantial influx of K^+^ ions during charge and discharge cycles, all while maintaining structural integrity. In the following section, we will delve into the examination of critical anode materials studied in PIBs using in situ TEM techniques. **Table** **6** comprehensively summarizes the electrochemical performances of anode materials in PIBs.

**Table 6:** Summary of the electrochemical performances of anode materials in PIBs.

Electrode material	Electrolyte	Rate performance	Discharge capability	Cycle capability	Ref.
K_0.17_TiO_2_	0.5 M KFSI	87% retention (1.55 A g^−1^)	79 mAh g^−1^ (15.5 mA g^−1^)	1000 cycles, 98% retention	[236]
K_2_Ti_6_O_13_	1 M KFSI	93% retention (0.05 A g^−1^)	190.7 mAh g^−1^ (10 mA g^−1^)	200 cycles, 77.3% retention	[239]
G‐TiO_2_ NTs	0.8 M KPF_6_	47.57% retention (5 A g^−1^)	831 mAh g^−1^ (50 mA g^−1^)	400 cycles, 84.1% retention	[276]
TiO_2_@NGC	1 M KPF_6_	50% retention (1 A g^−1^)	518 mAh g^−1^ (50 mA g^−1^)	2000 cycles, 98% retention	[277]
K_0.5_Mn_0.92_Ti_0.08_O_2_	0.8 M KPF_6_	_	126.9mAh g^−1^ (20 mA g^−1^)	100 cycles, 53.7% retention	[278]
M‐KTO	1 M KPF_6_	54% retention (0.3 A g^−1^)	584 mAh g^−1^ (50 mA g^−1^)	900 cycles, 51% retention	[279]
K_2_Ti_8_O_17_	0.8 M KPF_6_	24.35% retention (0.5 A g^−1^)	181.5mAh g^−1^ (20 mA g^−1^)	50 cycles, 60.9% retention	[280]
KTO	0.8 M KPF_6_	67.36% retention (0.5 A g^−1^)	267mAh g^−1^ (50 mA g^−1^)	1000 cycles, 84% retention	[281]
TiO_2_@rGO‐1	0.8 M KPF_6_	37% retention (5 A g^−1^)	1829.9mAh g^−1^ (50 mA g^−1^)	800 cycles, 79% retention	[282]
G‐TiO_2_ NTs	0.8 M KPF_6_	47.56% retention (5 A g^−1^)	831mAh g^−1^ (50 mA g^−1^)	400 cycles, 85.8% retention	[283]
TiO_2e_C	0.8 M KPF_6_	19.68% retention (2 A g^−1^)	186.4mAh g^−1^ (200 mA g^−1^)	900 cycles, 28.6% retention	[284]
TiO_2_@NGC	1 M KPF_6_	50% retention (1 A g^−1^)	518mAh g^−1^ (50 mA g^−1^)	2000 cycles, 98% retention	[285]
KTP@C	0.8 M KPF_6_	_	131mAh g^−1^ (1000 mA g^−1^)	1000 cycles, 53.2% retention	[242]

**Figure 16:**
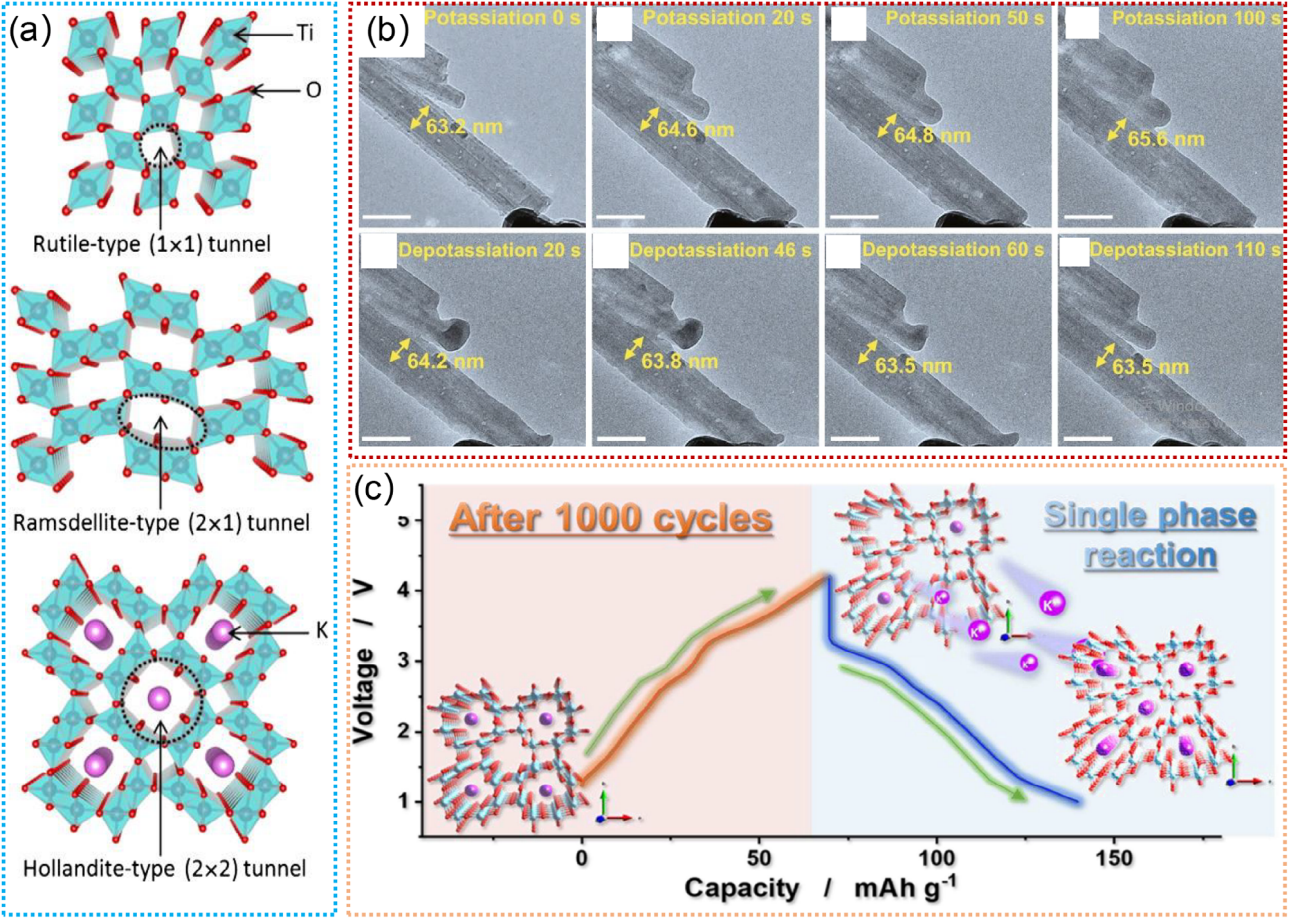
a) Schematic representation of 1D tunnel structures of hollandite‐type TiO_2_. Reproduced with permission.^[^ ^288^ ^]^ Copyright 2013, Elsevier Ltd. (b) In situ TEM study of G‐TiO_2_ NTs during (de)potassiation process. Reproduced with permission.^[^ ^286^ ^]^ Copyright 2020, Springer. c) Schematic illustration of cycling stability of K_0.17_TiO_2_. Reproduced with permission.^[^ ^236^ ^]^ Copyright 2023, Elsevier Ltd.

Ti‐based oxides (TiO_2_, K_2_Ti_6_O_13_, K_2_Ti_4_O_9_, K_2_Ti_8_O_17_, etc.) have garnered significant attention as anode materials for PIBs due to their intrinsic structural advantages, including low cost, fantastic chemical and thermal stability.^[^ ^271^, ^286^, ^287^, ^288^, ^289^, ^290^, ^291^, ^292^, ^293^, ^294^ ^]^ Generally, hollandite‐type TiO_2_ features large tunnels composed of single or multiple chains of edge‐sharing TiO_6_ octahedra, as illustrated in **Figure** **16**a.^[^ ^288^ ^]^ The unique structural framework allows it to store a larger quantity of K^+^ ions, while also offering shorter diffusion path lengths. Nevertheless, pure TiO_2_ usually exhibits poor conductivity and sluggish K^+^ reaction kinetics, which significantly limits its performance as an electrode material for PIBs. Incorporating cations (Na^+^, K^+^, etc.) and carbon coating modification are considered effective strategies for improving performance.^[^ ^271^, ^286^, ^287^, ^288^, ^289^, ^290^, ^291^, ^292^, ^293^, ^294^ ^]^ For example, Dubal et al. reported the ultrafine TiO_2_ nanoparticle supported nitrogen‐rich graphitic porous carbon as an anode material for PIBs, which not only shows a high reversible specific capacity (228 mAh g^−1^ at 0.05 A g^−1^) with excellent cyclic stability but also displays significantly enhanced rate capability.^[^ ^277^ ^]^ Cai et al. proposed the graphene‐armored TiO_2_ nanotubes (G‐TiO_2_ NTs), which display a high reversible capacity of 332 mAh g^−1^ at 0.05 A g^−1^.^[^ ^286^ ^]^ Besides, they also studied the structural evolutions of G‐TiO_2_ NTs during the (de)potassiation processes by in situ TEM, as displayed in Figure 16b.^[^ ^286^ ^]^ No visible crack or fracture could be observed in the whole process, revealing the favorable structural stability. Li et al. designed the fluff‐like hydrogenated Na_2_Ti_3_O_7_ nanowires grown on N‐doped carbon sponge (HNOT/CS), which exhibits a highly reversible specific capacity (107.8 mAh g^−1^ at 0.1 A g^−1^) and superior cycle stability.^[^ ^289^ ^]^ Interestingly, Jo et al. designed the hollandite‐type K_0.17_TiO_2_ with exceptional cycling stability as a cathode material for PIBs.^[^ ^236^ ^]^ As shown in Figure 16c, the K^+^ can be accommodated within the (2 × 2) tunnels along the c‐axis in K_0.17_TiO_2_, which underwent a single‐phase reaction accompanied by the Ti^4+/3+^ redox pair during K^+^ insertion.^[^ ^236^ ^]^ As‐designed K_0.17_TiO_2_ cathode can deliver a specific capacity of 79 mAh g^−1^ at 0.05 C with excellent capacity retention of 98% for 1000 cycles.^[^ ^236^ ^]^ By focusing on these optimization strategies, researchers can gain a comprehensive understanding of how carbon coating modification and cation incorporation processes influence the structural stability of Ti‐based materials. More importantly, utilizing advanced characterization techniques such as in situ (s)TEM and XRD can significantly advance understanding of the structural integrity and electrochemical behavior of Ti‐based materials, thereby facilitating targeted optimization strategies to enhance their performance as anode materials for PIBs.

## 5 Integration of In Situ TEM with Other Electrochemical Techniques

In situ TEM techniques, while powerful, have inherent limitations such as restricted sampling areas and potential electron beam artifacts. To overcome these challenges and gain a holistic understanding of electrochemical processes, it is essential to combine in situ TEM with complementary electrochemical and characterization techniques, such as XRD, Raman, X‐ray photoelectron spectroscopy (XPS), nuclear magnetic resonance (NMR), Neutron diffraction (ND), X‐ray absorption near‐edge structures (XANES), cyclic voltammetry (CV), Galvanostatic charge‐discharge (GCD) and electrochemical impedance spectroscopy (EIS).^[^ ^29^, ^30^, ^31^, ^32^, ^33^, ^34^, ^35^, ^36^, ^37^, ^38^ ^]^ For example, the integration of in situ TEM with XRD enables the tracking of crystallographic changes and phase transformations throughout electrochemical cycling, providing a comprehensive understanding of structural evolution. By incorporating NMR spectroscopy, researchers can investigate ion dynamics and local environments, complementing the structural insights offered by in situ TEM. Additionally, combining Raman spectroscopy with in situ TEM allows for the real‐time monitoring of chemical bonding and molecular changes during cycling, offering a holistic view of the electrochemical processes at the molecular level. These multi‐technique approaches not only enhance the depth of analysis but also provide synergistic insights into the dynamic behavior of electrode materials in energy storage applications. By integrating these advanced analytical tools, researchers can overcome the limitations of individual methods and gain a more comprehensive understanding of the structural, chemical, and electrochemical processes in battery materials. This multi‐technique approach will accelerate the development of advanced energy storage systems with improved performance and stability.

## 6 Summary and Outlook

**Figure 17:**
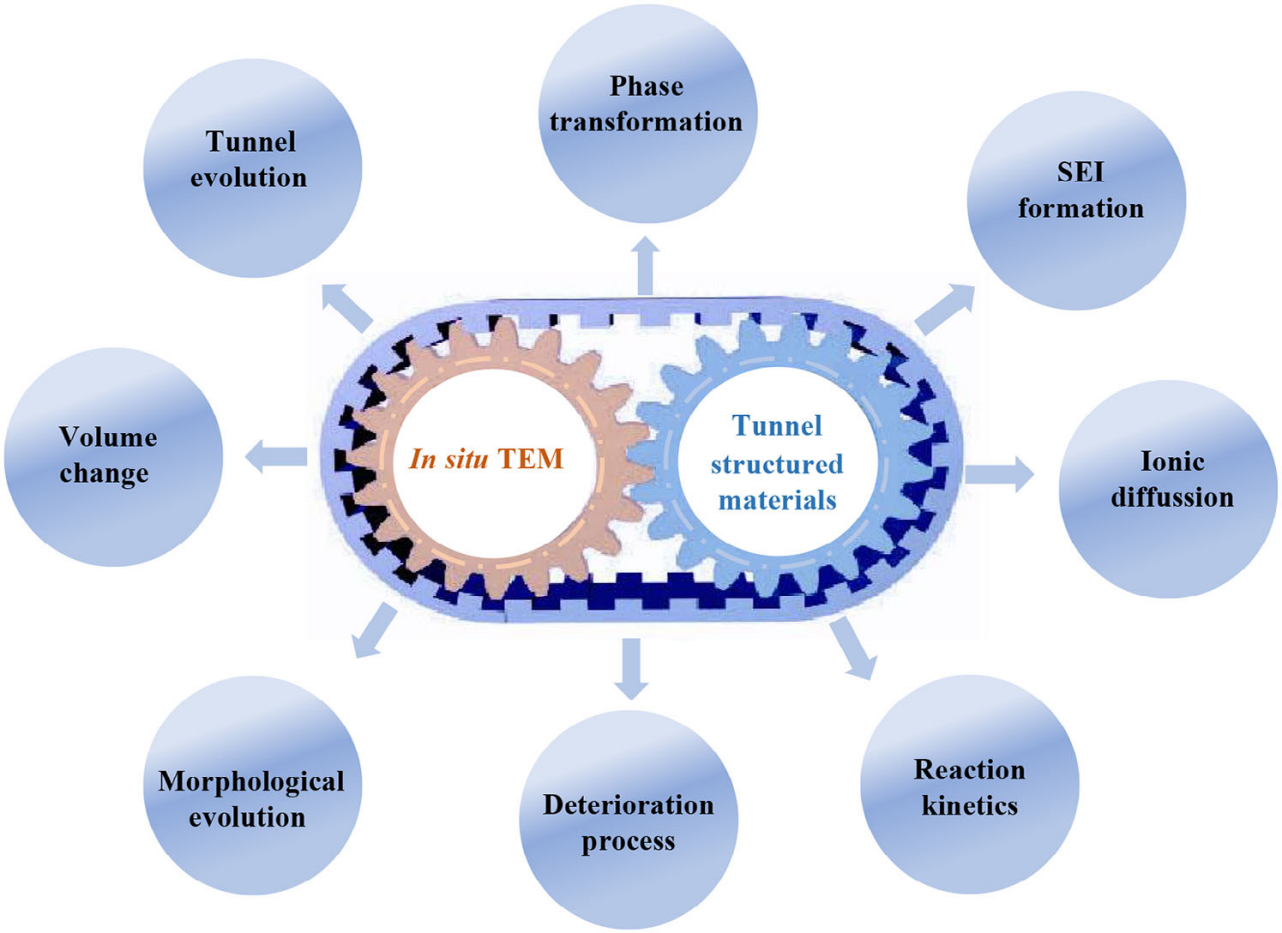
In Situ TEM techniques used for studying the tunnel‐structured materials for alkali metal‐ion batteries.

This review highlights the recent advancements of in situ TEM techniques for studying electrode materials in advanced AMIBs (**Figure** **17**), focusing on morphological and structural evolution, phase transformations, ionic diffusion, reaction kinetics, and interfacial dynamics. These studies provide fundamental insights into electrochemical mechanisms, degradation, and failure during cycling. However, challenges remain in translating nanoscale observations to practical battery performance. Key areas for future research include:

1. **Bridging the gap between nanoscale and macroscale**: Enhance in situ TEM setups to incorporate practical liquid electrolytes and electrode configurations used in macroscopic AMIBs, ensuring more representative insights into real‐world battery behavior.
2. **Long‐term cycling studies**: Develop advanced in situ TEM techniques and accelerated testing methodologies to enable real‐time observation of material evolution and degradation over extended cycling periods.
3. **Minimizing electron beam artifacts**: Design specialized TEM holders and miniaturized electrochemical cells to simulate realistic battery conditions while minimizing electron beam interference.
4. **Improving voltage control**: Address challenges in measuring and controlling voltage by combining open‐cell and liquid‐cell setups for correlative observations, enabling a more comprehensive understanding of electrochemical processes.
5. **Probing interfacial properties**: Utilize advanced techniques like TEM‐based electron holography and 4D‐STEM to study electrode/electrolyte interfaces with atomic resolution, providing insights into ionic conduction and charge transport.
6. **Multi‐technique characterization**: Combine in situ TEM with complementary techniques (XRD, ND, XANES, NMR, Raman spectroscopy) to overcome sampling limitations and gain a holistic understanding of material transformations and electrochemical processes.

Overall, in situ TEM has significantly advanced the understanding of tunnel‐structured electrode materials in AMIBs. Continued innovation in this field promises to unlock new battery chemistries and materials, driving the development of next‐generation energy storage technologies.

## Conflict of Interest

The authors declare no conflict of interest.

## References

[1] S. Li , J. Zhang , S. Zhang , Q. Liu , H. Cheng , L. Fan , W. Zhang , X. Wang , Q. Wu , Y. Lu , Nat. Energy 2024, 9, 285.

[2] P. Xiong , F. Zhang , X. Zhang , S. Wang , H. Liu , B. Sun , J. Zhang , Y. Sun , R. Ma , Y. Bando , C. Zhou , Z. Liu , T. Sasaki , G. Wang , Nat. Commun. 2020, 11, 3297. 32620745 10.1038/s41467-020-17014-w PMC7335097

[3] M. Liang , T. Xia , H. Gao , K. Zhao , T. Cao , M. Deng , X. Ren , S. Li , H. Guo , R. Wang , Nano Res. 2022, 15, 1221.

[4] F. Ma , Y. Wu , S. Dai , P. Lin , J. Sun , L. Dong , Nano Res. 2024, 17, 6567.

[5] Y. Liang , Y. Yao , Nat. Rev. Mater. 2023, 8, 109.

[6] S. G. Dai , Z. L. Lin , H. Hu , Y. Wang , L. H. Zeng , Appl. Phys. Rev. 2024, 11, 041319.

[7] D. Edelman , D. Eum , W. C. Chueh , Nat. Sustainability 2024, 7, 234.

[8] B. Wang , J. Fitzpatrick , A. Brookfield , A. Fielding , E. Reynolds , J. Entwistle , J. Tong , B. Spencer , S. Baldock , K. Hunter , C. Kavanagh , N. Tapia‐Ruiz , Nat. Commun. 2024, 15, 3013. 38589362 10.1038/s41467-024-45460-3 PMC11001870

[9] X. Li , X. Zhang , J. Xu , Z. Duan , Y. Xu , X. Zhang , L. Zhang , Y. Wang , P. Chu , Adv. Sci. 2024, 11, 2305467. 10.1002/advs.202305467 PMC10837388 38059813

[10] Y. Zhang , J. Wang , L. Shan , B. Han , Q. Gao , Z. Cai , C. Zhou , X. Tian , R. Sun , L. Mai , Adv. Energy Mater. 2023, 13, 2303464.

[11] X. Wu , S. Li , B. Yang , C. Wang , Electrochem. Energy Rev. 2019, 2, 467.

[12] Z. Chen , X. Wu , Z. Sun , J. Pan , J. Han , Y. Wang , H. Liu , Y. Shen , J. Li , D. Peng , Q. Zhang , Adv. Energy Mater. 2024, 14, 2400132.

[13] A. Tripathi , W. Su , B. J Hwang , Chem. Soc. Rev. 2018, 47, 736. 29308803 10.1039/c7cs00180k

[14] S. G. Dai , Z. F. Zhang , J. M. Xua , W. X. Shen , Q. B. Zhang , X. G. Yang , T. T. Xu , D. Dang , H. Hu , B. Zhao , Y. Wang , C. Qu , J. W. Fu , X. J. Li , C. G. Hu , M. L. Liu , Nano Energy 2019, 64, 103919.

[15] Y. Fang , D. Luan , X. Lou , Adv. Mater. 2020, 32, 2002976. 10.1002/adma.202002976 32914499

[16] C. Wang , X. Wang , R. Zhang , T. Lei , K. Kisslinger , H. Xin , Nat. Mater. 2023, 22, 235. 36702885 10.1038/s41563-022-01461-5

[17] Y. Yuan , C. Zhan , K. He , H. Chen , W. Yao , S. Sharifi‐Asl , B. Song , Z. Yang , A. Nie , X. Luo , H. Wang , S. Wood , K. Amine , M. Saiful Islam , J. Lu , R. Shahbazian‐Yassar , Nat. Commun. 2016, 7, 13374. 27869120 10.1038/ncomms13374 PMC5473628

[18] C. Li , Y. Yuan , P. Li , K. Yang , Q. Ren , A. Nie , S. Liu , S. Lazar , A. Meingast , S. Wang , ACS Nano 2022, 16, 21618. 36521057 10.1021/acsnano.2c10682

[19] S. Dai , W. Xu , Y. Xin , M. Wang , X. Gun , D. Guo , C. Hu , Nano Energy 2016, 19, 363.

[20] W. Shen , J. Zang , H. Hu , J. Xu , Z. Zhang , R. Yan , S. Dai , Mater. Des. 2020, 195, 108992.

[21] J. Hyeon Jo , J. U Choi , M. K Cho , Y. Aniskevich , H. Kim , G. Ragoisha , E. Streltsov , J. Kim , S. Myung , Adv. Energy Mater. 2019, 9, 1900603.

[22] G. Zhao , G. Wan , Y. Tang , X. Xu , X. Zhou , M. Zhou , Z. Deng , S. Lina , G. Wang , Chem. Commun. 2020, 56, 12435. 10.1039/d0cc04762g 32939519

[23] S. Choe , C. Yu , K. Ri , J. Kim , U. Jong , Y. Kye , S. Hong , Phys. Chem. Chem. Phys. 2019, 21, 8408. 30942793 10.1039/c9cp00267g

[24] S. Yang , K. R. Tallman , P. Liu , D. M. Lutz , B. Zhang , S. Joo Kim , L. Wu , A. C. Marschilok , E. S. Takeuchi , K. J. Takeuchi , Y. Zhu , Nano Energy 2020, 71, 104571.

[25] J. Cao , D. Zhang , Y. Yue , X. Wang , T. Pakornchote , T. Bovornratanaraks , X. Zhang , Z. Wu , J. Qin , Nano Energy 2021, 84, 105876.

[26] S. G. Dai , Y. Xi , C. G. Hu , J. L. Liu , K. Y. Zhang , X. L. Yue , L. Cheng , J. Mater. Chem. A 2013, 1, 15530.

[27] Y. Yuan , K. Amine , J. Lu , R. Shahbazian‐Yassar , Nat. Commun. 2017, 8, 15806.

[28] S. Dai , J. Zhang , J. Qu , X. Li , S. Cheng , C. Shan , Renewables 2023, 1, 57.

[29] Z. Lin , K. Fan , T. Liu , Z. Xu , G. Chen , H. Zhang , H. Li , X. Guo , X. Zhang , Y. Zhu , P. Hou , H. Huang , Nano‐Micro Lett. 2024, 16, 48. 10.1007/s40820-023-01269-1 PMC10713914 38082174

[30] L. Hou , L. Zhang , J. Zang , W. Shen , T. Zhang , X. Huang , H. Yuan , D. Kong , Y. Wang , X. Li , T. Xu , J. Phys,. D 2022, 55, 234002.

[31] X. Li , J. Fu , Y. Sun , M. Sun , S. Cheng , K. Chen , X. Yang , Q. Lou , T. Xu , Y. Shang , J. Xu , Q. Chen , C. Shan , Nanoscale 2019, 11, 13343. 31271407 10.1039/c9nr03581h

[32] Y. Wang , Y. V. Lim , S. Huang , M. Ding , D. Kong , Y. Pei , T. Xu , Y. Shi , X. Li , H. Yang , Nanoscale 2020, 12, 4341. 31994571 10.1039/c9nr09278a

[33] H. Zhao , C. Zhuang , J. Xu , Z. Zhang , W. Shen , H. Tang , Y. Wang , T. Xu , X. Wang , X. Li , Ionics 2020, 26, 5019.

[34] X. Li , C. Zhuang , J. Xu , L. Li , T. Xu , S. Dai , X. Wang , X. Li , Y. Wang , Nanoscale 2021, 13, 8199. 33885119 10.1039/d1nr00993a

[35] S. Dai , Z. Zhang , J. Xu , W. Shen , Q. Zhang , X. Yang , T. Xu , D. Dang , H. Hu , B. Zhao , Y. Wang , C. Qu , J. Fu , X. Li , C. Hu , M. Liu , Nano Energy 2019, 64, 103919.

[36] Y. Su , X. Lei , Z. Han , H. Liu , J. Xiao , Y. Su , S. Ren , Y. Lin , Q. Hu , R. Yang , G. Zhou , D. Su , Y. Zhang , Nano Lett. 2024, 24, 5332. 38634554 10.1021/acs.nanolett.4c01183

[37] C. Sun , B. Zhao , Z. Jing , H. Zhang , Q. Wen , H. Chen , X. Zhang , J. Zheng , Adv. Sci. 2024, 11, 2309657. 10.1002/advs.202309657 PMC11220708 38654462

[38] K. J. Chen , J. H. Zang , Z. F. Zhang , Y. Wang , Q. Lou , Y. C. Bai , J. Fu , C. F. Zhuang , Y. Zhang , L. L. Zhang , S. G. Dai , C. X. Shan , Nanoscale 2021, 13, 12370. 34254619 10.1039/d1nr02158c

[39] Z. Fan , L. Zhang , D. Baumann , L. Mei , Y. Yao , X. Duan , Y. Shi , J. Huang , Y. Huang , X. Duan , Adv. Mater. 2019, 31, 1900608. 10.1002/adma.201900608 31183914

[40] J. Cui , H. Zheng , K. He , Adv. Mater. 2021, 33, 2000699. 10.1002/adma.202000699 32578290

[41] Z. X. Huang , Z. X. Wang , X. C. Wang , S. Zhang , T. T. Xu , Z. F. Zhang , J. H. Zang , D. Z. Kong , X. J. Li , Y. Wang , Solid State Ionics 2022, 380, 115941.

[42] B. F. Tian , Z. X. Huang , H. Y. Yang , H. Wang , T. T. Xu , D. Z. Kong , C. J. Gao , J. H. Zang , X. J. Li , Y. Wang , Ionics 2022, 28, 4641.

[43] P. Liu , Q. Chen , Y. Ito , J. Han , S. Chu , X. Wang , K. M Reddy , S. Song , A. Hirata , M. Chen , Nano Lett. 2020, 20, 1944. 32069418 10.1021/acs.nanolett.9b05216

[44] S. G. Dai , J. W. Zhang , J. Q. Qu , X. Li , S. B. Cheng , C. X. Shan , Renewables 2023, 1, 57.

[45] O. Kwon , T. Y Kim , T. Kim , J. Kang , S. Jang , H. Eom , S. Choi , J. Shin , J. Park , M. Seol , J. W Han , S. Park , H. Lee , I. Nam , Adv. Energy Mater. 2024, 14, 2304085.

[46] B. Y. Wang , T. T. Jiang , L. J. Hou , H. Wang , T. T. Xu , Z. F. Zhang , D. Z. Kong , X. J. Li , Y. Wang , Solid State Ionics 2021, 368, 115711.

[47] Y. Li , H. Xu , Q. Ning , S. Li , J. Wang , J. Wang , Z. Hu , J. Tian , X. Li , Y. Han , Y. Zhu , Adv. Funct. Mater. 2024, 34, 2401361.

[48] J. Xu , H. Tang , T. Xu , D. Wu , Z. Shi , Y. Tian , X. Li , Ionics 2017, 23, 3273.

[49] J. Tang , B. Zhao , Z. Wang , J. Li , S. Guo , J. Shin , M. Wang , Y. Deng , ACS Appl. Mater. Interfaces 2024, 16, 16075. 38527926 10.1021/acsami.3c18027

[50] J. Huang , L. Zhong , C. Wang , J. P. Sullivan , W. Xu , L. Zhang , S. X. Mao , N. S. Hudak , X. Liu , A. Subramanian , H. Fan , L. Qi , A. Kushima , J. Li , Science 2010, 330, 1515. 21148385 10.1126/science.1195628

[51] M. Gu , L. R. Parent , B. Layla Mehdi , R. Unocic , M. T. McDowell , R. L. Sacci , W. Xu , J. Grant Connell , P. Xu , P. Abellan , X. Chen , Y. Zhang , D. E. Perea , J. E. Evans , L. J. Lauhon , J. Zhang , J. Liu , N. D. Browning , Y. Cui , I. Arslan , C. Wang , Nano Lett. 2013, 13, 6106. 24224495 10.1021/nl403402q

[52] J. Xie , J. Li , W. Mai , G. Hong , Nano Energy 2021, 83, 105780.

[53] J. M. Yuk , H. K. Seo , J. W. Choi , J. Y. Lee , ACS Nano 2014, 8, 7478. 24980889 10.1021/nn502779n

[54] L. Luo , B. Liu , S. Song , W. Xu , J. G. Zhang , C. Wang , Nat. Nanotechnol. 2017, 12, 535. 28346458 10.1038/nnano.2017.27

[55] Y. Li , Y. Li , A. Pei , K. Yan , Y. Sun , C. Wu , L. Joubert , R. Chin , A. Leen Koh , Y. Yu , J. Perrino , B. Butz , S. Chu , Y. Cui , Science 2017, 358, 506. 29074771 10.1126/science.aam6014

[56] M. J. Zachman , Z. Tu , S. Choudhury , L. A. Arche , L. F. Kourkoutis , Nat. 2018, 560, 345. 10.1038/s41586-018-0397-3 30111789

[57] Y. Xie , J. Wang , B. Savitzky , Z. Chen , Y. Wang , S. Betzler , K. Bustillo , K. Persson , Y. Cui , L. Wang , C. Ophus , P. Ercius , H. Zheng , Sci. Adv. 2023, 9, 9721. 10.1126/sciadv.adc9721 PMC11706249 36638171

[58] K. Zhang , H. Wang , X. Du , S. Dai , Y. Wang , T. Xu , M. Liu , S. Cheng , Adv. Funct. Mater. 2024, 34, 2407105.

[59] X. Ma , D. Dong , S. Guo , N. Cheng , B. Zhang , B. Ge , Adv. Funct. Mater. 2024, 34, 2400779.

[60] Y. Li , F. Börrnert , M. Ghorbani‐Asl , J. Biskupek , X. Zhang , Y. Zhang , D. Bresser , A. V. Krasheninnikov , U. Kaiser , Adv. Funct. Mater. 2024, 34, 2406034.

[61] S. Wei , J. Shang , Y. Zheng , T. Wang , X. Kong , Q. He , Z. Zhang , Y. Zhao , J. Colloid Interface Sci. 2024, 675, 904. 39002240 10.1016/j.jcis.2024.07.079

[62] M. Xu , P. Yang , K. Fan , Y. Gao , Z. Zhang , Y. Li , X. Li , J. Qi , K. Xi , H. Huang , L. Fei , Nano Today 2024, 57, 102393.

[63] L. Cheng , X. Luo , B. Ge , ACS Appl. Mater. Interfaces 2024, 16, 35006. 38935752 10.1021/acsami.4c05433

[64] Y. Liu , J. Huang , Mater. Today Commun. 2024, 39, 108780.

[65] D. Yang , R. Huang , B. Zou , X. Zhang , E. Huixiang Ang , Y. Wang , Y. Sun , H. Xiang , X. Song , Nano Today 2024, 57, 102316.

[66] J. Wu , H. Cai , Z. Deng , J. Gaumet , Y. Bao , W. Luo , Rare Met. 2024, 43, 3553.

[67] J. Li , J. Chen , X. Xu , J. Sun , B. Huang , T. Zhao , Energy Environ. Sci. 2024, 17, 5521.

[68] Y. Hu , Y. Nian , M. Wang , N. Wang , Y. Qiu , L. Zhang , X. Li , Y. Han , L. Luo , ACS Mater. Lett. 2024, 6, 3335.

[69] H. Hu , R. Yang , Z. Zeng , ACS Nano 2024, 18, 12598. 38723158 10.1021/acsnano.4c03319

[70] A. Minenkov , N. Šantić , H. Groiss , Microsc. Microanal. 2022, 28, 2300.

[71] S. Eswara , A. Pshenova , L. Yedra , Q. H. Hoang , J. Lovric , P. Philipp , T. Wirtz , Appl. Phys. Rev. 2019, 6, 021312.

[72] Y. Xu , Y. Xu , X. Han , S. Wang , J. Yu , J. Mater. Chem. A 2021, 9, 24397.

[73] Q. Xia , Q. Zhang , S. Sun , F. Hussain , C. Zhang , X. Zhu , F. Meng , K. Liu , H. Geng , J. Xu , F. Zan , P. Wang , L. Gu , H. Xia , Adv. Mater. 2021, 33, 2003524. 10.1002/adma.202003524 33336535

[74] Y. Yuan , A. Nie , G. M. Odegard , R. Xu , D. Zhou , S. Santhanagopalan , K. He , H. Asayesh‐Ardakani , D. Desheng Meng , R. F. Klie , C. Johnson , J. Lu , R. Shahbazian‐Yassar , Nano Lett. 2015, 15, 2998. 25871572 10.1021/nl5048913

[75] S. Lee , L. Wu , A. S. Poyraz , J. Huang , A. C. Marschilok , K. J. Takeuchi , E. S. Takeuchi , M. Kim , Y. Zhu , Adv. Mater. 2017, 19, 1703186. 10.1002/adma.201703186 28985007

[76] R. Caia , S. Guo , Q. Meng , S. Yang , H. Xin , X. Hu , M. Li , Y. Sun , P. Gao , S. Zhang , H. Dong , S. Lei , K. Kim , H. Zeng , L. Sun , F. Xu , Y. Zhu , Nano Energy 2019, 63, 103840.

[77] K. He , Y. Yuan , W. Yao , K. You , M. Dahbi , J. Alami , K. Amine , R. Shahbazian‐Yassar , J. Lu , Angew. Chem., Int. Ed. 2022, 134, 202113420. 10.1002/anie.202113420 34699672

[78] J. Zeng , L. Yang , R. Shao , L. Zhou , W. Utetiwabo , S. Wang , R. Chen , W. Yang , J. Colloid Interface Sci. 2021, 600, 111. 34010768 10.1016/j.jcis.2021.04.136

[79] J. Miranda , E. Le Calvez , R. Retoux , O. Crosnier , T. Brousse , Electrochem. Commun. 2022, 137, 107249.

[80] O. A. Drozhzhin , V. Grigoryev , A. M. Alekseeva , R. Samigullin , D. A. Aksyonov , O. V. Boytsova , D. Chernyshov , V. Shapovalov , A. Guda , A. V. Soldatov , K. J. Stevenson , A. M. Abakumov , E. V. Antipov , ACS Appl. Mater. Interfaces 2021, 13, 56366. 34784712 10.1021/acsami.1c20842

[81] Y. Yang , J. Zhao , Adv. Sci. 2021, 8, 2004855. 10.1002/advs.202004855 PMC8224428 34165894

[82] G. A. Horrocks , A. Parija , L. R. De Jesus , L. Wangoh , S. Sallis , Y. Luo , J. L. Andrews , J. Jude , C. Jaye , D. A. Fischer , D. Prendergast , L. F. J. Piper , S. Banerjee , Chem. Mater. 2017, 29, 10386.

[83] Y. Luo , S. Rezaei , D. A. Santos , Y. Zhang , J. V. Handy , L. Carrillo , B. J. Schultz , L. Gobbato , M. Pupucevskie , K. Wiaderek , H. Charalambous , A. Yakovenko , M. Pharr , B. Xu , S. Banerje , Proc. Natl. Acad. Sci. USA 2022, 119, 2115072119. 10.1073/pnas.2115072119 PMC8795564 35064084

[84] J. V. Handy , Y. Luo , J. L. Andrews , N. Bhuvanesh , S. Banerjee , Angew. Chem., Int. Ed. 2020, 59, 16385. 10.1002/anie.202005513 32542874

[85] S. Yang , R. Li , Z. Nie , H. Zhang , Y. Zhang , J. Zhu , Inorg. Chem. Front. 2022, 9, 5579.

[86] T. Ding , J. Xu , C. Chen , Z. Luo , J. Dai , Y. Tian , C. Chen , J. Mater. Sci. Technol. 2017, 33, 271.

[87] Y. Luo , J. V. Handy , T. Das , J. D. Ponis , R. Albers , Y. Chiang , M. Pharr , B. J. Schultz , L. Gobbato , D. C. Brown , S. Chakraborty , S. Banerjee , Nat. Mater. 2024, 23, 960. 38514846 10.1038/s41563-024-01842-y

[88] J. C. Pérez‐Flores , A. Kuhn , F. García‐Alvarado , J. Power Sources 2011, 196, 1378.

[89] F. Liu , Y. Zou , H. Wang , Z. Wang , M. Zhang , W. Wu , D. Du , W. Zhao , T. Zhao , Y. Liu , N. Yao , Y. Ma , ACS Nano 2022, 15, 9117. 10.1021/acsnano.2c01200 35593703

[90] S. R. Bruno , C. K. Blakely , C. M. Tenbusch , V. Poltavets , ECS Trans. 2013, 45, 23.

[91] K. Zhao , C. Sun , Y. Yu , Y. Dong , C. Zhang , C. Wang , P. M. Voyles , L. Mai , X. Wang , ACS Appl. Mater. Interfaces 2018, 10, 44376. 30489060 10.1021/acsami.8b13376

[92] L. Shen , S. Chen , J. Maier , Y. Yu , Adv. Mater. 2017, 29, 1701571. 10.1002/adma.201701571 28640524

[93] W. Xu , Z. Jiang , Q. Yang , W. Huo , M. S. Javed , Y. Li , L. Huang , X. Gu , C. Hu , Nano Energy 2018, 43, 168.

[94] H. M. Abuzeid , A. M. Hashem , M. Kaus , M. Knapp , S. Indris , H. Ehrenberg , A. Mauger , C. M. Julien , J. Alloys Compd. 2018, 746, 227.

[95] Y. Yang , B. Wang , J. Zhu , J. Zhou , Z. Xu , L. Fan , J. Zhu , R. Podila , A. M. Rao , B. Lu , ACS Nano 2016, 10, 5516. 27139149 10.1021/acsnano.6b02036

[96] Z. Li , X. Lian , M. Wu , F. Zheng , Y. Gao , H. Niu , Dalton Trans. 2020, 49, 6644. 32367093 10.1039/d0dt00980f

[97] A. Xia , C. Zhao , W. Yu , Y. Han , J. Yi , G. Tan , J. Appl. Electrochem. 2020, 50, 733.

[98] G. Zhong , J. Yu , P. Zhuang , M. Jin , Y. Fu , X. Ma , Electrochim. Acta 2019, 296, 276.

[99] J. Zhou , S. Lin , Y. Huang , P. Tong , B. Zhao , X. Zhu , Y. Sun , Chem. Eng. J. 2019, 373, 203.

[100] Y. Zhang , J. Li , Z. Gong , J. Xie , T. Lu , L. Pan , J. Colloid Interface Sci. 2021, 587, 489. 33387843 10.1016/j.jcis.2020.12.044

[101] D. Liu , L. Wang , Y. He , L. Liu , Z. Yang , B. Wang , Q. Xia , Q. Hu , A. Zhou , Energy Technol. 2020, 9, 2000753.

[102] C. S. Liu , X. Ye , B. Zhou , X. Q. Zeng , J. Xu , Q. C. Xu , J. Li , J. Phys. Chem. C 2020, 124, 24073.

[103] J. Wang , Z. Liu , W. Yang , L. Han , M. Wei , Chem. Commun. 2018, 54, 7346. 10.1039/c8cc03875a 29911212

[104] Y. Bai , Y. Tang , L. Liu , X. Li , Y. Gao , ACS Sustainable Chem. Eng. 2018, 6, 14614.

[105] Y. Song , W. Zhao , N. Wei , L. Zhang , F. Ding , Z. Liu , J. Sun , Nano Energy 2018, 53, 432.

[106] J. H. Kim , Y. S. Kim , S. H. Moon , D.‐H. Park , M.‐C. Kim , J. H. Choi , J.‐H. Shin , K. W. Park , Electrochim. Acta 2021, 389, 138685.

[107] Y. Tian , G. Wang , L. Zhu , H. Chen , T. Sun , Mater. Today Commun. 2021, 28, 102624.

[108] L. Jiang , Y. Qu , Z. Ren , P. Yu , D. Zhao , W. Zhou , L. Wang , H. Fu , ACS Appl. Mater. Interfaces 2015, 7, 1595. 25569599 10.1021/am5070393

[109] B. Mandal , I. Basumallick , S. Ghosh , Int. Res. J. Pure Appl. Chem. 2015, 5, 30.

[110] Z. Zhang , M. Avdeev , H. Chen , W. Yin , W. H. Kan , G. He , Nat. Commun. 2022, 13, 7790. 36526618 10.1038/s41467-022-35376-1 PMC9758126

[111] X. He , L. Tian , M. Qiao , J. Zhang , W. Geng , Q. Zhang , J. Mater. Chem. A 2019, 7, 11478.

[112] M. Zhang , J. Alloys Compd. 2015, 648, 134.

[113] H. Peng , F. Xia , C. Zhang , H. Zhuo , X. Peng , P. Song , C. Sun , J. Wu , Adv. Funct. Mater. 2022, 32, 2113424.

[114] D. J. Arnot , M. N. Vila , E. S. Takeuchi , A. C. Marschilok , K. J. Takeuchi , J. Electrochem. Soc. 2024, 171, 010524.

[115] A. S. Poyraz , J. Huang , S. Cheng , L. Wu , X. Tong , Y. Zhu , A. C. Marschilok , K. J. Takeuchi , E. S. Takeu , J. Electrochem. Soc. 2017, 164, A1983.

[116] N. I. Cool , R. James , P. Schofield , J. V. Handy , M. Bhatia , S. Banerjee , ACS Appl. Mater. Interfaces 2023, 15, 1554. 36541932 10.1021/acsami.2c17800

[117] L. Ma , H. Cui , S. Chen , X. Li , B. Dong , C. Zhi , Nano Energy 2021, 81, 105632.

[118] H. Park , S. Jo , T. Song , U. Paik , Cryst. Growth Des. 2020, 20, 4749.

[119] Y. Wang , K. Xie , Y. Zhu , K. Tong , M. Zhang , F. Wu , J. Power Sources 2023, 577, 233234.

[120] C. Wu , J. Hu , H. Chen , C. Zhang , M. Xu , L. Zhuang , X. Ai , J. Qian , Energy Storage Mater. 2023, 60, 102803.

[121] P. Barnes , Y. Zuo , K. Dixon , D. Hou , S. Lee , Z. Ma , J. G. Connell , H. Zhou , C. Deng , K. Smith , E. Gabriel , Y. Liu , O. Maryon , P. H. Davis , H. Zhu , Y. Du , J. Qi , Z. Zhu , C. Chen , Z. Zhu , Y. Zhou , P. J. Simmonds , A. E. Briggs , D. Schwartz , S. Ping Ong , H. Xiong , Nat. Mater. 2022, 21, 795. 35501365 10.1038/s41563-022-01242-0

[122] H. Han , Q. Jacquet , Z. Jiang , F. N. Sayed , J. Jeon , A. Sharma , A. M. Schankler , A. Kakekhani , H. L. Meyerheim , J. Park , S. Yeol Nam , K. J. Griffith , L. Simonelli , A. M. Rappe , C. P. Grey , S. P. Parkin , Nat. Mater. 2023, 22, 1128. 37500959 10.1038/s41563-023-01612-2 PMC10465368

[123] J. Lai , Z. Zou , Y. Bai , Y. Xing , C. Jiang , Rare Met. 2024, 43, 2053.

[124] Y. Yang , H. Zhu , J. Xiao , H. Geng , Y. Zhang , J. Zhao , G. Li , X. Wang , C. Li , Q. Liu , Adv. Mater. 2020, 32, 1905295. 10.1002/adma.201905295 32077160

[125] Y. Zhang , Y. Ugata , B. L. Campéon , N. Yabuuchi , Adv. Energy Mater. 2024, 14, 2304074.

[126] J. Chen , J. Meng , K. Han , F. Liu , W. Wang , Q. An , L. Mai , Nano Energy 2023, 110, 108377.

[127] Y. Sheng , X. Zhang , D. Liu , L. Zhou , F. Zhou , C. Wei , Y. Wang , G. Wen , Chem. Eng. Sci. 2024, 298, 120356.

[128] K. Parui , A. D. Lee , S. Gandhi , M. Butala , J. Mater. Chem. A 2023, 11, 5559.

[129] X. Yan , T. Li , Y. Xiong , X. Ge , Energy Storage Mater. 2021, 36, 213.

[130] Z. Zhu , Y. Chen , F. Liu , H. Wang , R. Yu , D. He , J. Wu , Electrochim. Acta 2023, 441, 141796.

[131] Z. Song , H. Li , W. Liu , H. Zhang , J. Yan , Y. Tang , J. Huang , H. Zhang , X. Li , Adv. Mater. 2020, 32, 2001001. 10.1002/adma.202001001 32309887

[132] M. Su , Y. Lei , K. He , K. Fu , X. Chen , A. Dou , Y. Zhou , Y. Liu , Electrochim. Acta 2023, 463, 142828.

[133] Y. Liu , W. Zhong , C. Yang , X. Liu , Q. Cheng , T. Tan , Q. Deng , C. Yang , J. Colloid Interface Sci. 2024, 667, 136. 38636215 10.1016/j.jcis.2024.04.035

[134] S. Zhang , J. Hwang , Y. Sato , K. Matsumoto , R. Hagiwara , ACS Appl. Energy Mater. 2023, 6, 2333.

[135] W. Xu , Y. Xu , T. Schultz , Y. Lu , N. Koch , N. Pinna , ACS Appl. Mater. Interfaces 2022, 15, 795. 36542687 10.1021/acsami.2c15124

[136] F. Liu , Z. Zhu , Y. Chen , J. Meng , H. Wang , R. Yu , X. Hong , J. Wu , ACS Appl. Mater. Interfaces 2022, 14, 49865. 10.1021/acsami.2c15697 36308403

[137] X. Li , Y. Chen , H. Wang , H. Yao , H. Huang , Y. W. Mai , N. Hu , L. Zhou , Adv. Funct. Mater. 2015, 26, 376.

[138] S. Zhang , H. Ying , P. Huang , J. Wang , Z. Zhang , T. Yang , W.‐Q. Han , ACS Nano 2020, 14, 17665. 33301296 10.1021/acsnano.0c08770

[139] R. Niu , R. Han , Y. Wang , L. Zhang , Q. Qiao , L. Jiang , Y. Sun , S. Tang , J. Zhu , Chem. Eng. J. 2021, 405, 127049.

[140] Y. He , A. Zhou , D. Liu , Q. Hu , X. Liu , L. Wang , ChemistrySelect 2019, 4, 10694.

[141] N. Xue , X. Li , M. Zhang , L. Han , Y. Liu , X. Tao , ACS Appl. Energy Mater. 2020, 3, 10234.

[142] X. Song , H. Wang , S. Jin , M. Lv , Y. Zhang , X. Kong , H. Xu , T. Ma , X. Luo , H. Tan , D. Hu , C. Deng , X. Chang , J. Xu , Nano Res. 2020, 13, 1659.

[143] R. Cheng , Z. Wang , C. Cui , T. Hu , B. Fan , H. Wang , Y. Liang , C. Zhang , H. Zhang , X. Wang , J. Phys. Chem. C 2020, 124, 6012.

[144] S. Zhang , H. Ying , R. Guo , W. Yang , W.‐Q. Han , J. Phys. Chem. Lett. 2019, 10, 6446. 31589051 10.1021/acs.jpclett.9b02335

[145] Z. Zhang , H. Guo , W. Li , G. Liu , Y. Zhang , Y. Wang , New J. Chem. 2020, 44, 5913.

[146] S. Zhang , H. Liu , B. Cao , Q. Zhu , P. Zhang , X. Zhang , R. Chen , F. Wu , B. Xu , J. Mater. Chem. A 2019, 7, 21766.

[147] J. Zhao , J. Wen , J. Xiao , X. Ma , J. Gao , L. Bai , H. Gao , X. Zhang , Z. Zhang , J. Energy Chem. 2021, 53, 387.

[148] C. Li , Z. Xue , J. Qin , M. Sawangphruk , P. Yu , X. Zhang , R. Liu , J. Alloys Compd. 2020, 842, 156405.

[149] Z. Wang , J. Bai , H. Xu , G. Chen , S. Kang , X. Li , J. Colloid Interface Sci. 2020, 577, 329. 32485414 10.1016/j.jcis.2020.05.035

[150] B. Zhang , J. Zhu , P. Shi , W. Wu , F. Wang , Ceram. Int. 2019, 45, 8395.

[151] D. Sun , M. Wang , Z. Li , G. Fan , L.‐Z. Fan , A. Zhou , Electrochem. Commun. 2014, 47, 80.

[152] C. E. Ren , M. Q. Zhao , T. Makaryan , J. Halim , M. Boota , S. Kota , B. Anasori , M. W. Barsoum , Y. Gogotsi , ChemElectroChem 2016, 3, 689.

[153] J. Luo , X. Tao , J. Zhang , Y. Xia , H. Huang , L. Zhang , Y. Gan , C. Liang , W. Zhang , ACS Nano 2016, 10, 2491. 26836262 10.1021/acsnano.5b07333

[154] A. Kuhn , J. C. Perez‐Flores , M. Hoelzel , C. Baehtz , I. Sobrados , J. Sanz , F. Garcıa‐Alvarado , J. Mater. Chem. A 2018, 6, 443.

[155] Y. Wang , H. Zhang , X. Yao , H. Zhao , ACS Appl. Mater. Interfaces 2013, 5, 1108. 23327096 10.1021/am302907v

[156] P. Li , P. Wang , S. Qian , H. Yu , X. Lin , M. Shui , X. Zheng , N. Long , J. Shu , Electrochim. Acta 2016, 187, 46.

[157] J. Kanchanawarin , W. Limphirat , P. Promchana , T. Sooknoi , T. Maluangnont , K. Simalaotao , A. Boonchun , P. Reunchan , S. Limpijumnong , J. T‐Thienprasert , J. Appl. Phys. 2018, 124, 155101.

[158] X. K. Zhang , J. J. Yuan , H. J. Yu , X. R. Zhu , Z. Yin , H. Shen , Y. M. Xie , J. Alloys Compd. 2015, 631, 171.

[159] L. Yu , W. Zhang , J. Lv , X. Liu , Y. Li , M. Wei , J. Electroanal. Chem. 2020, 874, 114522.

[160] J. Wang , H. Jing , X. Wang , Y. Xue , Q. Liang , W. Qi , H. Yu , C. Du , Adv. Funct. Mater. 2024, 34, 2315318.

[161] X. Wang , Q. Yang , K. Li , M. Zhen , J. Alloys Compd. 2024, 1003, 175676.

[162] D. Wang , Y. Deng , Y. Liu , Y. Jiang , B. Zhong , Z. Wu , X. Guo , Z. Chen , Nano Energy 2023, 110, 108340.

[163] E. Oz , S. Altin , S. Avci , J. Solid State Chem. 2023, 318, 123741.

[164] H. Liu , R. Feng , F. Hussain , Y. Liu , L. Wang , Q. Fan , M. Ni , C. Qiu , M. Sun , J. Wang , T. Wang , Z. Shi , X. Zhu , H. Xia , Adv. Funct. Mater. 2024, 34, 2404442.

[165] S. Chakrabarty , J. A. Dar , A. Joshi , A. Paperni , S. Taragin , A. Maddegalla , G. S. Gautam , A. Mukherjee , M. Noked , J. Mater. Chem. A 2024, 12, 25109.

[166] Y. Yuana , L. Ma , K. He , W. Yao , A. Nie , X. Bi , K. Amine , T. Wu , J. Lub , R. Shahbazian‐Yassr , Nano Energy 2016, 19, 382.

[167] C. Gu , E. Zhao , N. Li , K. Gao , K. Wu , P. Ran , M. Fu , Q. Wu , J. Zhao , Y. Wang , Appl. Phys. Lett. 2024, 125, 053904.

[168] J. Wang , Q. Sun , J. Yu , J. Guo , N. Mo , H. Li , Y. Su , S. Zhao , Y. Zhu , H. Chu , S. Dou , Y. Xiao , Composites, Part B 2024, 284, 111664.

[169] R. Cai , S. Guo , Y. Wu , S. Zhang , Y. Sun , S. Chen , P. Gao , C. Zhu , J. Chen , Z. Zhu , L. Sun , F. Xu , Energy Storage Mater. 2021, 37, 345.

[170] B. W. Byles , E. Pomerantseva , Materialia 2021, 15, 101013.

[171] F. Gu , T. Sun , X. Yao , M. Shui , J. Shu , J. Phys. Chem. Solids 2021, 149, 109771.

[172] Z. Hua , Y. Jian , J. Wang , Y. Lin , W. Zhou , H. Jiang , Y. Shen , X. Wu , Y. Xiang , J. Solid State Chem. 2024, 329, 124415.

[173] W. Shi , H. Li , D. Zhang , F. Du , Y. Zhang , Z. Wang , X. Zhang , P. Zhang , Chem. Eng. J. 2023, 477, 146976.

[174] Z. Jian , Y. Liu , Y. Zhu , J. Li , H. Hu , J. Wang , L. Kong , X. Jia , H. Liu , J. Guo , M. Li , Y. Xu , J. Mao , S. Zhang , Y. Su , S. Dou , S. Chou , Y. Xiao , Nano Energy 2024, 125, 109528.

[175] X. Chen , H. Liu , M. Zhou , G. Fang , H. Zhang , Z. Cai , X. Zhao , L. Xiao , S. Liu , Y. Zhang , Electrochim. Acta 2022, 401, 139522.

[176] B. Mandal , S. Chakrabarti , A. K. Thakur , Comput. Mater. Sci. 2024, 238, 112934.

[177] C. Wu , W. Hua , Z. Zhang , B. Zhong , Z. Yang , G. Feng , W. Xiang , Z. Wu , X. Guo , Adv. Sci. 2018, 5, 1800519. 10.1002/advs.201800519 PMC6145307 30250795

[178] Q. Zhang , Y. Guo , K. Guo , T. Zhai , H. Li , Chem. Commun. 2016, 52, 6229. 10.1039/c6cc01057a 26980665

[179] K. Cao , L. Jiao , W. Pang , H. Liu , T. Zhou , Z. Guo , Y. Wang , H. Yuan , Small 2016, 12, 2991. 27095282 10.1002/smll.201600845

[180] Y. Cai , J. Zhou , G. Fang , G. Cai , A. Pan , S. Liang , J. Power Sources 2016, 328, 241.

[181] W. Ko , J. Yoo , H. Park , Y. Lee , I. Kang , J. Kang , J. Hyeon Jo , J. Ung Choi , J. Hong , S. Myung , J. Kim , Nano Energy 2020, 77, 105175.

[182] E. Adamczyk , M. Gnanavel , V. Pralong , Materials 2018, 11, 1021. 29914070 10.3390/ma11061021 PMC6024919

[183] F. Liu , H. Xu , Y. He , H. Bian , D. Li , A. Wang , D. Sun , Energy Fuels 2023, 37, 11355.

[184] S. Osman , S. Zuo , X. Xu , J. Shen , Z. Liu , F. Li , P. Li , X. Wang , J. Liu , ACS Appl. Mater. Interfaces 2021, 13, 816. 33395248 10.1021/acsami.0c21328

[185] J. Ni , W. Wang , C. Wu , H. Liang , J. Maier , Y. Yu , L. Li , Adv. Mater. 2017, 29, 1. 10.1002/adma.201605607 28026059

[186] F. Liu , X. Cheng , R. Xu , Y. Wu , Y. Jiang , Y. Yu , Adv. Funct. Mater. 2018, 28, 1800394.

[187] Y. L. Han , M. H. Yang , Y. Zhang , J. J. Xie , D. G. Yin , C. L. Li , Chem. Mater. 2016, 28, 3139.

[188] L. Yan , G. Chen , S. Sarker , S. Richins , H. Wang , W. Xu , X. Rui , H. Luo , ACS Appl. Mater. Interfaces 2016, 8, 22213. 27508452 10.1021/acsami.6b06516

[189] Y. L. Wu , X. Fan , Y. J. Chen , R. R. Gaddam , F. Yu , C. L. Xiao , C. F. Lin , Q. L. Zhao , X. M. Sun , H. X. Wang , C. G. Liu , J. Li , X. S. Zhao , J. Mater. Chem. A 2019, 7, 20813.

[190] C. Yu , Y. Lin , Y. Wang , J. Zhang , C. Xia , J. Cui , J. Liu , Y. Zhang , H. H. Tan , Y. Wu , J. Colloid Interface Sci. 2025, 684, 403. 39799623 10.1016/j.jcis.2025.01.018

[191] Y. Li , H. Wang , L. Wang , Z. Mao , R. Wang , B. He , Y. Gong , X. Hu , Small 2019, 15, 1804539. 10.1002/smll.201804539 30701686

[192] J. Ma , J. Qin , S. Zheng , Y. Fu , L. Chi , Y. Li , C. Dong , B. Li , F. Xing , H. Shi , Z.‐S. Wu , Nano‐Micro Lett. 2024, 16, 2. 10.1007/s40820-023-01281-5 PMC10766898 38175485

[193] Z. Tong , S. Liu , Y. Zhou , J. Zhao , Y. Wu , Y. Wang , Y. Li , Energy Storage Mater. 2018, 13, 223.

[194] D. Luo , C. Ma , J. Hou , Z. Zhang , R. Feng , L. Yang , X. Zhang , H. Lu , J. Liu , Y. Li , Y. Zhang , X. Wang , Z. Chen , Adv. Energy Mater. 2022, 12, 2103716.

[195] B. Liu , S. Hu , Y. Pan , F. Zeng , S. Zhou , Y. Zheng , Y. Ma , D. Ma , S. Luo , Small 2023, 20, 2308263. 10.1002/smll.202308263 37946672

[196] Z. Lv , H. Xu , W. Xu , B. Peng , C. Zhao , M. Xie , X. Lv , Y. Gao , K. Hu , Y. Fang , W. Dong , F. Huang , Adv. Energy Mater. 2023, 13, 2300790.

[197] W. Wang , S. a. He , Z. Cui , Q. Liu , M. F. Yuen , J. Zhu , H. Wang , M. Gao , W. Luo , J. Hu , R. Zou , Small 2022, 18, 2203948. 10.1002/smll.202203948 36084223

[198] Y. Zhao , Z. Feng , Y. Tan , Q. Deng , L. Yao , Nanomaterials 2024, 14, 14070631. 10.3390/nano14070631 PMC11013061 38607165

[199] S. Wu , T. Bashir , Y. Zhang , L. Gao , J. Solid State Electrochem. 2023, 29, 61.

[200] X. Shi , J. Li , X. Lang , Q. Jiang , J. Phys. Chem. C 2017, 121, 5974.

[201] X. Jia , R. Tian , C. Liu , J. Zheng , M. Tian , G. Cao , Mater. Today Energy 2022, 28, 101063.

[202] A. S. Etmana , J. Suna , R. Younesi , J. Energy Chem. 2019, 30, 145.

[203] H. Li , H. Xu , C. Wang , X. Yang , L. Li , J. Sheng , Y. Jin , M. Wang , Y. Liu , Y. Zou , D. Yang , Chem. Eng. J. 2024, 479, 147597.

[204] R. Baddour‐Hadjean , M. Safrany Renard , J. Pereira‐Ramos , J. Power Sources 2021, 482, 229017.

[205] R. Cordoba , O. Dolotko , A. Kuhn , F. García‐Alvarado , J. Alloys Compd. 2024, 1002, 175512.

[206] J. Peng , W. Zhang , Z. Hu , L. Zhao , C. Wu , G. Peleckis , Q. Gu , J. Wang , H. Liu , S. Dou , S. Chou , Nano Lett. 2022, 22, 1302. 35089723 10.1021/acs.nanolett.1c04492

[207] Q. Li , C. Xu , Y. Liang , Z. Yang , N. LeGe , J. Peng , L. Chen , W. Lai , Y. Wang , Z. Tao , M. Liu , S. Chou , ACS Appl. Mater. Interfaces 2022, 14, 47747. 36250578 10.1021/acsami.2c13639

[208] J. Peng , W. Hua , Z. Yang , J. Li , J. Wang , Y. Liang , L. Zhao , W. Lai , X. Wu , Z. Cheng , G. Peleckis , S. Indris , J. Wang , H. Liu , S. Dou , S. Chou , ACS Nano 2024, 18, 19854. 10.1021/acsnano.4c07021 39007545

[209] B. Ran , R. Cheng , Y. Zhong , X. Zhang , T. Zhao , Z. Yang , C. Yang , J. Zhang , C. Fu , Energy Storage Mater. 2024, 71, 103583.

[210] X. Xu , Y. Lan , B. Zhang , S. Zhu , Y. Yang , Y. Gao , Electrochim. Acta 2023, 471, 143375.

[211] L. Ge , Y. Song , P. Niu , B. Li , L. Zhou , W. Feng , C. Ma , X. Li , D. Kong , Z. Yan , Q. Xue , Y. Cui , W. Xing , ACS Nano 2024, 18, 3542. 38215406 10.1021/acsnano.3c11169

[212] Y. He , S. L. Dreyer , T. Akçay , T. Diemant , R. Mönig , Y. Ma , Y. Tang , H. Wang , J. Lin , S. Schweidler , M. Fichtner , H. Hahn , T. Brezesinski , B. Breitung , Y. Ma , ACS Nano 2024, 18, 24441. 39172962 10.1021/acsnano.4c07528

[213] Y. Zhang , J. Huang , L. Qiu , R. Jiao , Y. Zhang , G. Yang , L. Zhang , Z. Tian , E. Debroye , T. Liu , J. Gohy , J. Hofkens , F. Lai , ACS Appl. Mater. Interfaces 2024, 16, 27684. 38753436 10.1021/acsami.4c04785

[214] M. Qin , W. Ren , R. Jiang , Q. Li , X. Yao , S. Wang , Y. You , L. Mai , ACS Appl. Mater. Interfaces 2021, 13, 3999. 33439613 10.1021/acsami.0c20067

[215] A. Duarte‐Cardenas , P. Díaz‐Carrasco , A. Kuhn , A. Basa , Electrochim. Acta 2022, 427, 140872.

[216] T. Yao , H. Wang , Y. Qin , J. W. Shi , Y. Cheng , Composites, Part B 2023, 253, 110557.

[217] Y. Xiao , Y. Kong , X. Wang , H. Luo , G. Yuan , S. Zhang , A. Zhang , J. Zhou , Y. Fan , L. Xin , A. Wang , S. Fang , Y. Zheng , J. Colloid Interface Sci. 2025, 677, 577. 39111093 10.1016/j.jcis.2024.07.245

[218] Y. E. Zhu , L. Yang , J. Sheng , Y. Chen , H. Gu , J. Wei , Z. Zhou , Adv. Energy Mater. 2017, 7, 1701222.

[219] T. Zhang , X. Shi , Z. Mao , C. Luo , G. Li , R. Wang , B. He , J. Jin , Y. Gong , H. Wang , Electrochim. Acta 2021, 399, 139377.

[220] S. Dong , L. Shen , H. Li , G. Pang , H. Dou , X. Zhang , Adv. Funct. Mater. 2016, 26, 3703.

[221] E. Lim , C. Jo , M. S. Kim , M. H. Kim , J. Chun , H. Kim , J. Park , K. C. Roh , K. Kang , S. Yoon , J. Lee , Adv. Funct. Mater. 2016, 26, 3711.

[222] H. Yang , R. Xu , Y. Gong , Y. Yao , L. Gu , Y. Yu , Nano Energy 2018, 48, 448.

[223] L. Wang , X. Bi , S. Yang , Adv. Mater. 2016, 28, 7672. 27346391 10.1002/adma.201601723

[224] Q. Deng , L. Yao , Coatings 2022, 12, 12121873.

[225] Q. Zhang , Y. Wei , H. Yang , D. Su , Y. Ma , H. Li , T. Zha , ACS Appl. Mater. Interfaces 2017, 9, 7009. 28157289 10.1021/acsami.6b13869

[226] D. Tao , Z. Fang , M. Qiu , Y. Li , X. Huang , K. Ding , W. Chen , W. Su , Y. Zhang , J. Alloys Compd. 2016, 689, 805.

[227] A. Vasileiadis , M. Wagemaker , Chem. Mater. 2017, 29, 1076.

[228] X. Rui , Y. Tang , O. I. Malyi , A. Gusak , Y. Zhang , Z. Niu , H. Tan , C. Persson , X. Chen , Z. Chen , Q. Yan , Nano Energy 2016, 22, 583.

[229] S. Chong , Y. Wu , C. Liu , Y. Chen , S. Guo , Y. Liu , G. Cao , Nano Energy 2018, 54, 106.

[230] X. Wang , F. Zhang , C. Xia , L. Cui , F. Yang , J. Alloys Compd. 2024, 970, 172599.

[231] Y. Zhu , X. Yang , T. Sun , S. Wang , Y. Zhao , J. Yan , X. Zhang , Electrochem. Energy Rev. 2018, 1, 548.

[232] H. Tian , X. Yu , H. Shao , L. Dong , Y. Chen , X. Fang , C. Wang , W. Han , G. Wang , Adv. Energy Mater. 2019, 9, 1901560.

[233] R. Rajagopalan , Y. Tang , X. Ji , C. Jia , H. Wang , Adv. Funct. Mater. 2020, 30, 1909486.

[234] W. Zhang , J. Yin , W. Wang , Z. Bayhan , H. N. Alshareef , Nano Energy 2021, 83, 105792.

[235] S. Vanam , B. Senthilkumar , P. Amonpattaratkit , P. Barpanda , Inorg. Chem. 2022, 61, 3959. 35201758 10.1021/acs.inorgchem.1c03609

[236] J. Hyeon Jo , H. Jae Kim , N. Yaqoob , K. Ihm , O. Guillon , K. Sohn , N. Lee , P. Kaghazchi , S. Myung , Energy Storage Mater. 2023, 54, 680.

[237] J. Cong , S. Luo , K. Li , S. Yan , Q. Wang , Y. Zhang , X. Liu , J. Electroanal. Chem. 2022, 927, 116971.

[238] B. Pandit , S. R. Rondiy , S. F. Shaikh , M. Ubaidullah , R. Amaral , N. Y. Dzade , E. S. Goda , A. Hassan Sarwar Rana , H. Singh Gill , T. Ahmad , J. Colloid Interface Sci. 2023, 633, 886. 36495810 10.1016/j.jcis.2022.11.070

[239] K. Cao , H. Liu , W. Li , C. Xu , Q. Han , Z. Zhang , L. Jiao , J. Electroanal. Chem. 2019, 841, 51.

[240] J. Hu , Y. Xie , J. Zheng , H. Li , T. Wang , Y. Lai , Z. Zhang , ACS Appl. Mater. Interfaces 2021, 13, 12149. 33656850 10.1021/acsami.1c01303

[241] H. He , K. Cao , S. Zeng , J. Si , Y. Zhu , C. Chen , J. Power Sources 2023, 587, 233715.

[242] Q. Zhou , H. Liu , S. Dou , S. Chong , ACS Nano 2024, 18, 7287. 38373205 10.1021/acsnano.4c00251

[243] Q. Zhang , C. Didier , W. K. Pang , Y. Liu , Z. Wang , S. Li , V. K. Peterson , J. Mao , Z. Guo , Adv. Energy Mater. 2019, 9, 1900568.

[244] T. Jin , H. Li , Y. Li , L. Jiao , J. Chen , Nano Energy 2018, 50, 462.

[245] Q. Deng , L. Wang , J. Li , Q. Cheng , X. Liu , C. Chen , Q. Zhang , W. Zhong , H. Wang , L. Wu , C. Yang , Chin. Chem. Lett. 2023, 34, 2203095.

[246] G. Yao , M. Lin , J. Yang , L. Wei , H. niu , Q. Luo , F. Zheng , Q. Chen , Inorg. Chem. Front. 2022, 9, 1434.

[247] Y. Wu , P. Wu , Y. Tang , R. Fu , Y. Cui , J. Chen , C. Kübel , F. Xu , Adv. Funct. Mater. 2024, 34, 2314344.

[248] L. Wu , H. Fu , S. Li , J. Zhu , J. Zhou , A. M. Rao , L. Cha , K. Guo , S. Wen , B. Lu , Nat. Commun. 2023, 14, 644. 36746953 10.1038/s41467-023-36385-4 PMC9902589

[249] S. Dong , N. Lv , R. Ren , Y. Wu , P. Liu , G. Zhu , W. Wang , Y. Zhang , X. Dong , Sci. China Mater. 2022, 65, 3069.

[250] M. Huang , X. Wang , J. Meng , X. Liu , X. Yao , Z. Liu , L. Mai , Nano Energy 2020, 77, 105069.

[251] L. Duan , H. Tang , X. Xu , J. Liao , X. Li , G. Zhou , X. Zhou , Energy Storage Mater. 2023, 62, 102950.

[252] S. Chong , J. Yang , L. Sun , S. Guo , Y. Liu , H. Liu , ACS Nano 2020, 14, 9807. 32709197 10.1021/acsnano.0c02047

[253] H. Chen , Z. Wu , Z. Zheng , T. Chen , X. Guo , J. Li , B. Zhong , Electrochim. Acta 2018, 273, 63.

[254] G. He , L. F. Nazar , ACS Energy Lett. 2017, 2, 1122.

[255] Y. Xia , W. Jin , Y. Qi , H. Li , Z. Jian , W. Chen , Chin. Chem. Lett. 2021, 32, 2433.

[256] W. Shu , C. Han , X. Wang , Adv. Funct. Mater. 2023, 34, 2309636.

[257] C. Zhang , Y. Xu , M. Zhou , L. Liang , H. Dong , M. Wu , Y. Yang , Y. Lei , Adv. Funct. Mater. 2016, 27, 1604307.

[258] X. Wu , Z. Jian , Z. Li , X. Ji , Electrochem. Commun. 2017, 77, 54.

[259] Z. Shadike , D.‐R. Shi , T.‐W. Tian‐Wang , M.‐H. Cao , S.‐F. Yang , J. Chen , Z.‐W. Fu , J. Mater. Chem. A 2017, 5, 6393.

[260] Y. h. Zhu , Y. b. Yin , X. Yang , T. Sun , S. Wang , Y. s. Jiang , J. m. Yan , X. b. Zhang , Angew. Chem., Int. Ed. 2017, 56, 7881. 10.1002/anie.201702711 28466484

[261] S. Chong , Y. Chen , Y. Zheng , Q. Tan , C. Shu , Y. Liu , Z. Guo , J. Mater. Chem. A 2017, 5, 22465.

[262] J. Liao , Q. Hu , Y. Yu , H. Wang , Z. Tang , Z. Wen , C. Chen , J. Mater. Chem. A 2017, 5, 19017.

[263] Y. Pei , C. Mu , H. Li , F. Li , J. Chen , ChemSusChem 2018, 11, 1285. 29498226 10.1002/cssc.201800057

[264] Y.‐H. Zhu , X. Yang , D. Bao , X. F. Bie , T. Sun , S. Wang , Y. S. Jiang , X.‐B. Zhang , J.‐M. Yan , Q. Jiang , Joule 2018, 2, 736.

[265] Q. Xue , L. Li , Y. Huang , R. Huang , F. Wu , R. Chen , ACS Appl. Mater. Interfaces 2019, 11, 22339. 31149796 10.1021/acsami.9b04579

[266] M. Qin , W. Ren , J. Meng , X. Wang , X. Yao , Y. Ke , Q. Li , L. Mai , ACS Sustainable Chem. Eng. 2019, 7, 11564.

[267] G. Oh , S. Kansara , X. Xu , Y. Liu , S. Xiong , J. Hwang , Adv. Funct. Mater. 2024, 34, 2401210.

[268] Q. Deng , L. Wang , J. Li , Q. Cheng , X. Liu , C. Chen , Q. Zhang , W. Zhong , H. Wang , L. Wu , C. Yang , Chin. Chem. Lett. 2023, 34, 107372.

[269] Y. Zhu , Q. Zhang , X. Yang , E. Zhao , T. Sun , X. Zhang , S. Wang , X. Yu , J. Yan , Q. Jiang , Chem 2019, 5, 168.

[270] B. Liu , X. Shi , X. Lang , L. Gu , Z. Wen , M. Zhao , Q. Jiang , Nat. Commun. 2018, 9, 1375. 29636459 10.1038/s41467-018-03700-3 PMC5893573

[271] Q. Deng , Y. Zhao , X. Zhu , K. Yang , M. Li , Nanomaterials 2023, 13, 2539. 37764568 10.3390/nano13182539 PMC10534337

[272] F. Wang , X. Liu , J. Mao , ACS Appl. Mater. Interfaces 2023, 15, 55848. 38013450 10.1021/acsami.3c13303

[273] S. Kumar , R. Sharma , S. Dubey , M. Gupta , S. Natarajan , S. Kumar , J. Power Sources 2024, 623, 235408.

[274] Y. Wang , F. Zhang , Q. Long , S. Li , D. Guo , Z. Zhu , H. Zhang , Energy Storage Mater. 2024, 71, 103399.

[275] A. Li , Y. Man , J. Liao , L. Duan , X. Ji , X. Zhou , Nano Lett. 2023, 23, 10066. 37846924 10.1021/acs.nanolett.3c03558

[276] J. Cai , R. Cai , Z. Sun , X. Wang , N. Wei , F. Xu , Y. Shao , P. Gao , S. Dou , J. Sun , Nano‐Micro Lett. 2020, 12, 2. 10.1007/s40820-020-00460-y PMC7770745 34138148

[277] D. P. Dubal , A. Schneemann , V. Ranc , Š. Kment , O. Tomanec , M. Petr , H. Kmentova , M. Otyepka , R. Zboˇril , R. A. Fischer , K. Jayaramulu , Adv. Energy Sustainability Res. 2021, 2, 2100042.

[278] J. Cong , S. Luo , Y. C. Lin , P.‐y. Li , L. X. Qian , S. X. Yan , J. Guo , J. Energy Storage 2024, 102, 114017.

[279] Y. Dong , Z. S. Wu , S. Zheng , X. Wang , J. Qin , S. Wang , X. Shi , X. Bao , ACS Nano 2017, 11, 4792. 28460161 10.1021/acsnano.7b01165

[280] J. Han , M. Xu , Y. Niu , G.‐N. Li , M. Wang , Y. Zhang , M. Jia , C. m. Li , Chem. Commun. 2016, 52, 11274. 10.1039/c6cc05102b 27709176

[281] S. Dong , Z. Li , Z. Xing , X. Wu , X. Ji , X. Zhang , ACS Appl. Mater. Interfaces 2018, 10, 15542. 29683638 10.1021/acsami.7b15314

[282] H. Li , X. Sun , H. Gou , C. Zhang , G. Wang , J. Colloid Interface Sci. 2023, 638, 161. 36736117 10.1016/j.jcis.2023.01.085

[283] Y. Li , C. Yang , F. Zheng , Q. Pan , Y. Liu , G. Wang , T. Liu , J. Hu , M. Liu , Nano Energy 2019, 59, 582.

[284] S. S. Fan , H. P. Liu , Q. Liu , C. S. Ma , T. F. Yi , J. Materiomics 2020, 6, 431.

[285] Z. Wei , D. Wang , M. Li , Y. Gao , C. Wang , G. Chen , F. Du , Adv. Energy Mater. 2018, 8, 1801102.

[286] J. Cai , R. Cai , Z. Sun , X. Wang , N. Wei , F. Xu , Y. Shao , P. Gao , S. Dou , J. Sun , Nano‐Micro Lett. 2020, 12, 123. 10.1007/s40820-020-00460-y PMC7770745 34138148

[287] M. Sha , L. Liu , H. Zhao , Y. Lei , Carbon Energy 2020, 2, 350.

[288] M. Sakao , N. Kijima , J. Akimoto , T. Okutani , Solid State Ionics 2013, 243, 22.

[289] P. Li , W. Wang , S. Gong , F. Lv , H. Huang , M. Luo , Y. Yang , C. Yang , J. Zhou , C. Qian , B. Wang , Q. Wang , S. Guo , ACS Appl. Mater. Interfaces 2018, 10, 37974. 30207451 10.1021/acsami.8b11354

[290] J. Ma , Y. Li , X. Wei , Z. Li , G. Li , T. Liu , Y. Zhao , S. He , Y. Li , R. Li , C. Gu , J. Li , H. Luo , Q. Wang , K. Li , C. Liu , Chem. Eng. J. 2022, 433, 133777.

[291] X. Chen , C. Hua , K. Zhang , H. Sun , S. Hu , Z. Jian , ACS Appl. Mater. Interfaces 2023, 15, 47125. 37756438 10.1021/acsami.3c11278

[292] Z. Zhang , Y. Ni , M. Avdeev , W. H. Kan , G. He , Electrochim. Acta 2021, 365, 137376.

[293] X. Wu , W. Deng , J. Qian , Y. Cao , X. Ai , H. Yang , J. Mater. Chem. A 2013, 10130.

[294] Y. You , X.‐L. Wu , Y.‐X. Yin , Y.‐G. Guo , Energy Environ. Sci. 2014, 7, 1643.

